# The global burden of bladder, kidney, and prostate cancers attributable to smoking from 1990 to 2021 and projections for the next two decades: A cross-sectional study

**DOI:** 10.18332/tid/204299

**Published:** 2025-05-23

**Authors:** Xiangyu Chen, Xuexue Hao, Jinhao Wu, Xiaoqiang Liu

**Affiliations:** 1 Department of Urology, Tianjin Medical University General Hospital, Tianjin, China

**Keywords:** Global Burden of Disease, urological cancers, smoking, tobacco control

## Abstract

**INTRODUCTION:**

Smoking increases the risk of bladder and kidney cancers and is associated with a poorer prognosis in prostate cancer (PCa) patients, which poses a significant health and socioeconomic burden. Understanding the epidemiologic trends of urological cancers attributable to smoking is critical to developing targeted prevention strategies. This study examines global trends in the three urological cancers attributable to smoking from 1990 to 2021 and projects future trends over the next two decades.

**METHODS:**

Data were obtained from the Global Burden of Disease (GBD) 2021. Metrics included deaths, disability-adjusted life years (DALYs), age-standardized mortality rates (ASMR), and age-standardized DALY rates (ASDR), with uncertainty intervals (UIs). Burden comparisons were stratified by sex, age, and sociodemographic index (SDI). Temporal trends were analyzed using Joinpoint regression to calculate annual percentage change (APC) and average annual percentage change (AAPC), with 95% confidence intervals (CIs). Future trends were predicted using the autoregressive integrated moving average (ARIMA) model.

**RESULTS:**

Compared with 1990, the number of deaths of bladder cancer, kidney cancer and PCa attributable to smoking increased by 43%, 67%, and 31%, and the number of DALYs increased by 31%, 52%, and 29% in 2021. However, the corresponding age-standardized rates showed a downward trend (AAPC_ASMR of bladder cancer_, -1.53; AAPC_ASDR of bladder cancer_, -1.68; AAPC_ASMR of kidney cancer_, -0.89; AAPC_ASDR of kidney cancer_, -1.11; AAPC_ASMR of PCa_, -2.10; AAPC_ASDR of PCa_, -1.97). The burden was higher among males than females, with the highest burden observed in high-SDI regions. The ASMR and ASDR were found to have a non-linear positive correlation with SDI (RASMR of bladder cancer=0.574, p<0.001; R_ASDR of bladder cancer_=0.580, p<0.001; R_ASMR of kidney cancer_=0.792, p<0.001; R_ASDR of kidney cancer_=0.783, p<0.001; R_ASMR of PCa_=0.417, p<0.001; R_ASDR of PCa_=0.436, p<0.001), although the greatest improvements over the past three decades were observed in high-SDI regions. Joinpoint regression analysis indicated a downward trend in global deaths and DALYs burden, and the ARIMA model predicted that the burden of related diseases will continue to decline through 2041 (ASMR_bladder cancer_=0.44; ASDR_bladder cancer_=8.56; ASMR_kidney cancer_=0.13; ASDR_kidney cancer_=2.82; ASMR_PCa_=0.28; ASDR_PCa_=4.28).

**CONCLUSIONS:**

Smoking has imposed a substantial disease burden on urological cancers over the past three decades. While overall ASDR and ASMR are declining, the disease burden remains high among men, especially those in high-SDI areas. This emphasizes the need for increased tobacco control for these populations or regions.

## INTRODUCTION

Bladder, kidney, and prostate (PCa) cancers are the three main urological cancers and represent significant causes of morbidity and mortality. According to GLOBOCAN, the number of new cases of bladder and kidney cancers rose in rank among all cancers in 2022 compared to 2020 (bladder cancer from 12th to 9th, kidney cancer from 16th to 14th). In addition, the number of deaths from bladder cancer increased from 212536 to 220349, but there was a small decrease in the number of deaths from kidney cancer^1,2^. Among men, PCa was diagnosed as the most common cancer in 118 countries. In terms of cancer deaths, PCa leads in 52 countries, second only to lung cancer^2^. At the same time, as expensive targeted therapies become the standard of care, lost earnings from early mortality and absence from work, as well as possible over-screening and over-treatment, create a heavy financial burden^3,4^.

Smoking is a significant risk factor for bladder cancer, responsible for approximately 50% of cases and increasing the risk by two to three times compared to non-smokers, with a dose-dependent relationship between smoking intensity and tumor aggressiveness^5^. While bladder cancer risk gradually decreases following smoking cessation, former smokers still exhibit a higher risk than never smokers, even after 30 years of abstinence^6^. Smoking also shows moderate to strong positive associations with kidney cancer incidence and mortality, demonstrating a similar dose-dependent pattern where risk escalates dramatically among those smoking ≥30 cigarettes daily^7^. Smoking cessation consistently reduces mortality risk and disease progression compared to continued smoking, with these benefits applying equally to both sexes^8^. The relationship between smoking and PCa remains less clearly established, though pooled analyses indicate smoking is associated with a modestly elevated risk of PCa-specific mortality [hazard ratio (HR)=1.10], which becomes substantially higher when combined with obesity (HR=1.49). However, whether smoking cessation after PCa diagnosis improves prognosis remains controversial, requiring further investigation^9^. Therefore, addressing health issues associated with urological cancers attributable to smoking is complex and challenging. Studying global and regional trends in mortality and disability-adjusted life years (DALYs) of urological cancers attributable to smoking is important to fully understand regional differences in the prevalence of the disease, conduct an informed resource allocation, and develop or strengthen national health policies. In addition, as a modifiable risk factor, attention to smoking-attributable mortality and DALYs of urological cancers can not only further emphasize the need to quit smoking before and after diagnosis, but also provide a basis for exploring the relationship between smoking and postoperative complications, infections and perioperative mortality^10^.

Despite the development of various measures to reduce the mortality of diseases, smoking-attributable urological cancers remain a significant global health challenge. While previous studies have reported the burden of these cancers using data from 2019 or earlier, real-time and accurate epidemiological data are crucial for informing prevention strategies and healthcare resource allocation. Although some studies have used meta-analyses to explore the characteristics of urological cancers attributable to smoking, these studies are often limited by small sample sizes that do not reflect global patterns of disease burden^11^. To address these gaps, we utilized the latest Global Burden of Disease (GBD) 2021 data, covering 204 countries and territories, and employed advanced analytical methods including Joinpoint regression and decomposition analysis. This comprehensive approach not only examines age-sex-time trends in diseases and associations with the sociodemographic index (SDI) but also projects future disease burdens, thereby providing a robust scientific foundation for public health interventions targeting smoking-attributable urological cancers.

## METHODS

### GBD data retrieval

Data for this study were obtained from the GBD 2021 which was conducted by the Institute for Health Metrics and Evaluation (IHME) to provide a comprehensive assessment of three urological cancers attributable to smoking burden over a 32-year period (1990–2021). For data over a 32-year period, our study focused on two time points, 1990 and 2021, as the basis for the analysis. The GBD 2021 calculated various epidemiological metrics by analyzing health-related information gathered from multiple sources, including population censuses, vital statistics, demographic monitoring systems, government health reports, clinical and non-clinical databases, population health studies, peer-reviewed research articles, and other relevant data repositories. The GBD 2021 provides a comprehensive evaluation of health impacts linked to 369 medical conditions, injuries, and disabilities, along with 88 contributing risk elements, covering 204 global regions^12^. This analysis employed up-to-date population health information and improved standardized analytical approaches. In the GBD 2021 classification system, PCa is categorized at the third level and is defined as a malignant neoplasm of the prostate; the International Classification of Diseases (ICD)-10 code is C61. Kidney cancer is defined as a malignant neoplasm of the kidneys; the ICD-10 codes are C64-C65 (level 3). Bladder cancer is defined as a malignant neoplasm of the bladder with the ICD-10 code of C67 (level 3). In addition, tobacco was the level 2 risk factor including tobacco smoking, chewing tobacco use, and secondhand smoke exposure. And in our study, smoking refers to tobacco smoking, not chewing tobacco and secondhand smoke (passive smoking). This study analyzed the global burden of smoking-induced PCa, kidney cancer, and bladder cancer using mortality, age-standardized mortality rates (ASMR), DALYs (incorporating both years lived with disability and years of life lost), and age-standardized disability-adjusted life year rates (ASDR) data using the GBD 2021 Results Tool^13^. The population attributable fraction (PAF) was used to quantify the proportion of urological cancer cases attributable to smoking exposure. PAF represents the reduction in disease burden that would occur if the exposure to smoking were eliminated, assuming other risk factors remain unchanged. This metric essentially measures the fraction of total urological cancer DALYs or mortality that could be prevented by removing smoking as a risk factor. All the above indicators were from GBD 2021 Results Tool. The dataset was systematically stratified across temporal, demographic, and biological dimensions, with comprehensive categorization by calendar year, sex-specific distributions, and quinquennial age cohorts spanning from 30 years to ≥95 years. All rates are expressed per 100000 persons. The 95% uncertainty intervals (UIs) are expressed as the 2.5th and 97.5th values of the ordered 1000 estimates.

The SDI serves as a composite measure of the regional development status. This metric, scaled between 0 and 1, classifies areas into five developmental tiers: low SDI (<0.46); low-middle SDI (0.46–0.60); middle SDI (0.61–0.69); high-middle SDI (0.70–0.81); and high SDI (>0.81)^14^.

Publicly-accessible datasets containing no identifiable personal data were used in this study; therefore, the study was exempt from ethical review. This study adhered to the STROBE protocols for cross-sectional research methodologies (Supplementary file).

### Statistical analysis

The Joinpoint regression model (version 5.1.0.0) was used to analyze the temporal patterns of disease burden using a segmented approach on a logarithmic scale to identify statistically significant trend transitions^15^. This analytical framework allows the calculation of the annual percentage change (APC) and average annual percentage change (AAPC), along with their corresponding 95% confidence intervals (CIs), through geometrically weighted averaging of annual variation rates. The structured methodology of the model facilitates comprehensive trend characterization across multiple temporal segments, allowing for the quantification of disease prevalence and burden trajectories. Furthermore, this approach employs segmented log-linear regression to identify trend inflection points. Using a grid search method (GSM), all possible joinpoints are assessed, with the optimal point chosen according to the smallest mean squared error (MSE). The final number of joinpoints is then determined through a Monte Carlo permutation test, allowing for zero to five joinpoints while ensuring model simplicity. In addition, the estimated annual percentage change (EAPC) is also an important indicator to assess the trends of changes. EAPC is a statistical measure used to quantify the average annual rate of change in a specific metric over time. It represents the percentage change in the metric per year. Statistically significant trends were determined by interpreting the CIs. Positive trends were identified when both APC, AAPC, and EAPC estimates and their CI lower bounds exceeded zero; negative trends were identified when the upper bounds fell below zero. When the CIs encompassed zero, stable patterns were identified. This approach provides a robust assessment of global, regional, and national disease patterns from 1990 to 2021. In order to comprehensively evaluate the stability of the global AAPC estimation results, two sensitivity analyses methods were used. First, to test the sensitivity of trend estimation to the choice of time span, four different time windows of 10, 15, 20, and 25 years were set, and the AAPC and their CIs within each window were calculated. The AAPC was calculated by the annualized rate of change between the start year and the end year of the window. By comparing the AAPC results in different time windows, the consistency and robustness of trend estimation in different observation periods were further verified. Second, a sensitivity analysis was performed by replacing the original estimate (val) with the corresponding lower and upper bounds (lower, upper) provided in the dataset. Specifically, for each year, in addition to the original values, we recalculated the AAPC using the lower and upper bounds of the estimated values. This approach simulates ‘worst case’ and ‘best case’ scenarios, respectively, reflecting the underlying uncertainty inherent in the input data. The differences in AAPC between the three conditions were then compared.

The Das Gupta decomposition method was used to quantitatively assess the drivers of changes in disease burden from 1990 to 2021 by analyzing the independent contributions of population aging, demographic expansion, and epidemiological transitions. This analytical approach, distinct from conventional linear regression methods that focus on variable relationships, allows for a detailed examination of how these three fundamental factors influence temporal patterns in DALYs and deaths due to PCa attributable to smoking, kidney cancer, and bladder cancer at the global, regional, and national levels. By dissecting the composite changes in disease burden into their constituent demographic and epidemiological components, this study provides critical insights into the specific mechanisms driving the observed trends, offering a more nuanced understanding than traditional analytical methods^16^.

An autoregressive integrated moving average (ARIMA) model was used to project the ASDR and ASMR of cancers attributable to smoking (PCa, kidney cancer, and bladder cancer) from 2022 to 2041. In this model framework, the parameters p, d, and q represent the autoregressive order, differencing degree, and moving average order, respectively. Following the logarithmic transformation and differential processing of the time-series data, stationarity was confirmed using the Augmented Dickey-Fuller (ADF) test. Model parameters were identified through autocorrelation (ACF) and partial autocorrelation (PACF) analyses, with optimal model selection based on the Akaike (AIC) and Bayesian (BIC) information criteria. The Ljung-Box Q test used to verify that the model residuals maintained an independent normal distribution, ensuring a robust prediction of smoking-attributable cancer burden trends across the specified timeframe^17^. All data analyses and visualizations were performed using R software (Version 4.4.2), and statistical significance was set at a two-sided p<0.05.

## RESULTS

### Bladder cancer attributable to smoking

**Figure 1:**
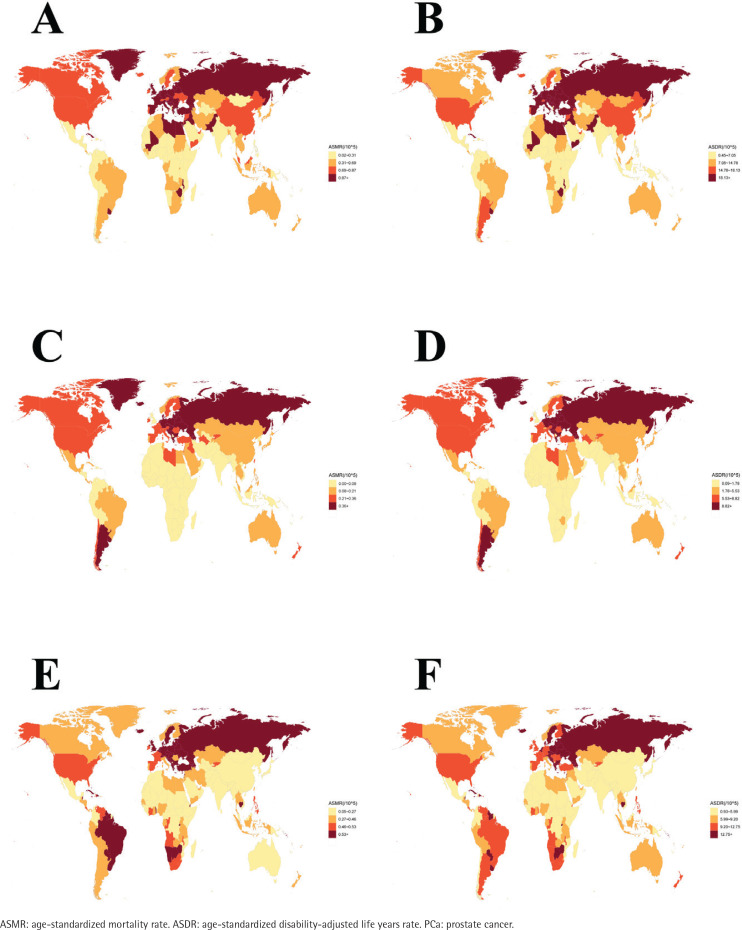
Global distribution of disease burden of urological cancers attributable to smoking in 2021: A) ASMR for bladder cancer; B) ASDR for bladder cancer; C) ASMR for kidney cancer; D) ASDR for kidney cancer; E) ASMR for PCa; F) ASDR for PCa

The global number of bladder cancer deaths caused by smoking increased from 41114 (95% UI: 35100–46965) in 1990 to 58767 (95% UI: 49381–70892) in 2021, a 43% rise, while DALYs increased from 945486 (95% UI: 805856–1076451) to 1238303 (95% UI: 1044303–1478221), an increase of 31%. However, the age-standardized rates (ASR) declined; ASMR fell from 1.13 (95% UI: 0.96–1.29) to 0.70 (95% UI: 0.59–0.84), with AAPC of -1.53 (95% CI: -1.57 – -1.50) and EAPC of -1.71 (95% CI: -1.77 – -1.65), and ASDR dropped from 24.2 (95% UI: 20.63–27.6) to 14.33 (95% UI: 12.09–17.14), with AAPC of -1.68 (95% CI: -1.71 – -1.64) and EAPC of -1.86 (95% CI: -1.92 – -1.8) (Table 1; and Supplementary file: Table 1, Figures 1A and 1B). Furthermore, the global PAF for age-standardized mortality (ASM) fell from 32.19% (95% UI: 28.2–36.25) in 1990 to 26.02% (95% UI: 22.36–29.97) in 2021, and PAF for age-standardized DALY (ASD) fell from 34.05% (95% UI: 29.96–38.08) to 27.78% (95% UI: 24.06–31.56) (Supplementary file Table 2).

**Table 1:** The global burden of bladder cancer attributable to smoking in terms of deaths and disability-adjusted life years (DALYs) from 1990 to 2021, based on Global Burden of Disease (GBD) 2021 data

	*1990*				*2021*				*AAPC*		*EAPC*	
	*Deaths* *(95% UI)*	*ASMR* *(95% UI)*	*DALYs* *(95% UI)*	*ASDR* *(95% UI)*	*Deaths* *(95% UI)*	*ASMR* *(95% UI)*	*DALYs* *(95% UI)*	*ASDR* *(95% UI)*	*ASMR* *(95% CI)*	*ASDR* *(95% CI)*	*ASMR* *(95% CI)*	*ASDR* *(95% CI)*
**Global**	41114 (35100–46965)	1.13 (0.96–1.29)	945486 (805856–1076451)	24.2 (20.63–27.6)	58767 (49381–70892)	0.7 (0.59–0.84)	1238303 (1044303– 1478221)	14.33 (12.09–17.14)	-1.53 (-1.57 – -1.50)	-1.68 (-1.71 – -1.64)	-1.71 (-1.77 – -1.65)	-1.86 (-1.92 – -1.8)
**Sex**												
Male	36423 (30704–41445)	2.37 (2–2.72)	847041 (716268–969269)	48.58 (40.94–55.58)	53090 (44382–64164)	1.45 (1.21–1.75)	1126416 (945018–1348081)	28.52 (23.9–34.2)	-1.59 (-1.62 – -1.56)	-1.71 (-1.74 – -1.67)	-1.76 (-1.82 – -1.71)	-1.9 (-1.96 – -1.84)
Female	4691 (3913–5539)	0.24 (0.19–0.28)	98445 (83645–114594)	4.71 (3.99–5.5)	5677 (4571–6959)	0.12 (0.1–0.15)	111887 (92227–132824)	2.41 (1.99–2.85)	-2.12 (-2.15 – -2.09)	-2.15 (-2.20 – -2.10)	-2.26 (-2.35 – -2.17)	-2.22 (-2.29 – -2.14)
**SDI region**												
Low	637 (472–805)	0.35 (0.26–0.45)	15087 (11151–19107)	7.18 (5.32–9.05)	1127 (911–1421)	0.27 (0.22–0.35)	26248 (21113–32827)	5.53 (4.47–6.94)	-0.83 (-0.88 – -0.77)	-0.84 (-0.89 – -0.79)	-1.04 (-1.15 – -0.94)	-1.09 (-1.19 – -0.99)
Low-middle	2903 (2259–3532)	0.56 (0.44–0.69)	71000 (53668–85594)	12.01 (9.22–14.49)	5103 (4109–7285)	0.4 (0.32–0.57)	116525 (94039–165601)	8.33 (6.73–11.87)	-1.09 (-1.14 – -1.03)	-1.17 (-1.24 – -1.11)	-1.36 (-1.47 – -1.25)	-1.5 (-1.62 – -1.37)
Middle	6774 (5201–8129)	0.77 (0.6–0.92)	163542 (123787–195645)	16.3 (12.46–19.54)	13623 (10820–17705)	0.55 (0.44–0.71)	295302 (233937–385222)	11.12 (8.83–14.45)	-1.07 (-1.13 – -1.01)	-1.23 (-1.28 – -1.17)	-1.31 (-1.42 – -1.2)	-1.44 (-1.55 – -1.34)
High-middle	14202 (11892–16354)	1.5 (1.25–1.73)	337237 (284763–385814)	33.61 (28.29–38.56)	19650 (16416–23607)	0.99 (0.82–1.19)	423434 (356414–505774)	21.06 (17.71–25.17)	-1.34 (-1.40 – -1.28)	-1.50 (-1.56 – -1.43)	-1.55 (-1.66 – -1.43)	-1.73 (-1.84 – -1.61)
High	16540 (14300–18853)	1.46 (1.26–1.66)	357231 (314473–404336)	32.04 (28.21–36.23)	19185 (15485–23062)	0.84 (0.69–1)	375066 (313626–439688)	17.81 (15.02–20.78)	-1.77 (-1.79 – -1.75)	-1.88 (-1.89 – -1.86)	-1.85 (-1.89 – -1.82)	-1.94 (-1.96 – -1.91)
**GBD region**												
Andean Latin America	37 (29–47)	0.21 (0.16–0.26)	782 (620–990)	4.03 (3.2–5.1)	88 (64–119)	0.16 (0.11–0.21)	1746 (1275–2360)	3.01 (2.2–4.09)	-0.89 (-0.99 – -0.79)	-0.93 (-1.03 – -0.84)	-0.91 (-1.1 – -0.73)	-0.98 (-1.16 – -0.8)
Australasia	241 (201–286)	1 (0.83–1.19)	5381 (4541–6311)	22.46 (18.98–26.31)	235 (178–305)	0.41 (0.31–0.52)	4531 (3608–5735)	8.45 (6.8–10.61)	-2.85 (-2.90 – -2.79)	-3.10 (-3.16 – -3.05)	-2.96 (-3.06 – -2.86)	-3.21 (-3.32 – -3.1)
Caribbean	182 (153–214)	0.73 (0.62–0.87)	3871 (3320–4506)	22.46 (18.98–26.31)	318 (253–391)	0.59 (0.47–0.72)	6682 (5387–8119)	8.45 (6.8–10.61)	-0.72 (-0.77 – -0.66)	-0.64 (-0.70 – -0.59)	-0.56 (-0.67 – -0.46)	-0.48 (-0.59 – -0.37)
Central Asia	313 (264–366)	0.68 (0.57–0.79)	8360 (7077–9794)	17.25 (14.64–20.25)	481 (405–570)	0.65 (0.55–0.77)	11977 (10048–14054)	14.57 (12.24–17.19)	-0.15 (-0.25 – -0.05)	-0.54 (-0.66 – -0.43)	-0.15 (-0.34–0.04)	-0.67 (-0.89 – -0.44)
Central Europe	2415 (2114–2721)	1.6 (1.4–1.81)	59561 (52685–66940)	38.61 (34.14–43.39)	3488 (2950–4118)	1.5 (1.27–1.77)	77675 (66589–91156)	35.09 (30.16–41.06)	-0.20 (-0.26 – -0.15)	-0.31 (-0.37 – -0.25)	-0.25 (-0.36 – -0.15)	-0.35 (-0.47 – -0.24)
Central Latin America	266 (227–308)	0.37 (0.31–0.43)	5807 (5017–6680)	7.34 (6.32–8.46)	425 (339–519)	0.18 (0.14–0.22)	8906 (7213–10840)	3.6 (2.9–4.37)	-2.33 (-2.38 – -2.28)	-2.28 (-2.33 – -2.22)	-2.55 (-2.65 – -2.45)	-2.52 (-2.62 – -2.41)
Central Sub-Saharan Africa	48 (36–63)	0.26 (0.19–0.33)	1262 (915–1668)	5.69 (4.21–7.46)	100 (72–135)	0.22 (0.16–0.29)	2666 (1933–3619)	4.86 (3.52–6.58)	-0.55 (-0.73 – -0.38)	-0.51 (-0.68 – -0.34)	-0.46 (-0.8 – -0.13)	-0.41 (-0.73 – -0.09)
East Asia	8667 (5901–10777)	1.22 (0.84–1.5)	209257 (138513–260162)	25.1 (16.93–31.29)	17715 (13442–24011)	0.86 (0.66–1.16)	371442 (278522–503619)	17.03 (12.86–23.12)	-1.12 (-1.18 – -1.05)	-1.24 (-1.31 – -1.18)	-1.35 (-1.47 – -1.23)	-1.47 (-1.6 – -1.35)
Eastern Europe	3079 (2670–3477)	1.08 (0.94–1.22)	78417 (67859–88829)	27.06 (23.48–30.57)	3267 (2706–3800)	0.9 (0.75–1.05)	78927 (65921–92032)	22.12 (18.51–25.79)	-0.58 (-0.81– -0.36)	-0.65 (-0.87 – -0.42)	-1.03 (-1.45 – -0.6)	-1.15 (-1.58 – -0.73)
Eastern Sub-Saharan Africa	183 (136–239)	0.31 (0.23–0.4)	4328 (3220–5674)	6.28 (4.64–8.22)	312 (241–415)	0.23 (0.18–0.3)	7380 (5571–9987)	4.69 (3.61–6.27)	-0.97 (-1.01 – -0.93)	-0.93 (-0.97 – -0.90)	-1.18 (-1.26 – -1.1)	-1.15 (-1.22 – -1.07)
High-income Asia Pacific	1713 (1493–1935)	0.9 (0.79–1.02)	36863 (32662–41261)	18.42 (16.34–20.64)	3038 (2407–3679)	0.53 (0.43–0.63)	51134 (42105–60315)	10.56 (8.79–12.27)	-1.69 (-1.73 – -1.64)	-1.78 (-1.82 – -1.74)	-1.84 (-1.92 – -1.75)	-1.89 (-1.96 – -1.81)
High-income North America	4191 (3586–4908)	1.15 (0.99–1.35)	95155 (81407–109862)	27.25 (23.48–31.37)	5554 (4464–6911)	0.8 (0.64–0.99)	118017 (96876–142893)	17.77 (14.64–21.36)	-1.18 (-1.23 – -1.12)	-1.37 (-1.44 – -1.29)	-1.15 (-1.26 – -1.05)	-1.36 (-1.5 – -1.22)
North Africa and Middle East	1961 (1427–2442)	1.31 (0.97–1.62)	51148 (36003–63842)	30.06 (21.67–37.5)	3820 (3032–4991)	0.97 (0.77–1.26)	92413 (73519–121067)	20.98 (16.63–27.44)	-0.46 (-0.53 – -0.40)	-0.57 (-0.64 – -0.49)	-1.18 (-1.26 – -1.09)	-1.44 (-1.54 – -1.34)
Oceania	6 (4–9)	0.24 (0.15–0.33)	177 (111–245)	5.73 (3.63–7.88)	15 (9–20)	0.21 (0.13–0.29)	423 (260–601)	5.19 (3.22–7.28)	-0.43 (-0.45 – -0.41)	-0.32 (-0.33 – -0.30)	-0.41 (-0.45 – -0.38)	-0.29 (-0.33 – -0.26)
South Asia	2159 (1600–2719)	0.48 (0.35–0.6)	49788 (37091–62611)	9.48 (7.02–11.9)	4190 (3268–5625)	0.33 (0.25–0.44)	90950 (70812–121087)	6.43 (4.99–8.6)	-0.60 (-0.67 – -0.54)	-0.61 (-0.67 – -0.56)	-1.54 (-1.62 – -1.45)	-1.56 (-1.64 – -1.48)
South-East Asia	1006 (789–1219)	0.48 (0.37–0.58)	23546 (18582–28689)	9.77 (7.69–11.87)	2276 (1834–3010)	0.4 (0.32–0.54)	51742 (41719–68489)	8.15 (6.56–10.73)	-0.54 (-0.58 – -0.50)	-0.58 (-0.62 – -0.55)	-0.81 (-0.88 – -0.74)	-0.82 (-0.89 – -0.76)
Southern Latin America	527 (433–622)	1.13 (0.93–1.34)	12781 (10748–14780)	27.07 (22.72–31.3)	518 (415–631)	0.58 (0.47–0.71)	11868 (9712–14206)	13.69 (11.24–16.33)	-2.12 (-2.17 – -2.06)	-2.18 (-2.23 – -2.12)	-1.98 (-2.09 – -1.88)	-2.09 (-2.2 – -1.98)
Southern SubSaharan Africa	162 (121–211)	0.67 (0.5–0.88)	3862 (2894–5008)	14.47 (10.83–18.88)	246 (201–299)	0.47 (0.37–0.56)	6229 (5073–7502)	10.6 (8.62–12.79)	-1.18 (-1.30 – -1.06)	-1.00 (-1.11 – -0.89)	-1.35 (-1.58 – -1.12)	-1.14 (-1.35 – -0.94)
Tropical Latin America	764 (663–871)	0.94 (0.81–1.08)	17678 (15467–20053)	19.87 (17.3–22.64)	1258 (1020–1549)	0.5 (0.41–0.62)	26514 (21772–32137)	10.32 (8.46–12.53)	-2.01 (-2.05 – -1.97)	-2.09 (-2.14 – -2.04)	-2.1 (-2.18 – -2.02)	-2.26 (-2.35 – -2.17)
Western Europe	13085 (11286–14877)	2.16 (1.86–2.45)	274886 (239707–309071)	47.17 (41.3–52.82)	11200 (9033–13504)	1.08 (0.88–1.28)	211726 (175874–251353)	22.82 (19.09–26.8)	-2.21 (-2.24 – -2.19)	-2.32 (-2.33 – -2.30)	-2.27 (-2.31 – -2.23)	-2.34 (-2.37 – -2.31)
Western Sub-Saharan Africa	109 (84–135)	0.15 (0.11–0.18)	2578 (1965–3231)	3.03 (2.34–3.79)	223 (170–292)	0.14 (0.11–0.18)	5355 (4099–7041)	2.84 (2.17–3.72)	-0.16 (-0.19 – -0.13)	-0.21 (-0.25 – -0.17)	-0.18 (-0.24 – -0.12)	-0.27 (-0.34 – -0.19)

DALYs: disability-adjusted life years. ASMR: age-standardized mortality rates. ASDR: age-standardized disability-adjusted life years rates. AAPC: average annual percentage change. EAPC: estimated annual percentage change. SDI: sociodemographic index. UI: uncertainty interval. CI: confidence interval.

Gender disparities were evident in 2021, with male deaths peaking at 9286 (aged 70–74 years) and female deaths at 862 (aged 75–79 years). Males had consistently higher mortality rates across all age groups. The DALYs for both sexes peaked at 70–74 years of age (men, 199763; women, 18175) (Supplementary file Figures 1A–1F). Joinpoint regression showed declining ASMR trends, with the steepest declines for men between 2003 and 2007 (APC= -2.60; 95% CI: -1.57 – -2.94; p<0.001), for women between 2017 and 2021 (APC= -2.78; 95% CI: -2.42 – -3.34; p<0.001), and for both sexes between 2001 and 2007 (APC: -2.30; 95% CI: -1.97 – -2.64; p<0.001) (Supplementary file Figure 2A). Similar decreases were observed in the ASDR (Supplementary file Figure 2B).

Regionally, Central Europe had the highest ASMR (1.5; 95% UI: 1.27–1.77) and ASDR (35.09; 95% UI: 30.16–41.06) in 2021, while Western Sub-Saharan Africa had the lowest (ASMR=0.14; 95% UI: 0.11–0.18; ASDR=2.84; 95% UI: 2.17–3.72). Nationally, Lebanon recorded the highest rates (ASMR=2.94; 95% UI: 2.12–4.01; ASDR=58.87; 95% UI: 43.13–78.57), and Nigeria the lowest (ASMR=0.02; 95% UI: 0.01–0.04; ASDR=0.45; 95% UI: 0.3–0.73) (Supplementary file Table 2). Notably, the highest PAFs for both ASM and ASD were observed in the high-middle SDI region, with 30.33% (95% UI: 26.16–34.67) and 32.97% (95% UI: 28.73–37.07). And the lowest PAFs for both ASM and ASD were observed in the low SDI region, with 12.13% (95% UI: 10.22–14.01) and 12.03% (95% UI: 10.21–13.92) (Supplementary file Table 2).

Decomposition analysis attributed mortality changes to aging (52.92%), population growth (181.23%), and epidemiological changes (-134.15%). The DALYs changes were driven by aging (54.11%), population growth (244.7%), and epidemiological changes (-198.8%) (Supplementary file: Table 3, Figures 3A and 3B). Positive correlations were found between the SDI and ASMR (region: R=0.546; p<0.001; country: R=0.574; p<0.001) and ASDR (region: R=0.554, p<0.001; country: R=0.580, p<0.001) across 204 countries and regions (Supplementary file Figures 4A–4D). At the regional level, ASMR and ASDR initially decline, begin to rise at an SDI of 0.3, begin to decline slightly as they rise to 0.5, and then continue to rise from 0.6 until they peak at about 0.77 and then begin to decline. From 1990 to 2021, ASMR and ASDR decreased significantly in Western Europe, while Western Sub-Saharan Africa and Andean Latin America remained relatively stable. At the national level, ASMR and ASDR increased slowly at the beginning, increased rapidly when SDI was about 0.61, peaked at about 0.76, and then showed small fluctuations. Lebanon, Armenia, and Greece had a significant disease burden. In addition, ASMR and ASDR were highest in the high-middle SDI regions but declined the fastest in the high SDI regions (Table 1).

Projections from the ARIMA model predict continued declines in the global ASMR and ASDR over the next two decades. By 2041, ASMR is expected to drop to 0.44 (95% CI: 0.34–0.55), and ASDR to 8.56 (95% CI: 6.12–11.01) (Supplementary file: Table 4, Figures 5A and 5B).

Sensitivity analysis showed that the global AAPC calculated under different time windows showed a decreasing trend in the same direction, despite minor differences in the estimates. Estimates under longer time windows are smoother, indicating that there is some sensitivity of AAPC to time windows, but the long-term trend is stable, supporting the robustness of the results (Supplementary file Table 5). Furthermore, by comparing AAPC_original val_, AAPC_upper bound val_, and AAPC_lower bound val_ of global ASMR, with -1.53 (95% CI: -1.57 – -1.50), -1.37 (95% CI: -1.40 – -1.34), and -1.58 (95% CI: -1.62 – -1.55); and AAPC_original val_, AAPC_upper bound val_, and AAPC_lower bound val_ of global ASDR, with -1.68 (95% CI: -1.71 – -1.64), -1.53 (95% CI: -1.56 – -1.49), and -1.71 (95% CI: -1.75 – -1.67), it was found that the observed downward trend remained statistically consistent and small, regardless of the range of variation in the input estimates, which further demonstrates the stability of the results.

### Kidney cancer attributable to smoking

The global burden of kidney cancer deaths due to smoking increased from 9673 (95% UI: 6072–13617) in 1990 to 16216 (95% UI: 9663–23217) in 2021, a 67% increase. DALYs increased from 251336 (95% UI: 159401–348868) to 382927 (95% UI: 233635–536755), an increase of 52%. However, the ASR declined: ASMR fell from 0.25 (95% UI: 0.16–0.35) to 0.19 (95% UI: 0.11–0.27), with AAPC of -0.89 (95% CI: -0.95 – -0.84) and EAPC of -0.93 (95% CI: -1.04 – -0.83); and ASDR dropped from 6.17 (95% UI: 3.9–8.61) to 4.37 (95% UI: 2.66–6.14), with AAPC of -1.11 (95% CI: -1.17 – -1.04) and EAPC of -1.15 (95% CI: -1.27 – -1.03) (Table 2; and Supplementary file: Table 6, Figures 1C and 1D). Furthermore, the global PAF for ASM fell from 12.5% (95% UI: 7.81–17.55) in 1990 to 9.87% (95% UI: 5.92–14.1) in 2021. And PAF for ASD fell from 11.64% (95% UI: 7.38–16.2) to 9.23% (95% UI: 5.72–13.01) (Supplementary file Table 7).

**Table 2:** The global burden of kidney cancer attributable to smoking in terms of deaths and disability-adjusted life years (DALYs) from 1990 to 2021, based on Global Burden of Disease (GBD) 2021 data

	*1990*				*2021*				*AAPC*		*EAPC*	
	*Deaths* *(95% UI)*	*ASMR* *(95% UI)*	*DALYs* *(95% UI)*	*ASDR* *(95% UI)*	*Deaths* *(95% UI)*	*ASMR* *(95% UI)*	*DALYs* *(95% UI)*	*ASDR* *(95% UI)*	*ASMR* *(95% CI)*	*ASDR* *(95% CI)*	*ASMR* *(95% CI)*	*ASDR* *(95% CI)*
**Global**	9673 (6072–13617)	0.25 (0.16–0.35)	251336 (159401–348868)	6.17 (3.9–8.61)	16216 (9663–23217)	0.19 (0.11–0.27)	382927 (233635–536755)	4.37 (2.66–6.14)	-0.89 (-0.95 – -0.84)	-1.11 (-1.17 – -1.04)	-0.93 (-1.04 – -0.83)	-1.15 (-1.27 – -1.03)
**Sex**												
Male	8058 (5114–11295)	0.46 (0.29–0.66)	212419 (136033–293300)	11.18 (7.11–15.6)	14118 (8504–20025)	0.36 (0.22–0.52)	338472 (207556–470872)	8.21 (5.01–11.5)	-0.81 (-0.84 – -0.78)	-1.00 (-1.02 – -0.97)	-0.85 (-0.94 – -0.76)	-1.04 (-1.15 – -0.93)
Female	1615 (975–2309)	0.08 (0.05–0.11)	38917 (23751–54853)	1.82 (1.11–2.57)	2098 (1204–3255)	0.04 (0.03–0.07)	44455 (26496–66466)	0.96 (0.57–1.43)	-1.76 (-1.81 – -1.72)	-2.08 (-2.12 – -2.04)	-1.72 (-1.88 – -1.55)	-2 (-2.17 – -1.83)
**SDI region**												
Low	47 (27–68)	0.02 (0.01–0.03)	1194 (694–1717)	0.53 (0.31–0.77)	101 (56–151)	0.02 (0.01–0.03)	2536 (1407–3762)	0.5 (0.28–0.75)	-0.14 (-0.17 – -0.10)	-0.17 (-0.20 – -0.13)	-0.29 (-0.36 – -0.22)	-0.35 (-0.41 – -0.28)
Low-middle	282 (176–393)	0.05 (0.03–0.07)	7261 (4549–10079)	1.17 (0.73–1.63)	735 (455–1050)	0.05 (0.03–0.08)	18224 (11317–25873)	1.25 (0.77–1.77)	0.23 (0.21–0.26)	0.20 (0.18–0.22)	0.21 (0.17–0.26)	0.18 (0.14–0.22)
Middle	942 (615–1283)	0.1 (0.06–0.13)	25284 (16536–34605)	2.35 (1.53–3.21)	2802 (1718–3871)	0.11 (0.06–0.15)	70248 (43364–97386)	2.51 (1.55–3.48)	0.28 (0.24–0.31)	0.22 (0.17–0.26)	0.36 (0.29–0.43)	0.33 (0.25–0.42)
High-middle	2963 (1918–4089)	0.29 (0.19–0.41)	80770 (52628–110173)	7.78 (5.06–10.64)	5509 (3390–7688)	0.27 (0.17–0.38)	138425 (86981–191405)	6.88 (4.32–9.5)	-0.24 (-0.33 – -0.15)	-0.40 (-0.49 – -0.31)	-0.32 (-0.49 – -0.15)	-0.5 (-0.67 – -0.32)
High	5423 (3350–7710)	0.49 (0.3–0.69)	136388 (85078–192526)	12.65 (7.91–17.8)	7046 (4030–10669)	0.33 (0.19–0.49)	152961 (89126–226095)	7.68 (4.54–11.29)	-1.29 (-1.35 – -1.23)	-1.60 (-1.66 – -1.53)	-1.34 (-1.44 – -1.23)	-1.64 (-1.76 – -1.51)
**GBD region**												
Andean Latin America	9 (5–14)	0.05 (0.03–0.07)	212 (123–319)	1.06 (0.61–1.6)	31 (17–51)	0.05 (0.03–0.09)	666 (371–1058)	1.14 (0.63–1.82)	0.37 (0.30–0.44)	0.24 (0.17–0.30)	0.52 (0.39–0.66)	0.35 (0.23–0.48)
Australasia	89 (53–132)	0.37 (0.22–0.55)	2176 (1319–3190)	9.35 (5.66–13.7)	110 (58–181)	0.2 (0.11–0.33)	2403 (1339–3786)	4.76 (2.69–7.4)	-1.97 (-2.01 – -1.94)	-2.15 (-2.19 – -2.11)	-2.09 (-2.16 – -2.03)	-2.22 (-2.29 – -2.15)
Caribbean	33 (20–50)	0.13 (0.08–0.2)	838 (513–1228)	3.21 (1.96–4.71)	67 (39–101)	0.12 (0.07–0.19)	1598 (931–2361)	2.95 (1.72–4.36)	-0.16 (-0.22 – -0.11)	-0.27 (-0.33 – -0.21)	-0.15 (-0.26 – -0.03)	-0.25 (-0.36 – -0.13)
Central Asia	75 (47–110)	0.15 (0.09–0.22)	2219 (1402–3211)	4.38 (2.76–6.35)	186 (119–262)	0.22 (0.14–0.31)	5299 (3398–7461)	5.84 (3.75–8.25)	1.16 (0.98–1.34)	0.93 (0.75–1.10)	1.35 (1–1.7)	1.03 (0.69–1.38)
Central Europe	777 (500–1087)	0.51 (0.32–0.71)	21030 (13703–28830)	13.59 (8.84–18.64)	1091 (657–1590)	0.49 (0.3–0.71)	26128 (16114–37549)	12.35 (7.66–17.67)	-0.11 (-0.22 – -0.00)	-0.31 (-0.43 – -0.18)	-0.05 (-0.26–0.16)	-0.24 (-0.47–0)
Central Latin America	86 (51–124)	0.11 (0.07–0.16)	2068 (1248–2945)	2.51 (1.51–3.6)	186 (108–278)	0.08 (0.04–0.11)	4249 (2514–6243)	1.69 (1–2.48)	-1.24 (-1.30 – -1.18)	-1.28 (-1.34 – -1.22)	-1.38 (-1.49 – -1.27)	-1.46 (-1.57 – -1.34)
Central Sub-Saharan Africa	3 (2–5)	0.01 (0.01–0.03)	83 (44–148)	0.36 (0.18–0.63)	9 (4–17)	0.02 (0.01–0.03)	256 (115–470)	0.44 (0.19–0.8)	0.58 (0.42–0.75)	0.66 (0.50–0.81)	0.76 (0.43–1.08)	0.83 (0.52–1.13)
East Asia	989 (642–1391)	0.13 (0.08–0.18)	26737 (17317–37593)	2.98 (1.93–4.19)	3614 (2156–5143)	0.17 (0.1–0.23)	90535 (53869–127788)	3.99 (2.37–5.65)	0.89 (0.80–0.97)	0.95 (0.86–1.05)	1.15 (0.99–1.31)	1.27 (1.09–1.46)
Eastern Europe	1109 (732–1540)	0.38 (0.25–0.53)	32358 (21493–44764)	11.15 (7.41–15.47)	1659 (1053–2309)	0.46 (0.29–0.65)	44638 (28604–61835)	12.81 (8.22–17.74)	0.62 (0.45–0.78)	0.45 (0.27–0.62)	0.31 (-0.01–0.63)	0.08 (-0.26–0.41)
Eastern Sub-Saharan Africa	14 (8–20)	0.02 (0.01–0.03)	345 (195–509)	0.47 (0.27–0.69)	33 (16–51)	0.02 (0.01–0.03)	823 (403–1302)	0.49 (0.25–0.77)	0.15 (0.11–0.19)	0.17 (0.13–0.21)	0.09 (0.01–0.16)	0.11 (0.03–0.18)
High-income Asia Pacific	543 (356–742)	0.27 (0.18–0.37)	13732 (9110–18509)	6.59 (4.36–8.9)	980 (574–1443)	0.2 (0.12–0.28)	18364 (11191–26517)	4.26 (2.63–6.08)	-1.01 (-1.09 – -0.92)	-1.40 (-1.50 – -1.30)	-1.2 (-1.36 – -1.04)	-1.55 (-1.74 – -1.36)
High-income North America	1831 (1090–2629)	0.54 (0.32–0.77)	48457 (29458–69128)	14.87 (9.09–21.13)	2365 (1276–3741)	0.35 (0.19–0.55)	55145 (31522–84516)	8.66 (5.04–13.2)	-1.34 (-1.43 – -1.25)	-1.73 (-1.82 – -1.64)	-1.46 (-1.63 – -1.29)	-1.86 (-2.04 – -1.69)
North Africa and Middle East	198 (120–275)	0.12 (0.07–0.17)	5502 (3399–7546)	3.07 (1.87–4.24)	582 (360–849)	0.13 (0.08–0.2)	15605 (9791–22589)	3.25 (2.03–4.76)	0.15 (0.11–0.18)	0.09 (0.06–0.12)	0.25 (0.2–0.29)	0.14 (0.09–0.18)
Oceania	1 (0–1)	0.02 (0.01–0.04)	17 (8–27)	0.56 (0.27–0.9)	1 (1–2)	0.02 (0.01–0.03)	40 (18–68)	0.49 (0.23–0.84)	-0.52 (-0.58 – -0.46)	-0.46 (-0.52 – -0.40)	-0.64 (-0.75 – -0.52)	-0.59 (-0.7 – -0.48)
South Asia	213 (133–298)	0.04 (0.03–0.06)	5365 (3351–7485)	0.94 (0.59–1.32)	528 (329–739)	0.04 (0.02–0.05)	12512 (7705–17580)	0.84 (0.52–1.19)	-0.14 (-0.19– -0.09)	-0.17 (-0.21– -0.13)	-0.51 (-0.58– -0.45)	-0.56 (-0.62– -0.5)
South-East Asia	172 (107–242)	0.07 (0.05–0.1)	4524 (2800–6352)	1.73 (1.07–2.44)	487 (310–685)	0.08 (0.05–0.11)	12609 (7990–17738)	1.83 (1.16–2.57)	0.17 (0.12–0.23)	0.17 (0.12–0.23)	-0.07 (-0.17–0.04)	-0.05 (-0.16–0.05)
Southern Latin America	187 (112–270)	0.4 (0.24–0.58)	5167 (3199–7313)	10.94 (6.78–15.49)	345 (195–525)	0.4 (0.22–0.6)	8732 (5156–12825)	10.25 (6.06–14.98)	-0.01 (-0.12–0.10)	-0.21 (-0.31– -0.11)	0.1 (-0.11–0.32)	-0.12 (-0.32–0.08)
Southern SubSaharan Africa	21 (11–31)	0.08 (0.04–0.12)	539 (298–809)	1.94 (1.06–2.92)	34 (20–47)	0.06 (0.04–0.09)	890 (543–1247)	1.47 (0.9–2.06)	-0.94 (-0.99 – -0.88)	-0.89 (-0.95 – -0.84)	-0.86 (-0.96 – -0.76)	-0.79 (-0.89 – -0.69)
Tropical Latin America	177 (109–252)	0.2 (0.12–0.28)	4712 (2937–6648)	4.95 (3.06–7.02)	445 (248–686)	0.17 (0.1–0.27)	10553 (6022–15842)	4.03 (2.3–6.06)	-0.43 (-0.47 – -0.39)	-0.66 (-0.71 – -0.61)	-0.5 (-0.58 – -0.43)	-0.79 (-0.88 – -0.7)
Western Europe	3140 (1936–4437)	0.54 (0.33–0.76)	75114 (46895–105381)	13.56 (8.5–19.02)	3447 (1972–5219)	0.36 (0.21–0.53)	71438 (41622–104623)	8.25 (4.85–11.9)	-1.32 (-1.36 – -1.29)	-1.59 (-1.64 – -1.54)	-1.26 (-1.32 – -1.19)	-1.51 (-1.59 – -1.42)
Western Sub-Saharan Africa	6 (3–9)	0.01 (0–0.01)	141 (80–215)	0.16 (0.09–0.24)	17 (10–26)	0.01 (0.01–0.01)	444 (256–681)	0.22 (0.12–0.34)	0.96 (0.93–0.99)	1.02 (0.99–1.05)	1.07 (1.01–1.13)	1.1 (1.04–1.17)

DALYs: disability-adjusted life years. ASMR: age-standardized mortality rates. ASDR: age-standardized disability-adjusted life years rates. AAPC: average annual percentage change. EAPC: estimated annual percentage change. SDI: sociodemographic index. UI: uncertainty interval. CI: confidence interval.

In 2021, deaths peaked at age 70–74 years (2370 males, 358 females), and DALYs peaked at age 65–69 years (59799 males, 7895 females) (Supplementary file Figures 1G–1L). Joinpoint analysis showed that ASMR for both sexes and males increased from 1990 to 1994 (both sexes: APC=0.65; 95% CI: 0.39–0.95; p<0.001; males: APC=0.66; 95% CI: 0.39–0.99; p<0.001), then declined from 1995 to 2021. In female patients, ASMR declined fastest between 2016 and 2021 (APC= -3.31; 95% CI: -3.96 – -3.00; p<0.001) (Supplementary file Figure 2C). The ASDR trends mirrored those of ASMR (Figure 3D).

Regionally, Central Europe had the highest ASMR (0.49; 95% UI: 0.3–0.71), and Eastern Europe had the highest ASDR (12.81; 95% UI: 8.22–17.74) in 2021. Western Sub-Saharan Africa had the lowest ASMR (0.01; 95% UI: 0.01–0.01) and ASDR (0.22; 95% UI: 0.12–0.34). Nationally, Uruguay had the highest ASMR (0.71; 95% UI: 0.41–1.06) and ASDR (18.13; 95% UI: 10.82–26.34), while Niger had the lowest ASMR (0; 95% UI: 0–0.01) and ASDR (0.09; 95% UI: 0.04–0.15) (Supplementary file Table 7). The highest PAFs for both ASM and ASD were observed in the high-middle SDI region, with 11.41% (95% UI: 7.14–15.7) and 11.24% (95% UI: 7.19–15.45), respectively. And the lowest PAFs for both ASM and ASD were observed in the low SDI region, with 2.63% (95% UI: 1.63–3.75) and 1.94% (95% UI: 1.2–2.78), respectively (Supplementary file Table 7).

Decomposition analysis attributed the mortality changes to aging (28.86%), population growth (121.16%), and epidemiological changes (-50.02%). The DALYs changes were driven by aging (-9.41%), population growth (96.13%), and epidemiological changes (13.29%) (Supplementary file: Table 3, Figures 3C and 3D). The SDI was positively correlated with ASMR (region: R=0.859, p<0.001; country: R=0.792, p<0.001) and ASDR (region: R=0.848, p<0.001; country: R=0.783, p<0.001) (Supplementary file Figures 4E–4H). At the regional level, ASMR and ASDR initially decreased slightly and then increased from SDI of 0.2 until they peaked at about 0.74 and began to decline. ASMR and ASDR increased rapidly from about 0.6 to 0.74. From 1990 to 2021, high-income North America, Western Europe, and Australasia had the fastest declines in ASMR and ASDR, while North Africa and Middle East, South-East Asia, Andean Latin America, South Asia, Western Sub-Saharan Africa, Oceania, Eastern Sub-Saharan Africa, and Central Sub-Saharan Africa were stable. At the national level, ASMR and ASDR began to rise slowly, then increased rapidly from SDI around 0.62 until peaking at 0.85, and then continued to decline. Uruguay and Greenland have a high disease burden. From 1990 to 2021, ASDR and ASMR declined in the low-middle and middle SDI regions, but increased in others (Table 2). ARIMA predicts that the global ASMR will reach 0.13 (95% CI: 0.05–0.20) and the global ASDR will reach 2.82 (95% CI: 0.22–5.42) in the next 20 years (Supplementary file: Table 4, Figures 5C and 5D).

**Table 3:** The global burden of prostate cancer attributable to smoking in terms of deaths and disability-adjusted life years (DALYs) from 1990 to 2021, based on Global Burden of Disease (GBD) 2021 data

	1990				2021				AAPC		EAPC	
	*Deaths* *(95% UI)*	*ASMR* *(95% UI)*	*DALYs* *(95% UI)*	*ASDR* *(95% UI)*	*Deaths* *(95% UI)*	*ASMR* *(95% UI)*	*DALYs* *(95% UI)*	*ASDR* *(95% UI)*	*ASMR* *(95% CI)*	*ASDR* *(95% CI)*	*ASMR* *(95% CI)*	*ASDR* *(95% CI)*
**Global**	9937 (4652–15975)	0.69 (0.32–1.13)	218511 (102373–342894)	13.28 (6.23–21.18)	12992 (5893–21582)	0.36 (0.16–0.6)	281979 (131119–458053)	7.17 (3.32–11.81)	-2.10 (-2.16 – -2.04)	-1.97 (-2.02 – -1.92)	-2.42 (-2.53 – -2.32)	-2.26 (-2.35 – -2.17)
**SDI region**												
Low	187 (73–315)	0.21 (0.08–0.35)	4342 (1648–7289)	4.13 (1.6–6.94)	371 (157–612)	0.19 (0.08–0.31)	8520 (3523–13974)	3.76 (1.57–6.2)	-0.32 (-0.38 – -0.26)	-0.30 (-0.36 – -0.24)	-0.52 (-0.64 – -0.41)	-0.53 (-0.64 – -0.42)
Low-middle	619 (267–1006)	0.26 (0.11–0.42)	13658 (5838–22091)	4.93 (2.12–8)	1469 (650–2368)	0.26 (0.12–0.42)	31499 (14101–50256)	4.96 (2.21–8.01)	0.04 (0.01–0.08)	0.02 (-0.01–0.05)	-0.04 (-0.12–0.03)	-0.09 (-0.15 – -0.03)
Middle	1323 (594–2079)	0.36 (0.16–0.57)	29963 (13434–46776)	6.8 (3.06–10.72)	2989 (1349–4870)	0.28 (0.12–0.45)	64234 (29527–101335)	5.29 (2.41–8.5)	-0.86 (-0.93 – -0.80)	-0.81 (-0.86 – -0.75)	-1.05 (-1.17 – -0.94)	-1.02 (-1.11 – -0.92)
High-middle	2383 (1121–3751)	0.69 (0.32–1.11)	53622 (25329–83377)	13.43 (6.31–21.03)	3515 (1591–5643)	0.42 (0.19–0.69)	78210 (35829–122601)	8.72 (3.97–13.82)	-1.57 (-1.66 – -1.49)	-1.38 (-1.46 – -1.30)	-1.81 (-1.97 – -1.64)	-1.59 (-1.74 – -1.44)
High SDI	5408 (2518–8773)	1.24 (0.57–2.01)	116572 (54464–188035)	25.07 (11.65–40.52)	4627 (2061–7895)	0.46 (0.21–0.79)	99057 (45349–162756)	10.14 (4.66–16.59)	-3.12 (-3.21 – -3.04)	-2.88 (-2.94 – -2.81)	-3.63 (-3.78 – -3.47)	-3.3 (-3.43 – -3.18)
**GBD region**												
Andean Latin America	38 (16–70)	0.46 (0.19–0.84)	746 (320–1337)	8.32 (3.55–15.06)	95 (40–183)	0.37 (0.15–0.71)	1823 (739–3502)	6.79 (2.76–13.04)	-0.69 (-0.75 – -0.63)	-0.66 (-0.72 – -0.59)	-0.91 (-1.02 – -0.8)	-0.9 (-1.02 – -0.78)
Australasia	108 (49–179)	1.07 (0.48–1.79)	2428 (1134–3982)	22.72 (10.49–37.31)	80 (33–149)	0.3 (0.12–0.56)	1745 (744–3054)	6.82 (2.92–11.9)	-4.01 (-4.21 – -3.82)	-3.81 (-4.06 – -3.56)	-4.53 (-4.89 – -4.16)	-4.26 (-4.72 – -3.81)
Caribbean	227 (97–402)	1.07 (0.49–1.83)	2518 (1155–4204)	20.76 (9.55–34.95)	122 (57–207)	0.93 (0.39–1.65)	4711 (2087–8324)	18.84 (8.34–33.35)	-0.45 (-0.56 – -0.35)	-0.31 (-0.41 – -0.21)	-0.64 (-0.83 – - 0.44)	-0.49 (-0.68 – -0.29)
Central Asia	125 (59–193)	0.37 (0.17–0.59)	1608 (756–2490)	8.56 (4–13.38)	63 (29–98)	0.43 (0.2–0.67)	3070 (1436–4702)	9.06 (4.27–14)	0.44 (0.27–0.61)	0.18 (0.03–0.33)	1.51 (1.18–1.84)	1.14 (0.85–1.43)
Central Europe	644 (297–1041)	0.89 (0.42–1.46)	11795 (5663–18628)	18.54 (8.88–29.53)	524 (251–845)	0.67 (0.31–1.09)	13865 (6446–22402)	14.15 (6.59–22.85)	-0.91 (-1.05 – -0.77)	-0.87 (-1.00 – -0.74)	-1.27 (-1.53 – -1)	-1.19 (-1.43 – - 0.94)
Central Latin America	162 (76–257)	0.48 (0.22–0.77)	3452 (1654–5373)	9.4 (4.49–14.79)	291 (129–497)	0.27 (0.12–0.47)	6351 (2878–10677)	5.7 (2.55–9.63)	-1.78 (-1.93 – -1.62)	-1.60 (-1.75 – -1.46)	-2.26 (-2.55 – -1.96)	-2.12 (-2.4 – -1.84)
Central Sub-Saharan Africa	18 (7–32)	0.23 (0.09–0.4)	446 (163–780)	4.65 (1.74–8.1)	44 (17–76)	0.25 (0.1–0.46)	1087 (413–1901)	5.07 (1.98–8.8)	0.34 (0.20–0.47)	0.28 (0.15–0.41)	0.4 (0.14–0.66)	0.34 (0.08–0.59)
East Asia	2019 (861–3407)	0.25 (0.11–0.41)	17452 (7509–28609)	4.64 (1.99–7.68)	729 (315–1207)	0.23 (0.1–0.38)	42557 (18315–70623)	4.23 (1.81–7.06)	-0.24 (-0.31 – -0.17)	-0.29 (-0.35 – -0.24)	-0.37 (-0.49 – -0.24)	-0.41 (-0.51 – -0.31)
Eastern Europe	531 (253–830)	0.6 (0.29–0.95)	13557 (6467–21176)	13.73 (6.53–21.31)	1109 (525–1787)	0.85 (0.4–1.37)	28022 (13268–44380)	20.19 (9.56–32.26)	1.11 (0.96–1.26)	1.25 (1.10–1.41)	1.26 (0.97–1.54)	1.37 (1.07–1.68)
Eastern Sub-Saharan Africa	84 (30–146)	0.28 (0.1–0.48)	1986 (694–3467)	5.7 (2.02–9.88)	150 (59–252)	0.23 (0.09–0.39)	3629 (1436–6208)	4.88 (1.93–8.25)	-0.61 (-0.69 – -0.53)	-0.50 (-0.58 – -0.42)	-0.86 (-1.01 – -0.71)	-0.76 (-0.91 – -0.61)
High-income Asia Pacific	282 (131–454)	0.38 (0.18–0.63)	5895 (2761–9237)	7.22 (3.36–11.51)	536 (239–919)	0.23 (0.1–0.39)	9667 (4462–15619)	4.38 (2.03–7.03)	-1.67 (-1.84 – -1.50)	-1.60 (-1.76 – -1.44)	-2.26 (-2.57 – -1.94)	-2.09 (-2.39 – -1.79)
High-income North America	1556 (652–2810)	1.45 (0.65–2.37)	50711 (22874–81259)	33.28 (15.01–53.29)	2143 (959–3506)	0.5 (0.21–0.92)	38256 (17307–64627)	12.37 (5.58–20.82)	-3.35 (-3.48 – -3.22)	-3.14 (-3.27 – -3.02)	-3.95 (-4.18 – -3.71)	-3.68 (-3.91 – -3.46)
North Africa and Middle East	273 (119–436)	0.42 (0.18–0.7)	5968 (2567–9595)	7.96 (3.48–12.77)	601 (257–1036)	0.33 (0.14–0.57)	13522 (5673–23089)	6.59 (2.8–11.31)	-0.38 (-0.43 – -0.33)	-0.30 (-0.34 – -0.26)	-0.95 (-1.02 – -0.89)	-0.77 (-0.83 – -0.72)
Oceania	10 (4–18)	0.35 (0.14–0.6)	89 (36–152)	6.75 (2.75–11.69)	4 (2–6)	0.33 (0.13–0.61)	232 (90–428)	6.63 (2.58–12.2)	-0.19 (-0.23 – -0.14)	-0.05 (-0.09 – -0.02)	-0.1 (-0.19 – -0.02)	0.03 (-0.05–0.1)
South Asia	404 (165–682)	0.18 (0.07–0.3)	8927 (3590–14769)	3.38 (1.38–5.68)	911 (410–1578)	0.15 (0.07–0.27)	18728 (8293–32376)	2.84 (1.26–4.93)	-0.21 (-0.29 – -0.12)	-0.27 (-0.36 – -0.19)	-0.8 (-0.92 – -0.69)	-0.94 (-1.05 – -0.83)
South-East Asia	308 (130–509)	0.34 (0.14–0.56)	6868 (2878–11287)	6.5 (2.73–10.75)	828 (350–1384)	0.34 (0.14–0.57)	18635 (7952–30672)	6.61 (2.82–11.03)	-0.01 (-0.05–0.04)	0.06 (0.02–0.10)	-0.03 (-0.12–0.06)	0.03 (-0.04–0.1)
Southern Latin America	124 (57–201)	0.64 (0.29–1.06)	2914 (1344–4671)	14.01 (6.44–22.53)	158 (70–273)	0.42 (0.19–0.73)	3472 (1605–5868)	8.99 (4.13–15.19)	-1.33 (-1.53 – -1.13)	-1.42 (-1.62 – -1.22)	-1.39 (-1.77 – -1.01)	-1.51 (-1.9 – -1.13)
Southern Sub-Saharan Africa	99 (43–173)	1.09 (0.47–1.93)	2161 (953–3670)	20.6 (9–35.58)	128 (55–206)	0.64 (0.27–1.02)	3131 (1362–4997)	13.47 (5.79–21.44)	-1.72 (-1.78 – -1.67)	-1.36 (-1.41 – -1.31)	-1.71 (-1.82 – -1.61)	-1.34 (-1.44 – -1.25)
Tropical Latin America	343 (161–553)	0.96 (0.45–1.58)	7696 (3630–12274)	19.25 (9.09–30.75)	688 (297–1217)	0.66 (0.28–1.17)	14239 (6252–24260)	12.74 (5.58–21.96)	-1.21 (-1.28 – -1.14)	-1.32 (-1.41 – -1.24)	-1.39 (-1.52 – -1.26)	-1.62 (-1.78 – -1.46)
Western Europe	3495 (1652–5575)	1.52 (0.71–2.45)	69438 (33373–110205)	28.83 (13.83–46.12)	2574 (1122–4429)	0.56 (0.25–0.96)	50258 (22956–82734)	11.58 (5.34–18.87)	-3.18 (-3.28 – -3.08)	-2.90 (-2.99 – -2.81)	-3.69 (-3.87 – -3.51)	-3.32 (-3.49 – -3.16)
Western Sub-Saharan Africa	82 (32–146)	0.23 (0.09–0.41)	1858 (716–3318)	4.57 (1.75–8.09)	220 (75–401)	0.3 (0.1–0.55)	4978 (1686–9010)	5.93 (2.02–10.81)	0.77 (0.74–0.80)	0.84 (0.81–0.88)	0.85 (0.8–0.9)	0.91 (0.84–0.98)

DALYs: disability-adjusted life years. ASMR: age-standardized mortality rates. ASDR: age-standardized disability-adjusted life years rates. AAPC: average annual percentage change. EAPC: estimated annual percentage change. SDI: sociodemographic index. UI: uncertainty interval. CI: confidence interval.

Sensitivity analysis showed that the global AAPC calculated under different time windows showed a decreasing trend in the same direction, despite minor differences in the estimates. Estimates under longer time windows are smoother, indicating that there is some sensitivity of AAPC to time windows, but the long-term trend is stable, supporting the robustness of the results (Supplementary file Table 5). Furthermore, by comparing AAPC_original val_, AAPC_upper bound val_, and AAPC_lower bound val_ of global ASMR, with -0.89 (95% CI: -0.95 – -0.84), -0.84 (95% CI: -0.89 – -0.78), and -1.05 (95% CI: -1.11 – -0.99); and AAPC_original val_, AAPC_upper bound val_, and AAPC_lower bound val_ of global ASDR, with -1.11 (95% CI: -1.17 – -1.04), -1.09 (95% CI: -1.15 – -1.02), and -1.23 (95% CI: -1.30 – -1.16), it was found that the observed downward trend remained statistically consistent and small, regardless of the range of variation in the input estimates, which further demonstrates the stability of the results.

### PCa attributable to smoking

The global burden of PCa deaths attributable to smoking increased from 9937 (95% UI: 4652–15975) in 1990 to 12992 (95% UI: 5893–21582) in 2021, a 31% increase. DALYs grew from 218511 (95% UI: 102373–342894) to 281979 (95% UI: 131119–458053), an increase of 29%. However, the ASRs declined: ASMR decreased from 0.69 (95% UI: 0.32–1.13) to 0.36 (95% UI: 0.16–0.60), with AAPC of -2.10 (95% CI: -2.16 – -2.04) and EAPC of -2.42 (95% CI: -2.53 – -2.32), and ASDR dropped from 13.28 (95% UI: 6.23–21.18) to 7.17 (95% UI: 3.32–11.81), with AAPC of -1.97 (95% CI: -2.02 – -1.92) and EAPC of -2.26 (95% CI: -2.35 – -2.17) (Table 3; and Supplementary file: Table 8, Figures 1E and 1F). Moreover, the global PAF for ASM fell from 4.21% (95% UI: 1.98–6.76) in 1990 to 2.82% (95% UI: 1.32–4.68) in 2021. And PAF for ASD fell from 4.82% (95% UI: 2.3–7.63) to 3.29% (95% UI: 1.56–5.33) (Supplementary file Table 9). In 2021, the death and DALY rates rose steadily from the ages of 30 to 94 years before declining (Supplementary file Figures 1M–1R). Joinpoint analysis revealed that ASMR and ASDR declined overall, with the steepest drop in ASMR from 2002 to 2007 (APC= -3.25; p<0.001) and ASDR from 2002 to 2013 (APC= -2.83; p<0.001) (Supplementary file Figures 2E and 2F).

Regionally, the Caribbean had the highest ASMR (0.93; 95% UI: 0.39–1.65), and Eastern Europe had the highest ASDR (20.19; 95% UI: 9.56–32.26) in 2021. South Asia had the lowest ASMR (0.15; 95% UI: 0.07–0.27) and ASDR (2.84; 95% UI: 1.26–4.93). Nationally, Seychelles had the highest ASMR (1.78; 95% UI: 0.75–3.03), Georgia had the highest ASDR (36.72; 95% UI: 16.18–57.91), and Ethiopia had the lowest ASMR (0.05; 95% UI: 0.02–0.09) and ASDR (0.93; 95% UI: 0.32–1.84) (Supplementary file Table 9). The highest PAFs for both ASM and ASD were observed in the high-middle SDI region, with 3.58% (95% UI: 1.69–5.73) and 4.33% (95% UI: 2.09–6.76). And the lowest PAFs for both ASM and ASD were observed in the low SDI region, with 1.12% (95% UI: 0.52–1.84) and 1.28% (95% UI: 0.61– 2.09) (Supplementary file Table 9).

Decomposition analysis attributed mortality changes to aging (116.64%), population growth (286.82%), and epidemiological changes (-303.46%). The DALYs changes were driven by aging (97.18%), population growth (313.54%), and epidemiological changes (-310.72%) (Supplementary file: Table 3, Figures 3E and 3F). The SDI was positively correlated with ASMR (region: R=0.602, p<0.001; country: R=0.417, p<0.001) and ASDR (region: R=0.612, p<0.001; country: R=0.436, p<0.001) (Supplementary file Figures 4I–4L). At the regional level, ASMR and ASDR began to decline, then began to rise at about 0.35 SDI until 0.55, remained stable between 0.55 and 0.65, then continued to rise and peaked at about 0.75 and continued to decline. From 1990 to 2021, high-income North America, Western Europe, and Australasia showed the most significant decreases in ASMR and ASDR. At the national level, ASMR and ASDR initially showed a volatile rise until they peaked at an SDI of about 0.76 and then began to decline. Georgia, Seychelles, and Zimbabwe had a high disease burden, while Oman and Saudi Arabia had a low burden. From 1990 to 2021, the ASDR and ASMR declined in the low-middle SDI regions but increased elsewhere (Table 3). ARIMA predicts that the global ASMR will reach 0.28 (95% CI: 0.06–0.51) and the global ASDR will reach 4.28 (95% CI: 1.94–6.61) in the next 20 years (Supplementary file: Table 4, Figures 5E and 5F).

Sensitivity analysis showed that the global AAPC calculated under different time windows showed a decreasing trend in the same direction, despite minor differences in the estimates. Estimates under longer time windows are smoother, indicating that there is some sensitivity of AAPC to time windows, but the long-term trend is stable, supporting the robustness of the results (Supplementary file Table 5). Furthermore, by comparing AAPC_original val_, AAPC_upper bound val_, and AAPC_lower bound val_ of global ASMR, with -2.10 (95% CI: -2.16 – -2.04), -1.99 (95% CI: -2.05 – -1.93), and -2.16 (95% CI: -2.22 – -2.10); and AAPC_original val_, AAPC_upper bound val_, and AAPC_lower bound val_ of global ASDR, with -1.97 (95% CI: -2.02 – -1.92), -1.87 (95% CI: -1.92 – -1.81), and -2.01 (95% CI: -2.06 – -1.96), it was found that the observed downward trend remained statistically consistent and small, regardless of the range of variation in the input estimates, which further demonstrates the stability of the results.

## DISCUSSION

This study has several key findings. First, the trend analysis indicates that, while the absolute number of deaths and DALYs from urological cancers attributable to smoking has increased globally, ASMR and ASDR have shown a declining trend. Second, men exhibited higher absolute numbers of deaths and DALYs and higher ASRs than women. This suggests that males bear a heavier burden of disease than females. Third, regions or nations with a higher SDI tended to have increased ASMR and ASDR, along with higher AAPCs. This means that while the burden is still higher in higher SDI regions, improvements in disease deaths and DALYs have been more pronounced over the past three decades. Fourth, geographically, Europe and the Caribbean demonstrated higher ASMR or ASDR, whereas Western Sub-Saharan Africa and South Asia exhibited lower rates. Finally, projections suggest that over the next two decades, the global ASMR and ASDR will continue to steadily decline.

Both the mortality and DALYs rates of urological cancers attributable to smoking have significantly declined in the past three decades. This positive trend can be largely attributed to the successful implementation of the World Health Organization (WHO) Framework Convention on Tobacco Control (FCTC). The FCTC’s comprehensive tobacco control measures, including increased tobacco taxation, mandatory health warnings on packaging, complete bans on tobacco advertising and sponsorship, and restrictions on public smoking, have collectively contributed to a reduction in global smoking prevalence^18^. In China, the overall smoking rate among women decreased from 7% in 1984 to 2% in 2018, while that among men declined from 61% to 50% during the same period^19^. Advancements in cancer care have played a crucial role in improving patient outcomes. The implementation of risk stratification strategies has enabled the identification of patients at a higher risk of cancer. The development of electronic risk prediction tools that integrate sociodemographic and clinical characteristics from medical records, and the use of molecular techniques and genomic information to predict cancer recurrence, progression, and metastasis, have significantly enhanced patient survival rates and quality of life^20-22^. Moreover, the implementation of innovative clinical trial designs incorporating enhanced decentralized approaches combined with the establishment of robust patient navigation systems and strategic community partnerships and the systematic adoption of quality equity metrics that specifically monitor treatment timelines and technology utilization across diverse sociodemographic groups, have effectively addressed racial and geographic disparities in diagnostic imaging and treatment for patients with urological cancer, ultimately advancing care equity^23,24^.

According to GLOBOCAN 2022, the ASMR ratio between males and females for bladder cancer is 3.1:0.8 and that for kidney cancer is 2.0:0.9^2^. In comparison, the 2021 study showed that bladder cancer attributable to smoking has an ASMR ratio of 1.45:0.12 between males and females, whereas kidney cancer attributable to smoking has an ASMR ratio of 0.36:0.04. These data indicate that smoking exacerbates sex disparities in patients with cancer. Globally, smoking patterns differ between men and women; the smoking rate among men is five times higher than that among women. Notably, there were regional variations in smoking patterns. In Central Europe, both male and female smoking rates are significantly higher than the global average, while men in South-East Asia have the highest smoking rates and women in Western Europe rank first globally^25^. In this study, the regional distribution of the disease burden of urological cancers attributable to smoking aligned with the above findings, with Central Europe having the highest ASDR and ASMR. Women are often more receptive to health education, indicating that they have stronger health awareness and are more likely to follow medical advice than men^26^. This significant sex disparity highlights the need for sex-specific public health interventions. Notably higher smoking, mortality, and DALYs rates among men underscore the urgent need for tailored smoking cessation programs for male patients. Additionally, men typically start smoking earlier than women, which also leads to earlier or comparable peaks in mortality and DALYs rates among men^27^. These findings emphasize the importance of early interventions and preventive measures targeting men.

In 2021, there was a nonlinear positive correlation between SDI, ASMR, and ASDR at the national and regional levels, indicating that countries with higher SDI often bear a greater burden of deaths and DALYs from urologic cancers attributable to smoking. The unfavorable patterns of urologic cancers observed in high-SDI regions may be associated with other risk factors such as obesity, alcohol consumption, and hypertension^28-30^. However, over the past three decades, the declining rates of ASMR and ASDR in higher SDI regions have been faster than those in lower SDI regions. This is attributed to the fact that many higher SDI countries, including Australia, Canada, South Korea, and the United States, achieved significant reductions in smoking rates even before the adoption of the FCTC. In addition, sustained economic growth, improved public health awareness, and the establishment of relatively comprehensive basic medical insurance systems have effectively ensured the treatment of patients with cancer attributable to smoking^31,32^. In countries with a lower SDI, the disease burden of urological cancers attributable to smoking, although declining, remains severe. Studies have reported that, compared to global consumption, high-income and European countries have reduced annual cigarette consumption per adult by more than 1000 cigarettes, while low- and middle-income and Asian countries have increased consumption by more than 500 cigarettes^33^. Moreover, smoking cessation rates in low- and middle-income countries are extremely low, casting a shadow over national-level tobacco control efforts^34^. In the future, well-resourced, comprehensive, and multifaceted cancer control plans are needed to narrow the economic and geographical disparities in the shortest possible time.

### Strengths and limitations

This study provides a comprehensive analysis of the global burdens of bladder cancer, kidney cancer, and PCa attributable to smoking from 1990 to 2021 based on GBD data, offering critical insights for reducing mortality, formulating health policies, and improving healthcare services. However, this study has several limitations. First, the GBD data lack comprehensive population coverage in remote areas, and variations in diagnostic criteria across regions may introduce biases, potentially distorting the assessment of the actual disease burden, particularly in underserved regions. This could lead to the misallocation of healthcare resources or biased risk factor evaluations. Second, the etiology of cancers attributable to smoking is complex and involves genetic, environmental, and chemical factors. However, the GBD data only include a subset of these risk factors, which may result in incomplete etiological analysis and hinder the development of comprehensive prevention and intervention strategies. Third, the absence of pathological type and clinical staging information in the GBD data limits the in-depth analysis of disease severity, prognosis, and treatment efficacy, potentially affecting the applicability of the findings in clinical practice. However, the lack of raw data in some countries may lead to discrepancies between the results and the actual situation due to the model’s reliance on indirect data and assumptions. Particularly, high-quality data from low- and middle-income regions are limited, potentially underestimating the true disease burden. Fourth, the study only analyzed data at the national level and did not include subnational data, which may have overlooked significant regional variations. Future research should consider incorporating subnational data and other global databases to reduce the errors caused by differences in data collection methods, thereby enhancing the accuracy and comprehensiveness of the results. Fifth, our study only focused on the burden of urological cancers caused by smoking, while neglecting other confounding factors such as obesity, genetics, high blood pressure, alcohol, and environmental factors. Sixth, the Joinpoint analysis may underestimate the uncertainty of the AAPC trend because the uncertainty of the GBD method was not considered in the regression analysis. Moreover, the Joinpoint regression model may be sensitive to outliers, which may affect the estimated location of the join point. Seventh, our analysis was restricted to smoked tobacco products, excluding other forms such as smokeless tobacco, chewed tobacco, secondhand smoking, and e-cigarettes. Finally, the smoking data relied on self-reported information and cancer registry records, both of which are subject to potential reporting biases and inaccuracies.

## CONCLUSIONS

The global burden of bladder cancer, kidney cancer, and PCa attributable to smoking increased significantly from 1990 to 2021, with notable disparities across the SDI regions and sexes. High-SDI regions exhibited higher disease rates than low-SDI regions, reflecting differences in healthcare access and prevalence of risk factors. Males bear a disproportionately higher burden than females, likely due to higher smoking rates and lower cessation rates. The growing burden highlights the need for more targeted tobacco control policies and public health interventions based on the geographical, age and population distribution of the diseases. Future efforts should focus on tailoring prevention strategies and allocating resources to mitigate the global impacts of these cancers.

## Supplementary Material



## Data Availability

The data supporting this research are available from the Global Health Data Exchange (GHDx) query tool (http://ghdx.healthdata.org/gbdresults-tool).
