# Supplementary material for: The global burden of bladder, kidney, and prostate cancers attributable to smoking from 1990 to 2021 and projections for the next two decades: A cross-sectional study
Source: Tob Induc Dis. 2025 May 23;23:10.18332/tid/204299. doi: 10.18332/tid/204299 (PMC12101059; doi:10.18332/tid/204299)
Supplement: Supplementary file 1 [file TID-23-69-s1.pdf]

Supplementary Materials

STROBE Statement—Checklist of items that should be included in reports of cross-sectional studies

Supplementary Table 1. Average annual percentage change (AAPC) and estimated annual percentage change (EAPC) of age-standardized mortality rates (ASMR) and age-standardized disability-adjusted life year rates (ASDR) for bladder cancer attributable to smoking.

Supplementary Table 2. The global burden of bladder cancer attributable to smoking in 2021.

Supplementary Table 3. Decomposition analysis of bladder, kidney, and prostate cancers attributable to smoking.

Supplementary Table 4. Autoregressive integrated moving average (ARIMA) model predicts disease burden over the next 20 years.

Supplementary Table 5. Global average annual percentage change (AAPC) of age-standardized mortality rates (ASMR) and age-standardized disability-adjusted life year rates (ASDR) calculated for different time windows (10, 15, 20, and 25 years).

Supplementary Table 6. Average annual percentage change (AAPC) and estimated annual percentage change (EAPC) of age-standardized mortality rates (ASMR) and age-standardized disability-adjusted life year rates (ASDR) for kidney cancer attributable to smoking.

Supplementary Table 7. The global burden of kidney cancer attributable to smoking in 2021.

Supplementary Table 8. Average annual percentage change (AAPC) and estimated annual percentage change (EAPC) of age-standardized mortality rates (ASMR) and age-standardized disability-adjusted life year rates (ASDR) for prostate cancer attributable to smoking.

Supplementary Table 9. The global burden of prostate cancer attributable to smoking in 2021.

Supplementary Figure 1. The number of (A) bladder cancer deaths, (D) bladder cancer DALYs, (G) kidney cancer deaths, (J) kidney cancer DALYs, (M) PCa deaths, and (P) PCa DALYs in men or women by age. Temporal trends in (B) the number of deaths and ASMR in bladder cancer, (E) the number of DALYs and ASDR in bladder cancer, (H) the number of deaths and ASMR in kidney cancer, (K) the number of DALYs and ASDR in kidney cancer, (N) the number of deaths and ASMR in PCa, and (Q) the number of DALYs and ASDR in PCa. Rates of (C) mortality for bladder cancer, (F) DALYs for bladder cancer, (I) mortality for kidney cancer, (L) DALYs for kidney cancer, (O) mortality for PCa, and (R) DALYs for PCa by age. DALYs, disability-adjusted life years; PCa, prostate cancer; ASMR, age-standardised

mortality rate; ASDR, age-standardised DALYs rate.

Supplementary Figure 2. The joinpoint regression analysis of (A) ASMR for bladder cancer, (B) ASDR for bladder cancer, (C) ASMR for kidney cancer, (D) ASDR for kidney cancer, (E) ASMR for PCa, (F) ASDR for PCa. ASMR, age-standardised mortality rate; ASDR, age-standardised disability-adjusted life years rate; PCa, prostate cancer.

Supplementary Figure 3. Decomposition analysis of rates of **(A)** mortality in bladder cancer, **(B)** DALYs in bladder cancer, **(C)** mortality in kidney cancer, **(D)** DALYs in kidney cancer, **(E)** mortality in PCa, and **(F)** DALYs in PCa. DALYs, disability-adjusted life years; PCa, prostate cancer.

Supplementary Figure 4. The associations between the SDI and disease burden across GBD regions or countries. (A) Association between ASMR and SDI in bladder cancer across GBD regions; (B) association between ASMR and SDI in bladder cancer across GBD countries; (C) association between ASDR and SDI in bladder cancer across GBD regions; (D) association between ASDR and SDI in bladder cancer across GBD countries; (E) association between ASMR and SDI in kidney cancer across GBD regions; (F) association between ASMR and SDI in kidney cancer across GBD countries; (G) association between ASDR and SDI in kidney cancer across GBD regions; (H) association between ASDR and SDI in kidney cancer across GBD countries; (I) association between ASMR and SDI in PCa across GBD regions; (J) association between ASMR and SDI in PCa across GBD countries; (K) association between ASDR and SDI in PCa across GBD regions; (L) association between ASDR and SDI in PCa across GBD countries. SDI, socio-demographic index; GBD, Global Burden of Disease; ASMR, age-standardised mortality rate; ASDR, age-standardised disability-adjusted life years rate; PCa, prostate cancer.

Supplementary Figure 5. The ARIMA model predicts (A) ASMR in bladder cancer, (B) ASDR in bladder cancer, (C) ASMR in kidney cancer, (D) ASDR in kidney cancer, (E) ASMR in PCa, and (F) ASDR in PCa over the next two decades. ARIMA, autoregressive integrated moving average; ASMR, age-standardised mortality rate; ASDR, age-standardised disability-adjusted life years rate; PCa, prostate cancer.

STROBE Statement—Checklist of items that should be included in reports of *cross-sectional studies*

	Item No	Recommendation	Page
**Title and abstract**	1	(*a*) Indicate the study's design with a commonly used term in the title or the abstract	P1
		(*b*) Provide in the abstract an informative and balanced summary of what was done and what was found	P1-P2
**Introduction**			
Background/rationale	2	Explain the scientific background and rationale for the investigation being reported	P3-P4
Objectives	3	State specific objectives, including any prespecified hypotheses	P4-P5
**Methods**			
Study design	4	Present key elements of study design early in the paper	P5-P7
Setting	5	Describe the setting, locations, and relevant dates, including periods of recruitment, exposure, follow-up, and data collection	P5-P7
Participants	6	(*a*) Give the eligibility criteria, and the sources and methods of selection of participants	P5-P7
Variables	7	Clearly define all outcomes, exposures, predictors, potential confounders, and effect modifiers. Give diagnostic criteria, if applicable	P6
Data sources/ measurement	8*	For each variable of interest, give sources of data and details of methods of assessment (measurement). Describe comparability of assessment methods if there is more than one group	P5-P7
Bias	9	Describe any efforts to address potential sources of bias	P8-P9
Study size	10	Explain how the study size was arrived at	P5-P6

Quantitative variables	11	Explain how quantitative variables were handled in the analyses. If applicable, describe which groupings were chosen and why	P7
Statistical methods	12	(*a*) Describe all statistical methods, including those used to control for confounding	P7-P10
		(*b*) Describe any methods used to examine subgroups and interactions	P7-P8
		(*c*) Explain how missing data were addressed	P7-P8
		(*d*) If applicable, describe analytical methods taking account of sampling strategy	Not applicable
		(*e*) Describe any sensitivity analyses	P8-P9
**Results**			
Participants	13*	(a) Report numbers of individuals at each stage of study—eg numbers potentially eligible, examined for eligibility, confirmed eligible, included in the study, completing follow-up, and analysed	P10, P13, P16
		(b) Give reasons for non-participation at each stage	Not applicable
		(c) Consider use of a flow diagram	Not applicable
Descriptive data	14*	(a) Give characteristics of study participants (eg demographic, clinical, social) and information on exposures and potential confounders	P10-P19
		(b) Indicate number of participants with missing data for each variable of interest	Not applicable
Outcome data	15*	Report numbers of outcome events or summary measures	P10-P19
Main results	16	(*a*) Give unadjusted estimates and, if applicable, confounder-adjusted	Not

		estimates and their precision (eg, 95% confidence interval). Make clear which confounders were adjusted for and why they were included	applicable
		(*b*) Report category boundaries when continuous variables were categorized	P10-P19
		(*c*) If relevant, consider translating estimates of relative risk into absolute risk for a meaningful time period	Not applicable
Other analyses	17	Report other analyses done—eg analyses of subgroups and interactions, and sensitivity analyses	
**Discussion**			
Key results	18	Summarise key results with reference to study objectives	P19-P20
Limitations	19	Discuss limitations of the study, taking into account sources of potential bias or imprecision. Discuss both direction and magnitude of any potential bias	P23-P24
Interpretation	20	Give a cautious overall interpretation of results considering objectives, limitations, multiplicity of analyses, results from similar studies, and other relevant evidence	P20-P22
Generalisability	21	Discuss the generalisability (external validity) of the study results	P21-P22
**Other information**			
Funding	22	Give the source of funding and the role of the funders for the present study and, if applicable, for the original study on which the present article is based	P25

*Give information separately for exposed and unexposed groups.

Note: An Explanation and Elaboration article discusses each checklist item and gives methodological background and published examples of transparent reporting. The STROBE checklist is best used in conjunction with this article (freely available on the Web sites of PLoS Medicine at http://www.plosmedicine.org/, Annals of Internal Medicine at http://www.annals.org/, and Epidemiology at http://www.epidem.com/). Information on the STROBE Initiative is available at www.strobe-statement.org.

Supplementary Table 1. Average annual percentage change (AAPC) and estimated annual percentage change (EAPC) of age-standardized mortality rates (ASMR) and age-standardized disability-adjusted life year rates (ASDR) for bladder cancer attributable to smoking.

location_name	AAPC of ASMR (95% CI)	EAPC of ASMR (95% CI)	AAPC of ASDR (95% CI)	EAPC of ASDR (95% CI)
Afghanistan	0.37 (0.23,0.51)	0.4 (0.13,0.67)	0.34 (0.19,0.48)	0.31 (0.03,0.58)
Albania	-0.25 (-0.33,-0.16)	0.1 (-0.07,0.26)	-0.39 (-0.47,-0.31)	-0.03 (-0.19,0.13)
Algeria	-0.98 (-1.10,-0.86)	-0.73 (-0.97,-0.49)	-0.97 (-1.06,-0.89)	-0.91 (-1.07,-0.75)
American Samoa	0.31 (0.16,0.45)	0.79 (0.51,1.07)	0.44 (0.29,0.58)	0.95 (0.66,1.23)
Andean Latin America	-0.89 (-0.99,-0.79)	-0.91 (-1.1,-0.73)	-0.93 (-1.03,-0.84)	-0.98 (-1.16,-0.8)

Andorra	-2.24 (-2.38,-2.10)	-2.15 (-2.41,-1.88)	-2.25 (-2.39,-2.12)	-2.13 (-2.37,-1.88)
Angola	-0.46 (-0.59,-0.33)	-0.32 (-0.57,-0.08)	-0.50 (-0.64,-0.37)	-0.37 (-0.63,-0.11)
Antigua and Barbuda	-0.19 (-0.36,-0.03)	0.03 (-0.28,0.35)	-0.35 (-0.50,-0.19)	-0.18 (-0.48,0.12)
Argentina	-2.11 (-2.18,-2.04)	-1.89 (-2.02,-1.76)	-2.14 (-2.21,-2.06)	-1.98 (-2.11,-1.84)
Armenia	0.20 (0.06,0.35)	0.26 (-0.01,0.53)	-0.02 (-0.16,0.13)	-0.03 (-0.31,0.25)
Australasia	-2.85 (-2.90,-2.79)	-2.96 (-3.06,-2.86)	-3.10 (-3.16,-3.05)	-3.21 (-3.32,-3.1)
Australia	-3.09 (-3.13,-3.06)	-3.15 (-3.21,-3.09)	-3.32 (-3.36,-3.28)	-3.36 (-3.43,-3.29)
Austria	-1.46 (-1.51,-1.41)	-1.33 (-1.43,-1.24)	-1.54 (-1.60,-1.49)	-1.35 (-1.46,-1.25)
Azerbaijan	-0.11 (-0.22,0.00)	0.14 (-0.07,0.35)	-0.41 (-0.51,-0.31)	-0.35 (-0.53,-0.16)
Bahamas	0.05 (-0.04,0.13)	0.44 (0.26,0.61)	-0.18 (-0.27,-0.08)	0.24 (0.06,0.42)

Bahrain	-1.86 (-2.00,-1.73)	-2.38 (-2.62,-2.13)	-1.99 (-2.10,-1.87)	-2.52 (-2.74,-2.31)
Bangladesh	-1.24 (-1.37,-1.11)	-1.55 (-1.79,-1.3)	-1.55 (-1.64,-1.45)	-1.74 (-1.91,-1.56)
Barbados	-0.59 (-0.73,-0.46)	-0.67 (-0.92,-0.41)	-0.79 (-0.91,-0.67)	-0.81 (-1.03,-0.58)
Belarus	-0.94 (-1.17,-0.72)	-1.74 (-2.16,-1.31)	-0.95 (-1.17,-0.73)	-1.77 (-2.18,-1.35)
Belgium	-2.55 (-2.66,-2.43)	-2.52 (-2.74,-2.3)	-2.53 (-2.65,-2.40)	-2.54 (-2.78,-2.31)
Belize	0.12 (-0.10,0.34)	0.45 (0.03,0.87)	0.11 (-0.11,0.32)	0.42 (0.01,0.83)
Benin	-2.33 (-2.47,-2.20)	-2.86 (-3.11,-2.61)	-2.27 (-2.40,-2.14)	-2.78 (-3.03,-2.53)
Bermuda	-1.33 (-1.48,-1.18)	-0.77 (-1.05,-0.49)	-1.38 (-1.54,-1.23)	-0.88 (-1.16,-0.59)
Bhutan	-0.37 (-0.43,-0.31)	-0.43 (-0.54,-0.32)	-0.67 (-0.74,-0.61)	-0.76 (-0.88,-0.64)
Bolivia (Plurinational State of)	-0.91 (-1.02,-0.80)	-0.54 (-0.76,-0.32)	-1.06 (-1.18,-0.95)	-0.72 (-0.94,-0.5)

Bosnia and Herzegovina	0.98 (0.85,1.11)	1.31 (1.06,1.56)	0.84 (0.71,0.98)	1.2 (0.94,1.46)
Botswana	-0.88 (-0.98,-0.78)	-1.17 (-1.36,-0.98)	-0.91 (-1.04,-0.79)	-1.26 (-1.5,-1.02)
Brazil	-2.04 (-2.08,-2.00)	-2.13 (-2.21,-2.05)	-2.12 (-2.17,-2.07)	-2.29 (-2.38,-2.2)
Brunei Darussalam	-2.24 (-2.41,-2.08)	-1.58 (-1.89,-1.27)	-2.33 (-2.48,-2.18)	-1.86 (-2.14,-1.57)
Bulgaria	0.38 (0.31,0.46)	0.68 (0.54,0.82)	0.45 (0.38,0.52)	0.64 (0.5,0.78)
Burkina Faso	-0.74 (-0.83,-0.66)	-1 (-1.16,-0.85)	-0.74 (-0.84,-0.65)	-1.04 (-1.21,-0.86)
Burundi	-2.57 (-2.81,-2.34)	-2.78 (-3.22,-2.34)	-2.63 (-2.87,-2.38)	-2.85 (-3.29,-2.4)
Cabo Verde	6.10 (5.34,6.86)	4.19 (2.69,5.71)	6.04 (5.28,6.79)	4.24 (2.74,5.75)
Cambodia	-0.24 (-0.33,-0.15)	-0.4 (-0.57,-0.23)	-0.36 (-0.46,-0.27)	-0.58 (-0.76,-0.4)
Cameroon	-1.02 (-1.12,-0.93)	-1.26 (-1.44,-1.07)	-0.91 (-1.02,-0.80)	-1.15 (-1.36,-0.94)

Canada	-2.82 (-2.89,-2.74)	-2.9 (-3.04,-2.76)	-3.13 (-3.22,-3.05)	-3.23 (-3.4,-3.07)
Caribbean	-0.72 (-0.77,-0.66)	-0.56 (-0.67,-0.46)	-0.64 (-0.70,-0.59)	-0.48 (-0.59,-0.37)
Central African Republic	-1.25 (-1.35,-1.14)	-1.34 (-1.53,-1.15)	-1.22 (-1.33,-1.12)	-1.34 (-1.54,-1.14)
Central Asia	-0.15 (-0.25,-0.05)	-0.15 (-0.34,0.04)	-0.54 (-0.66,-0.43)	-0.67 (-0.89,-0.44)
Central Europe	-0.20 (-0.26,-0.15)	-0.25 (-0.36,-0.15)	-0.31 (-0.37,-0.25)	-0.35 (-0.47,-0.24)
Central Europe, Eastern Europe, and Central Asia	-0.37 (-0.51,-0.22)	-0.62 (-0.9,-0.34)	-0.51 (-0.66,-0.36)	-0.8 (-1.09,-0.51)
Central Latin America	-2.33 (-2.38,-2.28)	-2.55 (-2.65,-2.45)	-2.28 (-2.33,-2.22)	-2.52 (-2.62,-2.41)
Central Sub-Saharan Africa	-0.55 (-0.73,-0.38)	-0.46 (-0.8,-0.13)	-0.51 (-0.68,-0.34)	-0.41 (-0.73,-0.09)
Chad	-0.31 (-0.45,-0.17)	-0.81 (-1.08,-0.54)	-0.24 (-0.39,-0.10)	-0.72 (-1,-0.44)
Chile	-1.89 (-1.95,-1.82)	-1.74 (-1.86,-1.62)	-1.96 (-2.02,-1.90)	-1.79 (-1.9,-1.67)

China	-1.14 (-1.20,-1.07)	-1.36 (-1.49,-1.24)	-1.26 (-1.32,-1.19)	-1.48 (-1.61,-1.35)
Colombia	-2.95 (-3.09,-2.82)	-3.48 (-3.73,-3.22)	-3.08 (-3.22,-2.94)	-3.64 (-3.89,-3.38)
Comoros	-0.73 (-0.79,-0.67)	-1.08 (-1.2,-0.96)	-0.98 (-1.06,-0.90)	-1.38 (-1.54,-1.23)
Congo	0.13 (-0.01,0.27)	0.25 (-0.02,0.52)	-0.06 (-0.20,0.08)	0.02 (-0.25,0.29)
Cook Islands	-0.75 (-0.78,-0.72)	-0.77 (-0.82,-0.71)	-0.71 (-0.74,-0.68)	-0.69 (-0.75,-0.63)
Costa Rica	-1.73 (-1.86,-1.60)	-2.05 (-2.29,-1.81)	-1.71 (-1.82,-1.60)	-2.11 (-2.31,-1.91)
Croatia	-0.45 (-0.55,-0.35)	0 (-0.2,0.19)	-0.35 (-0.43,-0.27)	0 (-0.15,0.15)
Cuba	-0.42 (-0.49,-0.36)	-0.29 (-0.41,-0.16)	-0.33 (-0.40,-0.26)	-0.17 (-0.3,-0.04)
Cyprus	-2.01 (-2.06,-1.96)	-1.96 (-2.05,-1.86)	-1.54 (-1.59,-1.49)	-1.36 (-1.45,-1.26)
Czechia	-1.06 (-1.13,-1.00)	-1.18 (-1.3,-1.07)	-1.19 (-1.24,-1.14)	-1.3 (-1.39,-1.2)

C 么 te d'Ivoire	-0.44 (-0.61,-0.27)	-0.94 (-1.26,-0.62)	-0.45 (-0.62,-0.27)	-0.96 (-1.29,-0.62)
Democratic People's Republic of Korea	-0.63 (-0.66,-0.60)	-0.61 (-0.67,-0.56)	-0.43 (-0.45,-0.40)	-0.42 (-0.46,-0.37)
Democratic Republic of the Congo	-0.88 (-1.09,-0.67)	-0.85 (-1.24,-0.46)	-0.74 (-0.94,-0.55)	-0.69 (-1.07,-0.32)
Denmark	-1.60 (-1.94,-1.27)	-2.77 (-3.39,-2.14)	-1.81 (-2.13,-1.50)	-2.94 (-3.53,-2.35)
Djibouti	0.10 (0.04,0.15)	0.19 (0.08,0.29)	-0.06 (-0.12,0.00)	0.01 (-0.11,0.12)
Dominica	-0.45 (-0.52,-0.38)	-0.35 (-0.48,-0.21)	-0.39 (-0.48,-0.31)	-0.23 (-0.38,-0.07)
Dominican Republic	-0.41 (-0.58,-0.24)	-0.12 (-0.44,0.21)	-0.24 (-0.38,-0.10)	0 (-0.26,0.27)
East Asia	-1.12 (-1.18,-1.05)	-1.35 (-1.47,-1.23)	-1.24 (-1.31,-1.18)	-1.47 (-1.6,-1.35)
Eastern Europe	-0.58 (-0.81,-0.36)	-1.03 (-1.45,-0.6)	-0.65 (-0.87,-0.42)	-1.15 (-1.58,-0.73)
Eastern Sub-Saharan Africa	-0.97 (-1.01,-0.93)	-1.18 (-1.26,-1.1)	-0.93 (-0.97,-0.90)	-1.15 (-1.22,-1.07)

Ecuador	-1.06 (-1.23,-0.89)	-0.85 (-1.17,-0.53)	-1.08 (-1.24,-0.92)	-0.99 (-1.29,-0.69)
Egypt	-1.58 (-1.70,-1.45)	-2.01 (-2.24,-1.78)	-1.98 (-2.15,-1.82)	-2.56 (-2.86,-2.26)
El Salvador	0.31 (0.24,0.38)	0.34 (0.2,0.47)	0.42 (0.34,0.49)	0.45 (0.3,0.59)
Equatorial Guinea	0.11 (-0.08,0.30)	0.22 (-0.14,0.59)	-0.13 (-0.34,0.07)	-0.09 (-0.48,0.3)
Eritrea	-0.71 (-0.80,-0.63)	-1.08 (-1.24,-0.92)	-0.93 (-1.02,-0.84)	-1.33 (-1.5,-1.16)
Estonia	-0.50 (-0.69,-0.31)	-1.01 (-1.37,-0.65)	-0.76 (-0.96,-0.57)	-1.27 (-1.64,-0.89)
Eswatini	-1.04 (-1.21,-0.88)	-0.81 (-1.12,-0.5)	-0.88 (-1.07,-0.70)	-0.75 (-1.1,-0.4)
Ethiopia	-1.23 (-1.44,-1.02)	-1.05 (-1.44,-0.66)	-1.61 (-1.82,-1.40)	-1.52 (-1.92,-1.12)
Fiji	-0.53 (-0.61,-0.45)	-0.37 (-0.52,-0.22)	-0.56 (-0.65,-0.47)	-0.36 (-0.54,-0.19)
Finland	-2.61 (-2.67,-2.56)	-2.87 (-2.97,-2.77)	-2.72 (-2.77,-2.67)	-2.95 (-3.05,-2.85)

France	-1.90 (-1.92,-1.88)	-1.83 (-1.87,-1.79)	-1.95 (-1.98,-1.93)	-1.84 (-1.88,-1.79)
Gabon	-0.02 (-0.05,0.01)	-0.09 (-0.15,-0.03)	-0.02 (-0.05,0.00)	-0.05 (-0.1,0)
Gambia	-1.00 (-1.08,-0.93)	-1.24 (-1.38,-1.1)	-1.06 (-1.15,-0.98)	-1.33 (-1.49,-1.17)
Georgia	1.59 (1.31,1.87)	2.05 (1.5,2.6)	1.25 (0.99,1.51)	1.62 (1.12,2.12)
Germany	-2.15 (-2.28,-2.03)	-2.31 (-2.54,-2.08)	-2.15 (-2.26,-2.05)	-2.26 (-2.46,-2.06)
Ghana	-0.41 (-0.46,-0.35)	-0.26 (-0.36,-0.16)	-0.43 (-0.48,-0.38)	-0.29 (-0.39,-0.19)
Greece	-1.25 (-1.33,-1.17)	-1.34 (-1.49,-1.18)	-1.19 (-1.27,-1.11)	-1.17 (-1.32,-1.02)
Greenland	-1.95 (-2.01,-1.89)	-1.76 (-1.87,-1.64)	-2.05 (-2.09,-2.00)	-1.86 (-1.95,-1.78)
Grenada	-0.31 (-0.59,-0.03)	-0.35 (-0.88,0.18)	-0.39 (-0.59,-0.19)	-0.33 (-0.72,0.06)
Guam	-0.29 (-0.45,-0.14)	0.68 (0.38,0.98)	0.10 (-0.06,0.26)	1.06 (0.76,1.37)

Guatemala	-1.89 (-2.02,-1.76)	-2.27 (-2.51,-2.03)	-1.69 (-1.82,-1.56)	-2.03 (-2.27,-1.78)
Guinea	0.32 (0.28,0.36)	0.38 (0.31,0.45)	0.42 (0.37,0.47)	0.54 (0.44,0.64)
Guinea-Bissau	0.08 (-0.05,0.20)	0.31 (0.07,0.55)	0.17 (0.06,0.29)	0.44 (0.21,0.67)
Guyana	-0.65 (-0.76,-0.55)	-0.01 (-0.21,0.2)	-0.58 (-0.69,-0.47)	0.06 (-0.15,0.28)
Haiti	-1.06 (-1.12,-1.00)	-0.95 (-1.06,-0.83)	-1.32 (-1.40,-1.25)	-1.25 (-1.39,-1.1)
High SDI	-1.77 (-1.79,-1.75)	-1.85 (-1.89,-1.82)	-1.88 (-1.89,-1.86)	-1.94 (-1.96,-1.91)
High-income	-2.02 (-2.03,-2.01)	-2.06 (-2.08,-2.04)	-2.12 (-2.13,-2.10)	-2.13 (-2.16,-2.1)
High-income Asia Pacific	-1.69 (-1.73,-1.64)	-1.84 (-1.92,-1.75)	-1.78 (-1.82,-1.74)	-1.89 (-1.96,-1.81)
High-income North America	-1.18 (-1.23,-1.12)	-1.15 (-1.26,-1.05)	-1.37 (-1.44,-1.29)	-1.36 (-1.5,-1.22)
High-middle SDI	-1.34 (-1.40,-1.28)	-1.55 (-1.66,-1.43)	-1.50 (-1.56,-1.43)	-1.73 (-1.84,-1.61)

Honduras	0.81 (0.73,0.89)	1.1 (0.94,1.26)	0.69 (0.61,0.76)	0.92 (0.78,1.07)
Hungary	-0.61 (-0.76,-0.45)	-0.65 (-0.94,-0.36)	-0.47 (-0.63,-0.31)	-0.55 (-0.86,-0.25)
Iceland	-2.67 (-2.75,-2.59)	-2.78 (-2.92,-2.63)	-2.75 (-2.83,-2.68)	-2.86 (-3,-2.72)
India	-0.87 (-0.94,-0.80)	-1.09 (-1.22,-0.96)	-1.05 (-1.12,-0.98)	-1.3 (-1.44,-1.17)
Indonesia	0.42 (0.34,0.49)	0.35 (0.21,0.49)	0.30 (0.24,0.35)	0.23 (0.12,0.34)
Iran (Islamic Republic of)	-0.14 (-0.21,-0.06)	0.19 (0.05,0.33)	-0.25 (-0.31,-0.18)	0.05 (-0.07,0.18)
Iraq	0.28 (0.18,0.37)	-0.06 (-0.25,0.13)	0.06 (-0.02,0.14)	-0.26 (-0.41,-0.1)
Ireland	-2.92 (-3.00,-2.83)	-2.96 (-3.12,-2.8)	-3.01 (-3.08,-2.93)	-3.03 (-3.18,-2.89)
Israel	-1.77 (-1.92,-1.62)	-1.93 (-2.21,-1.65)	-1.81 (-1.96,-1.65)	-1.88 (-2.17,-1.59)
Italy	-2.48 (-2.52,-2.44)	-2.59 (-2.66,-2.51)	-2.63 (-2.67,-2.60)	-2.68 (-2.75,-2.61)

Jamaica	-0.46 (-0.65,-0.27)	-0.31 (-0.67,0.05)	-0.38 (-0.59,-0.17)	-0.27 (-0.68,0.13)
Japan	-1.62 (-1.66,-1.57)	-1.73 (-1.81,-1.64)	-1.59 (-1.64,-1.55)	-1.66 (-1.74,-1.58)
Jordan	-0.97 (-1.02,-0.91)	-1.06 (-1.16,-0.96)	-0.98 (-1.04,-0.91)	-1.08 (-1.2,-0.97)
Kazakhstan	-1.17 (-1.48,-0.86)	-1.77 (-2.35,-1.18)	-1.28 (-1.59,-0.96)	-1.97 (-2.56,-1.37)
Kenya	-0.34 (-0.41,-0.28)	-0.62 (-0.75,-0.49)	-0.15 (-0.23,-0.08)	-0.37 (-0.52,-0.22)
Kiribati	0.34 (0.23,0.44)	0.27 (0.07,0.47)	0.31 (0.21,0.40)	0.26 (0.08,0.45)
Kuwait	0.32 (0.02,0.61)	-0.6 (-1.16,-0.03)	0.17 (-0.14,0.47)	-0.87 (-1.45,-0.29)
Kyrgyzstan	-0.45 (-0.56,-0.34)	-0.65 (-0.86,-0.43)	-0.53 (-0.68,-0.38)	-0.87 (-1.15,-0.59)
Lao People's Democratic Republic	-0.57 (-0.64,-0.50)	-0.58 (-0.71,-0.45)	-0.85 (-0.92,-0.78)	-0.87 (-1,-0.74)
Latin America and Caribbean	-1.91 (-1.95,-1.88)	-2.01 (-2.08,-1.95)	-1.97 (-2.00,-1.93)	-2.11 (-2.18,-2.04)

Latvia	0.31 (0.19,0.43)	0.34 (0.11,0.57)	0.19 (0.06,0.32)	0.08 (-0.18,0.33)
Lebanon	-0.45 (-0.58,-0.31)	-0.03 (-0.3,0.23)	-0.64 (-0.76,-0.51)	-0.25 (-0.49,-0.01)
Lesotho	1.20 (1.11,1.30)	1.79 (1.6,1.98)	1.54 (1.43,1.64)	2.08 (1.87,2.3)
Liberia	-1.31 (-1.39,-1.23)	-1.71 (-1.86,-1.56)	-1.13 (-1.21,-1.05)	-1.49 (-1.64,-1.33)
Libya	0.36 (0.25,0.46)	0.83 (0.62,1.03)	0.38 (0.28,0.47)	0.73 (0.55,0.91)
Lithuania	-0.56 (-0.99,-0.13)	-1.31 (-2.11,-0.49)	-0.65 (-1.05,-0.26)	-1.41 (-2.15,-0.67)
Low SDI	-0.83 (-0.88,-0.77)	-1.04 (-1.15,-0.94)	-0.84 (-0.89,-0.79)	-1.09 (-1.19,-0.99)
Low-middle SDI	-1.09 (-1.14,-1.03)	-1.36 (-1.47,-1.25)	-1.17 (-1.24,-1.11)	-1.5 (-1.62,-1.37)
Luxembourg	-2.19 (-2.26,-2.12)	-2.08 (-2.21,-1.95)	-2.35 (-2.41,-2.28)	-2.26 (-2.39,-2.13)
Madagascar	-2.41 (-2.57,-2.24)	-2.55 (-2.85,-2.24)	-2.46 (-2.62,-2.30)	-2.6 (-2.89,-2.3)

Malawi	0.27 (0.18,0.37)	0.2 (0.02,0.39)	0.47 (0.35,0.59)	0.35 (0.12,0.58)
Malaysia	-0.50 (-0.60,-0.40)	-0.94 (-1.12,-0.75)	-0.63 (-0.71,-0.55)	-0.99 (-1.15,-0.83)
Maldives	-1.69 (-1.74,-1.64)	-1.97 (-2.08,-1.87)	-1.93 (-2.00,-1.85)	-2.32 (-2.47,-2.18)
Mali	1.05 (1.00,1.10)	1.34 (1.24,1.44)	0.95 (0.90,1.01)	1.24 (1.13,1.34)
Malta	-2.73 (-2.84,-2.62)	-2.77 (-2.97,-2.57)	-2.53 (-2.65,-2.41)	-2.48 (-2.69,-2.26)
Marshall Islands	0.63 (0.55,0.71)	0.94 (0.78,1.09)	0.56 (0.48,0.64)	0.84 (0.69,0.99)
Mauritania	-1.19 (-1.29,-1.09)	-1.6 (-1.78,-1.41)	-1.36 (-1.46,-1.27)	-1.76 (-1.94,-1.58)
Mauritius	-2.41 (-2.87,-1.95)	-1.54 (-2.41,-0.67)	-2.46 (-2.92,-2.01)	-1.67 (-2.52,-0.8)
Mexico	-2.64 (-2.69,-2.58)	-2.74 (-2.85,-2.63)	-2.48 (-2.54,-2.43)	-2.6 (-2.7,-2.49)
Micronesia (Federated States of)	0.09 (0.08,0.10)	0.12 (0.1,0.14)	0.18 (0.16,0.20)	0.25 (0.22,0.29)

Middle SDI	-1.07 (-1.13,-1.01)	-1.31 (-1.42,-1.2)	-1.23 (-1.28,-1.17)	-1.44 (-1.55,-1.34)
Monaco	-0.60 (-0.62,-0.59)	-0.59 (-0.62,-0.56)	-0.73 (-0.74,-0.71)	-0.73 (-0.76,-0.71)
Mongolia	-1.08 (-1.24,-0.92)	-1.54 (-1.85,-1.24)	-1.11 (-1.26,-0.96)	-1.55 (-1.84,-1.26)
Montenegro	0.33 (0.26,0.39)	0.33 (0.2,0.45)	0.15 (0.07,0.24)	0.16 (0,0.32)
Morocco	-0.73 (-0.82,-0.63)	-0.82 (-0.99,-0.64)	-0.56 (-0.67,-0.45)	-0.6 (-0.81,-0.39)
Mozambique	-0.16 (-0.22,-0.09)	0.26 (0.13,0.38)	0.03 (-0.05,0.11)	0.49 (0.34,0.64)
Myanmar	-2.18 (-2.24,-2.11)	-2.45 (-2.57,-2.33)	-2.41 (-2.48,-2.35)	-2.72 (-2.85,-2.6)
Namibia	-0.46 (-0.62,-0.29)	-0.71 (-1.02,-0.4)	-0.45 (-0.62,-0.29)	-0.73 (-1.04,-0.42)
Nauru	-0.53 (-0.61,-0.45)	-0.59 (-0.73,-0.44)	-0.28 (-0.38,-0.19)	-0.34 (-0.51,-0.16)
Nepal	-1.15 (-1.25,-1.04)	-1.4 (-1.6,-1.2)	-1.37 (-1.48,-1.26)	-1.65 (-1.86,-1.45)

Netherlands	-2.34 (-2.43,-2.25)	-2.3 (-2.47,-2.13)	-2.59 (-2.69,-2.49)	-2.54 (-2.73,-2.35)
New Zealand	-1.84 (-2.01,-1.66)	-2.16 (-2.49,-1.82)	-2.19 (-2.38,-2.00)	-2.57 (-2.92,-2.22)
Nicaragua	-0.52 (-0.62,-0.42)	-0.17 (-0.36,0.03)	-0.44 (-0.54,-0.34)	-0.13 (-0.31,0.06)
Niger	-0.72 (-0.85,-0.59)	-1.14 (-1.38,-0.9)	-1.03 (-1.13,-0.93)	-1.42 (-1.61,-1.23)
Nigeria	-1.48 (-1.51,-1.46)	-1.54 (-1.58,-1.49)	-1.27 (-1.33,-1.22)	-1.35 (-1.45,-1.25)
Niue	0.36 (0.31,0.42)	0.2 (0.09,0.3)	0.28 (0.23,0.33)	0.08 (-0.01,0.18)
North Africa and Middle East	-0.46 (-0.53,-0.40)	-1.18 (-1.26,-1.09)	-0.57 (-0.64,-0.49)	-1.44 (-1.54,-1.34)
North Macedonia	-0.08 (-0.25,0.09)	-0.19 (-0.52,0.13)	-0.20 (-0.34,-0.06)	-0.28 (-0.54,-0.01)
Northern Mariana Islands	1.04 (0.82,1.26)	1.82 (1.39,2.24)	1.08 (0.87,1.29)	1.91 (1.49,2.33)
Norway	-3.28 (-3.42,-3.15)	-3.79 (-4.04,-3.54)	-3.47 (-3.60,-3.33)	-3.93 (-4.17,-3.68)

Oceania	-0.43 (-0.45,-0.41)	-0.41 (-0.45,-0.38)	-0.32 (-0.33,-0.30)	-0.29 (-0.33,-0.26)
Oman	-1.16 (-1.30,-1.02)	-0.59 (-0.86,-0.32)	-1.37 (-1.49,-1.26)	-0.88 (-1.1,-0.66)
Pakistan	-0.68 (-0.81,-0.55)	-1 (-1.24,-0.75)	-0.68 (-0.81,-0.54)	-1.02 (-1.27,-0.76)
Palau	-0.74 (-0.80,-0.69)	-0.83 (-0.93,-0.73)	-0.65 (-0.71,-0.60)	-0.75 (-0.85,-0.65)
Palestine	-0.90 (-1.05,-0.76)	-1.19 (-1.47,-0.91)	-0.74 (-0.87,-0.62)	-0.99 (-1.23,-0.76)
Panama	-1.32 (-1.43,-1.20)	-1.76 (-1.98,-1.55)	-1.31 (-1.41,-1.21)	-1.73 (-1.92,-1.54)
Papua New Guinea	-0.24 (-0.28,-0.20)	-0.35 (-0.42,-0.27)	-0.17 (-0.21,-0.13)	-0.28 (-0.36,-0.2)
Paraguay	-0.26 (-0.31,-0.21)	-0.19 (-0.29,-0.09)	-0.21 (-0.26,-0.16)	-0.23 (-0.33,-0.14)
Peru	-0.76 (-0.91,-0.61)	-1.13 (-1.42,-0.85)	-0.77 (-0.92,-0.62)	-1.17 (-1.46,-0.88)
Philippines	-0.76 (-0.82,-0.70)	-0.65 (-0.76,-0.53)	-0.51 (-0.58,-0.44)	-0.55 (-0.69,-0.41)

Poland	-0.20 (-0.28,-0.13)	-0.39 (-0.53,-0.25)	-0.49 (-0.56,-0.41)	-0.61 (-0.75,-0.46)
Portugal	-1.53 (-1.58,-1.48)	-1.5 (-1.6,-1.4)	-1.23 (-1.29,-1.17)	-1.16 (-1.27,-1.05)
Puerto Rico	-1.29 (-1.40,-1.18)	-1.25 (-1.45,-1.05)	-1.02 (-1.12,-0.92)	-1.01 (-1.2,-0.82)
Qatar	-2.30 (-2.55,-2.04)	-2.35 (-2.83,-1.87)	-2.28 (-2.50,-2.05)	-2.17 (-2.59,-1.75)
Republic of Korea	-1.84 (-1.92,-1.76)	-2.22 (-2.38,-2.07)	-2.23 (-2.30,-2.17)	-2.56 (-2.68,-2.44)
Republic of Moldova	0.15 (0.02,0.27)	-0.09 (-0.32,0.15)	0.23 (0.11,0.35)	-0.04 (-0.27,0.2)
Romania	0.36 (0.27,0.44)	0.16 (-0.01,0.32)	0.26 (0.17,0.34)	0.06 (-0.1,0.22)
Russian Federation	-0.58 (-0.82,-0.34)	-1.06 (-1.52,-0.61)	-0.72 (-0.96,-0.49)	-1.27 (-1.72,-0.82)
Rwanda	-0.55 (-0.66,-0.45)	-1.13 (-1.33,-0.92)	-0.80 (-0.92,-0.68)	-1.46 (-1.69,-1.23)
Saint Kitts and Nevis	-0.79 (-0.90,-0.69)	-0.24 (-0.44,-0.04)	-0.96 (-1.07,-0.85)	-0.4 (-0.61,-0.19)

Saint Lucia	-1.49 (-1.59,-1.39)	-1.49 (-1.68,-1.3)	-1.47 (-1.54,-1.39)	-1.4 (-1.55,-1.26)
Saint Vincent and the Grenadines	-0.01 (-0.15,0.12)	0.32 (0.07,0.58)	-0.06 (-0.18,0.07)	0.28 (0.04,0.52)
Samoa	-0.55 (-0.60,-0.50)	-0.79 (-0.89,-0.69)	-0.57 (-0.62,-0.52)	-0.79 (-0.88,-0.69)
San Marino	-3.08 (-3.26,-2.89)	-2.2 (-2.56,-1.85)	-2.96 (-3.13,-2.80)	-2.2 (-2.51,-1.89)
Sao Tome and Principe	1.24 (1.10,1.38)	1.22 (0.94,1.49)	1.11 (0.97,1.24)	1.02 (0.76,1.28)
Saudi Arabia	-0.19 (-0.29,-0.08)	-0.46 (-0.66,-0.25)	-0.04 (-0.15,0.06)	-0.21 (-0.41,-0.01)
Senegal	-1.42 (-1.50,-1.33)	-1.82 (-1.99,-1.66)	-1.47 (-1.55,-1.38)	-1.87 (-2.02,-1.71)
Serbia	-0.47 (-0.56,-0.38)	-0.37 (-0.55,-0.19)	-0.25 (-0.35,-0.16)	-0.23 (-0.42,-0.04)
Seychelles	-1.10 (-1.22,-0.98)	-0.77 (-1,-0.53)	-1.25 (-1.36,-1.13)	-0.9 (-1.12,-0.69)
Sierra Leone	-1.67 (-1.70,-1.64)	-1.83 (-1.89,-1.77)	-1.58 (-1.60,-1.55)	-1.68 (-1.73,-1.63)

Singapore	-2.98 (-3.10,-2.85)	-2.95 (-3.19,-2.72)	-3.19 (-3.30,-3.08)	-3.11 (-3.31,-2.91)
Slovakia	-1.14 (-1.19,-1.09)	-0.9 (-1,-0.8)	-1.23 (-1.28,-1.18)	-1.02 (-1.11,-0.92)
Slovenia	-0.45 (-0.54,-0.36)	-0.51 (-0.68,-0.35)	-0.67 (-0.76,-0.57)	-0.72 (-0.9,-0.54)
Solomon Islands	0.07 (-0.00,0.14)	0.22 (0.08,0.36)	0.22 (0.14,0.31)	0.41 (0.25,0.57)
Somalia	-1.08 (-1.11,-1.05)	-1.07 (-1.13,-1.02)	-1.01 (-1.04,-0.99)	-1.04 (-1.09,-0.99)
South Africa	-1.51 (-1.62,-1.40)	-1.75 (-1.95,-1.55)	-1.35 (-1.45,-1.25)	-1.53 (-1.71,-1.35)
South Asia	-0.60 (-0.67,-0.54)	-1.54 (-1.62,-1.45)	-0.61 (-0.67,-0.56)	-1.56 (-1.64,-1.48)
South Sudan	-0.76 (-0.83,-0.70)	-0.97 (-1.1,-0.84)	-0.87 (-0.96,-0.77)	-1.12 (-1.29,-0.94)
Southeast Asia	-0.54 (-0.58,-0.50)	-0.81 (-0.88,-0.74)	-0.58 (-0.62,-0.55)	-0.82 (-0.89,-0.76)
Southeast Asia, East Asia, and Oceania	-0.96 (-1.02,-0.91)	-1.19 (-1.3,-1.08)	-1.14 (-1.20,-1.08)	-1.36 (-1.47,-1.24)

Southern Latin America	-2.12 (-2.17,-2.06)	-1.98 (-2.09,-1.88)	-2.18 (-2.23,-2.12)	-2.09 (-2.2,-1.98)
Southern Sub-Saharan Africa	-1.18 (-1.30,-1.06)	-1.35 (-1.58,-1.12)	-1.00 (-1.11,-0.89)	-1.14 (-1.35,-0.94)
Spain	-2.22 (-2.34,-2.11)	-2.16 (-2.38,-1.95)	-2.28 (-2.42,-2.14)	-2.26 (-2.52,-1.99)
Sri Lanka	-1.97 (-2.06,-1.88)	-1.64 (-1.81,-1.47)	-1.85 (-1.94,-1.76)	-1.63 (-1.81,-1.46)
Sub-Saharan Africa	-0.86 (-0.90,-0.81)	-1.01 (-1.1,-0.92)	-0.78 (-0.82,-0.73)	-0.92 (-1.01,-0.84)
Sudan	-0.71 (-0.74,-0.67)	-0.88 (-0.94,-0.81)	-0.81 (-0.85,-0.78)	-1.01 (-1.08,-0.94)
Suriname	-0.86 (-0.98,-0.73)	-0.62 (-0.86,-0.39)	-0.76 (-0.89,-0.64)	-0.56 (-0.79,-0.32)
Sweden	-1.37 (-1.49,-1.24)	-1.04 (-1.28,-0.81)	-1.58 (-1.72,-1.44)	-1.21 (-1.48,-0.94)
Switzerland	-0.19 (-0.34,-0.04)	-0.31 (-0.59,-0.01)	-0.52 (-0.67,-0.38)	-0.68 (-0.96,-0.4)
Syrian Arab Republic	-0.39 (-0.48,-0.30)	-0.65 (-0.82,-0.48)	-0.51 (-0.58,-0.44)	-0.79 (-0.92,-0.66)

Taiwan (Province of China)	-1.23 (-1.37,-1.08)	-1.76 (-2.03,-1.49)	-1.50 (-1.65,-1.35)	-2.14 (-2.42,-1.86)
Tajikistan	-2.08 (-2.23,-1.92)	-2.16 (-2.46,-1.87)	-2.16 (-2.31,-2.02)	-2.33 (-2.61,-2.05)
Thailand	-1.82 (-1.91,-1.73)	-2.41 (-2.58,-2.25)	-1.74 (-1.83,-1.66)	-2.29 (-2.44,-2.13)
Timor-Leste	-0.26 (-0.33,-0.19)	-0.24 (-0.38,-0.11)	-0.29 (-0.39,-0.20)	-0.26 (-0.45,-0.08)
Togo	-1.55 (-1.63,-1.47)	-1.98 (-2.12,-1.83)	-1.32 (-1.39,-1.24)	-1.71 (-1.85,-1.57)
Tokelau	-0.52 (-0.56,-0.48)	-0.59 (-0.66,-0.51)	-0.42 (-0.45,-0.39)	-0.48 (-0.53,-0.43)
Tonga	-0.05 (-0.14,0.04)	-0.16 (-0.33,0.02)	0.01 (-0.08,0.09)	-0.06 (-0.22,0.1)
Trinidad and Tobago	-1.22 (-1.31,-1.13)	-0.87 (-1.05,-0.7)	-1.06 (-1.15,-0.98)	-0.76 (-0.92,-0.59)
Tropical Latin America	-2.01 (-2.05,-1.97)	-2.1 (-2.18,-2.02)	-2.09 (-2.14,-2.04)	-2.26 (-2.35,-2.17)
Tunisia	-0.41 (-0.49,-0.33)	-0.7 (-0.85,-0.55)	-0.30 (-0.37,-0.24)	-0.56 (-0.68,-0.43)

Turkey	-1.32 (-1.45,-1.18)	-1.63 (-1.88,-1.38)	-1.34 (-1.46,-1.22)	-1.64 (-1.87,-1.41)
Turkmenistan	-1.39 (-1.54,-1.25)	-1.92 (-2.19,-1.65)	-1.39 (-1.54,-1.24)	-1.94 (-2.22,-1.66)
Tuvalu	0.15 (0.12,0.18)	0.23 (0.18,0.29)	0.13 (0.11,0.15)	0.22 (0.18,0.26)
Uganda	-0.65 (-0.84,-0.46)	-1.4 (-1.75,-1.04)	-0.62 (-0.82,-0.42)	-1.43 (-1.8,-1.05)
Ukraine	-0.57 (-0.81,-0.33)	-0.9 (-1.35,-0.45)	-0.46 (-0.69,-0.22)	-0.82 (-1.27,-0.37)
United Arab Emirates	-1.07 (-1.38,-0.76)	0.23 (-0.36,0.83)	-1.66 (-1.96,-1.36)	-0.7 (-1.27,-0.13)
United Kingdom	-2.68 (-2.77,-2.60)	-2.75 (-2.91,-2.59)	-3.00 (-3.07,-2.92)	-3.06 (-3.2,-2.92)
United Republic of Tanzania	-1.56 (-1.65,-1.47)	-2.06 (-2.22,-1.89)	-1.55 (-1.63,-1.46)	-2.04 (-2.2,-1.88)
United States of America	-0.97 (-1.03,-0.91)	-0.93 (-1.05,-0.81)	-1.15 (-1.23,-1.07)	-1.13 (-1.28,-0.98)
United States Virgin Islands	-1.75 (-1.91,-1.59)	-1.87 (-2.17,-1.57)	-1.85 (-2.00,-1.71)	-1.93 (-2.2,-1.66)

Uruguay	-0.83 (-0.90,-0.75)	-1.18 (-1.32,-1.04)	-1.00 (-1.09,-0.92)	-1.41 (-1.56,-1.25)
Uzbekistan	1.44 (1.25,1.64)	1.54 (1.16,1.92)	1.18 (0.96,1.39)	1.22 (0.81,1.63)
Vanuatu	-0.72 (-0.78,-0.67)	-0.89 (-0.99,-0.79)	-0.66 (-0.71,-0.61)	-0.84 (-0.93,-0.74)
Venezuela (Bolivarian Republic of)	-1.34 (-1.44,-1.25)	-1.74 (-1.92,-1.56)	-1.44 (-1.54,-1.34)	-1.86 (-2.05,-1.68)
Viet Nam	0.17 (0.11,0.24)	-0.1 (-0.22,0.02)	0.28 (0.23,0.33)	0.11 (0.01,0.2)
Western Europe	-2.21 (-2.24,-2.19)	-2.27 (-2.31,-2.23)	-2.32 (-2.33,-2.30)	-2.34 (-2.37,-2.31)
Western Sub-Saharan Africa	-0.16 (-0.19,-0.13)	-0.18 (-0.24,-0.12)	-0.21 (-0.25,-0.17)	-0.27 (-0.34,-0.19)
Yemen	0.13 (0.10,0.16)	0.11 (0.06,0.16)	-0.13 (-0.16,-0.10)	-0.18 (-0.23,-0.13)
Zambia	-0.09 (-0.15,-0.03)	-0.23 (-0.35,-0.11)	-0.03 (-0.10,0.03)	-0.22 (-0.35,-0.1)
Zimbabwe	-0.01 (-0.16,0.13)	0.19 (-0.09,0.47)	0.13 (-0.02,0.28)	0.2 (-0.09,0.5)

AAPC, average annual percentage change; EAPC, estimated annual percentage change; ASMR, age-standardized mortality rates; ASDR, age-standardized disability-adjusted life year rates; CI, confidence interval.

Supplementary Table 2. The global burden of bladder cancer attributable to smoking in 2021.

location_name	Deaths cases (95% UI)	ASMR (95% UI)	PAF of ASM (95% UI)	DALYs cases (95% UI)	ASDR (95% UI)	PAF of ADM (95% UI)
Afghanistan	38 (22,58)	0.48 (0.28,0.71)	13.06 (9.44,16.93)	995 (544,1528)	10.48 (5.9,15.88)	13.52 (9.5,17.55)
Albania	5 (4,8)	0.12 (0.08,0.18)	39.91 (33.82,45.94)	105 (72,150)	2.33 (1.59,3.33)	40.39 (34.77,46.09)
Algeria	140 (99,194)	0.55 (0.38,0.75)	30.58 (25.15,36.46)	2896 (2047,3959)	9.38 (6.6,12.92)	30.66 (25.27,35.78)
American Samoa	0 (0,0)	0.37 (0.28,0.48)	18.89 (15.3,23.2)	5 (3,6)	9.07 (6.89,11.79)	20.62 (16.7,24.77)
Andean Latin America	88 (64,119)	0.16 (0.11,0.21)	10.24 (8.16,12.33)	1746 (1275,2360)	3.01 (2.2,4.09)	10.25 (8.34,12.27)
Andorra	2 (1,3)	1.14 (0.71,1.65)	29.67 (23.78,35.55)	37 (23,55)	23.98 (14.91,35.41)	32.11 (26.02,38.14)
Angola	36 (25,50)	0.37 (0.26,0.53)	14.6 (11.83,17.68)	947 (675,1334)	8.19 (5.84,11.45)	15.54 (12.71,18.59)
Antigua and Barbuda	0 (0,0)	0.35 (0.27,0.44)	12.64 (9.67,15.92)	8 (6,10)	7.15 (5.48,9)	13.66 (10.61,16.88)
Argentina	365 (287,447)	0.64 (0.51,0.78)	21.65 (17.92,25.87)	8426 (6807,10155)	15.18 (12.3,18.15)	25.13 (21.15,29.45)
Armenia	101 (82,125)	2.3 (1.85,2.83)	44.77 (40.29,49.26)	2238 (1807,2710)	50.89 (41.12,61.61)	45.28 (40.82,49.57)

Australasia	235 (178,305)	0.41 (0.31,0.52)	14.63 (11.4,18.32)	4531 (3608,5735)	8.45 (6.8,10.61)	17.05 (13.82,20.96)
Australia	177 (133,235)	0.36 (0.28,0.48)	13.43 (10.39,17.08)	3468 (2750,4446)	7.75 (6.22,9.76)	16.08 (13.07,19.92)
Austria	159 (126,199)	0.81 (0.65,1)	25.24 (20.99,29.45)	3232 (2648,3888)	18.23 (15.07,21.75)	29.36 (24.93,33.72)
Azerbaijan	65 (45,92)	0.69 (0.48,0.96)	39.3 (34.29,44.39)	1712 (1140,2454)	16.06 (10.83,22.64)	39.5 (34.63,44.41)
Bahamas	1 (1,1)	0.25 (0.18,0.32)	13.57 (10.25,17.04)	22 (15,28)	5.37 (3.81,6.9)	14.29 (11.07,17.75)
Bahrain	8 (5,12)	1.38 (0.93,1.96)	26.88 (22.21,32.02)	216 (139,326)	27.39 (18.03,39.44)	28.28 (23.66,33.38)
Bangladesh	422 (239,865)	0.35 (0.21,0.73)	30.37 (25.6,35.63)	8479 (4757,17457)	6.49 (3.67,13.34)	29.17 (24.79,34.57)
Barbados	2 (1,2)	0.31 (0.21,0.42)	11.84 (9.3,14.72)	30 (21,41)	5.64 (3.89,7.71)	11.6 (9.22,14.26)
Belarus	162 (127,198)	0.98 (0.77,1.2)	39.56 (34.72,44.05)	3808 (2967,4662)	23.17 (17.95,28.4)	41.56 (36.63,46.19)
Belgium	267 (214,328)	1.05 (0.86,1.27)	25.3 (21.15,30.07)	5179 (4339,6200)	22.52 (19.05,26.78)	28.34 (24.12,33.06)
Belize	1 (1,1)	0.29 (0.21,0.36)	14.43 (11.66,17.32)	17 (13,22)	5.98 (4.55,7.52)	14.63 (12.04,17.4)
Benin	5 (3,7)	0.1 (0.07,0.15)	5.58 (4.34,6.87)	114 (78,158)	2.25 (1.55,3.18)	6.05 (4.76,7.4)

Bermuda	1 (1,2)	0.78 (0.58,1.02)	17.29 (13.46,21.74)	23 (17,30)	16.34 (12.16,21.35)	18.88 (15.25,23.02)
Bhutan	1 (1,2)	0.19 (0.11,0.44)	15.96 (11.71,20.9)	19 (11,44)	3.37 (1.91,7.72)	14.95 (10.97,19.69)
Bolivia (Plurinational State of)	21 (13,30)	0.25 (0.16,0.37)	11.24 (8.76,13.95)	446 (297,654)	5.05 (3.33,7.45)	11.64 (9.29,14.31)
Bosnia and Herzegovina	101 (73,135)	1.55 (1.11,2.06)	37.48 (32.26,42.2)	2157 (1552,2864)	33.61 (24.1,44.64)	39.29 (34.31,44.15)
Botswana	5 (4,7)	0.42 (0.31,0.58)	20.4 (16.99,24.13)	124 (87,180)	8.85 (6.28,12.43)	20.15 (16.86,23.99)
Brazil	1235 (999,1524)	0.5 (0.41,0.62)	21.02 (17.16,25.45)	26059 (21410,31667)	10.37 (8.51,12.63)	21.77 (18.02,25.94)
Brunei Darussalam	1 (1,2)	0.53 (0.39,0.69)	21 (16.6,25.15)	35 (25,45)	10.59 (7.78,13.63)	23.02 (18.64,27.37)
Bulgaria	207 (164,261)	1.41 (1.13,1.79)	27.32 (23.27,31.72)	5024 (3973,6342)	36.7 (28.81,46.68)	32.95 (28.49,37.56)
Burkina Faso	7 (5,10)	0.08 (0.06,0.12)	4.45 (3.36,5.69)	191 (134,269)	1.97 (1.39,2.79)	5.1 (3.94,6.34)
Burundi	7 (4,10)	0.17 (0.11,0.25)	6.8 (5.29,8.52)	169 (107,256)	3.61 (2.36,5.36)	7.18 (5.71,8.82)
Cabo Verde	1 (0,1)	0.16 (0.08,0.24)	7.83 (6.23,9.79)	16 (8,24)	3.65 (1.81,5.31)	8.57 (6.91,10.57)
Cambodia	56 (38,86)	0.54 (0.37,0.83)	38.79 (33.03,45.27)	1315 (897,1967)	11.06 (7.54,16.76)	37.25 (31.89,43.19)

Cameroon	18 (12,26)	0.16 (0.11,0.23)	6.61 (5.16,8.39)	510 (336,722)	3.79 (2.5,5.41)	7.54 (5.95,9.44)
Canada	596 (459,751)	0.76 (0.59,0.94)	22.43 (18.16,27.41)	10915 (8736,13458)	14.78 (11.93,18.12)	24.52 (20.4,29.44)
Caribbean	318 (253,391)	0.59 (0.47,0.72)	20.87 (17.35,24.58)	6682 (5387,8119)	12.35 (9.94,14.99)	22.05 (18.6,25.55)
Central African Republic	4 (3,6)	0.23 (0.15,0.32)	8.23 (6.35,10.37)	120 (78,175)	5.28 (3.6,7.63)	8.82 (6.88,10.98)
Central Asia	481 (405,570)	0.65 (0.55,0.77)	32.19 (28.44,36.35)	11977 (10048,14054)	14.57 (12.24,17.19)	32.84 (29.17,36.87)
Central Europe	3488 (2950,4118)	1.5 (1.27,1.77)	27.82 (24.06,31.87)	77675 (66589,91156)	35.09 (30.16,41.06)	31.32 (27.43,35.32)
Central Europe, Eastern Europe, and Central Asia	7235 (6199,8360)	1.08 (0.93,1.25)	29.3 (25.53,33.28)	168579 (145321,192711)	25.42 (21.97,29.05)	32.54 (28.65,36.65)
Central Latin America	425 (339,519)	0.18 (0.14,0.22)	12.86 (10.82,15.27)	8906 (7213,10840)	3.6 (2.9,4.37)	12.73 (10.78,14.98)
Central Sub-Saharan Africa	100 (72,135)	0.22 (0.16,0.29)	8.17 (6.79,9.81)	2666 (1933,3619)	4.86 (3.52,6.58)	8.85 (7.43,10.47)
Chad	9 (6,14)	0.19 (0.12,0.29)	8.69 (6.47,11.34)	222 (135,327)	3.99 (2.44,5.9)	8.75 (6.64,11.23)
Chile	80 (62,100)	0.31 (0.24,0.39)	12.82 (10.1,16.02)	1922 (1561,2337)	7.48 (6.09,9.08)	15.91 (13.05,18.94)
China	17178 (12936,23471)	0.87 (0.66,1.18)	37.05 (32.26,42.19)	359985 (266681,490838)	17.09 (12.74,23.35)	37.62 (33.05,42.11)

Colombia	77 (58,103)	0.14 (0.1,0.19)	9.91 (7.91,12.29)	1608 (1216,2139)	2.92 (2.21,3.88)	10.26 (8.35,12.57)
Comoros	1 (1,2)	0.28 (0.15,0.49)	12.31 (8.18,17.17)	24 (12,40)	5.27 (2.75,9.15)	11.37 (7.49,15.75)
Congo	8 (6,12)	0.41 (0.28,0.58)	13.09 (10.28,16.16)	206 (139,299)	8.32 (5.68,12.14)	13.1 (10.55,15.84)
Cook Islands	0 (0,0)	0.48 (0.35,0.65)	16.14 (12.62,20.33)	3 (2,4)	11.63 (8.47,16.1)	17.58 (13.98,21.9)
Costa Rica	19 (15,25)	0.35 (0.27,0.45)	18.28 (14.85,22.29)	391 (306,487)	7.14 (5.58,8.9)	18.24 (15.09,21.89)
Croatia	176 (139,218)	1.81 (1.43,2.23)	33.95 (29.3,39.36)	3469 (2765,4280)	38.24 (30.53,47.09)	35.74 (31.13,40.77)
Cuba	211 (164,259)	1.04 (0.81,1.28)	25.9 (21.61,30.26)	4428 (3489,5376)	22.44 (17.79,27.18)	28.31 (24.02,32.71)
Cyprus	27 (20,38)	1.28 (0.92,1.78)	30.08 (25.05,35.38)	561 (405,771)	26.45 (19.15,36.25)	34.19 (29.13,39.25)
Czechia	301 (234,381)	1.31 (1.01,1.65)	27.52 (23.29,31.91)	6504 (5092,8275)	29.89 (23.32,37.75)	30.77 (26.45,35.49)
C 么 te d'Ivoire	20 (13,29)	0.22 (0.15,0.32)	9.12 (6.94,11.38)	504 (322,766)	4.7 (3.1,6.82)	9.54 (7.43,11.65)
Democratic People's Republic of Korea	155 (111,230)	0.48 (0.34,0.71)	27.2 (22.55,32.83)	4037 (2834,6009)	11.92 (8.35,17.65)	29.14 (24.59,33.93)
Democratic Republic of the Congo	47 (31,69)	0.15 (0.1,0.22)	5.67 (4.52,7.08)	1289 (855,1879)	3.43 (2.25,5.02)	6.32 (5.07,7.75)

Denmark	195 (160,233)	1.48 (1.23,1.77)	32.26 (27.14,37.58)	3631 (3056,4314)	29.57 (24.82,34.93)	33.81 (28.78,38.85)
Djibouti	2 (1,4)	0.53 (0.32,0.87)	18.85 (13.88,24.1)	56 (32,95)	10.13 (5.92,16.72)	17.98 (13.13,23.03)
Dominica	0 (0,0)	0.36 (0.25,0.52)	10.26 (7.85,13.24)	7 (5,9)	7.73 (5.36,11.03)	11.26 (8.75,14.28)
Dominican Republic	28 (20,41)	0.3 (0.2,0.42)	25.76 (21,30.69)	568 (386,822)	5.79 (3.95,8.35)	24.86 (20.82,29.56)
East Asia	17715 (13442,24011)	0.86 (0.66,1.16)	36.65 (31.98,41.77)	371442 (278522,503619)	17.03 (12.86,23.12)	37.23 (32.73,41.66)
Eastern Europe	3267 (2706,3800)	0.9 (0.75,1.05)	31.52 (27.6,35.67)	78927 (65921,92032)	22.12 (18.51,25.79)	34.63 (30.6,38.75)
Eastern Sub-Saharan Africa	312 (241,415)	0.23 (0.18,0.3)	8.59 (6.98,10.17)	7380 (5571,9987)	4.69 (3.61,6.27)	8.63 (7.03,10.18)
Ecuador	27 (19,36)	0.17 (0.12,0.23)	11.27 (8.8,14.16)	520 (365,695)	3.23 (2.28,4.33)	11.37 (9.13,13.84)
Egypt	803 (531,1428)	1.6 (1.03,2.94)	36.12 (30.85,41.36)	20753 (13988,36281)	34.21 (22.78,61.01)	37.05 (32.24,41.74)
El Salvador	5 (4,7)	0.09 (0.06,0.12)	9.09 (7.11,11.59)	123 (88,171)	2.03 (1.45,2.81)	10.07 (8.09,12.68)
Equatorial Guinea	1 (1,2)	0.28 (0.17,0.46)	9.65 (6.96,13.23)	30 (18,47)	6.02 (3.64,9.76)	10.37 (7.58,14.09)
Eritrea	3 (2,5)	0.14 (0.09,0.22)	4.78 (3.39,6.49)	96 (58,151)	3.25 (2.06,5.03)	5.34 (3.79,7.31)

Estonia	25 (20,31)	0.89 (0.72,1.09)	24.39 (20.41,28.94)	543 (438,658)	21.02 (17.04,25.4)	28.68 (24.38,33.1)
Eswatini	1 (1,2)	0.22 (0.14,0.32)	8.19 (6.38,10.38)	26 (17,39)	4.84 (3.1,7.18)	7.62 (6.04,9.29)
Ethiopia	36 (21,60)	0.1 (0.06,0.16)	3.88 (2.94,5.1)	805 (477,1375)	1.98 (1.18,3.35)	4.04 (3.02,5.28)
Fiji	2 (1,3)	0.25 (0.14,0.35)	14.36 (11.61,17.36)	51 (28,73)	6.09 (3.44,8.77)	15.78 (12.87,18.7)
Finland	55 (43,70)	0.4 (0.31,0.5)	17.58 (14.05,21.7)	1114 (875,1357)	9.03 (7.25,10.97)	20.63 (16.95,24.76)
France	1669 (1311,2071)	1.09 (0.87,1.32)	23.72 (19.61,28.32)	32202 (26129,38715)	23.73 (19.17,28.34)	26.67 (22.41,31)
Gabon	3 (2,4)	0.32 (0.22,0.48)	9.47 (7.47,11.83)	75 (51,110)	7.12 (4.99,10.47)	10.23 (8.17,12.65)
Gambia	1 (1,2)	0.14 (0.1,0.21)	9.76 (7.61,12.24)	30 (20,42)	3.11 (2.12,4.48)	10.47 (8.27,12.99)
Georgia	119 (97,143)	1.96 (1.58,2.37)	38.31 (34.23,43.05)	2707 (2209,3269)	46.02 (37.52,55.66)	39.82 (35.72,44.22)
Germany	1903 (1492,2333)	0.89 (0.71,1.08)	25.21 (20.78,29.71)	36350 (29465,44147)	19.16 (15.97,23.11)	28.24 (23.61,32.58)
Ghana	23 (15,35)	0.19 (0.12,0.28)	7.89 (6.06,10.17)	529 (360,784)	3.45 (2.32,5.21)	6.86 (5.28,8.75)
Global	58767 (49381,70892)	0.7 (0.59,0.84)	26.02 (22.36,29.97)	1238303 (1044303,1478221)	14.33 (12.09,17.14)	27.78 (24.06,31.56)

Greece	598 (495,712)	2.21 (1.86,2.61)	37.02 (31.76,42.16)	10950 (9282,12899)	46.88 (40.34,54.59)	40.4 (35.29,45.43)
Greenland	1 (0,1)	1.07 (0.72,1.47)	30.21 (25.24,35.5)	17 (11,24)	23.56 (15.97,32.81)	33.17 (27.93,38.48)
Grenada	0 (0,0)	0.3 (0.23,0.39)	10.9 (8.43,13.7)	8 (6,10)	6.75 (5.04,8.54)	11.91 (9.4,14.67)
Guam	0 (0,1)	0.23 (0.17,0.29)	19.17 (15.59,23.52)	14 (10,18)	6.58 (4.91,8.4)	20.38 (16.62,25.07)
Guatemala	7 (6,10)	0.07 (0.05,0.09)	9.48 (7.59,11.71)	159 (120,202)	1.48 (1.11,1.88)	9.3 (7.53,11.18)
Guinea	19 (12,28)	0.39 (0.25,0.57)	11.04 (8.55,13.63)	435 (283,651)	8.05 (5.25,11.98)	11.47 (8.86,14.09)
Guinea-Bissau	1 (1,2)	0.16 (0.1,0.24)	6.06 (4.58,7.79)	29 (19,44)	3.76 (2.47,5.66)	6.69 (5.17,8.41)
Guyana	1 (1,2)	0.21 (0.15,0.29)	12.38 (9.86,15.32)	32 (22,44)	4.81 (3.3,6.66)	13.18 (10.6,15.85)
Haiti	11 (7,17)	0.19 (0.11,0.29)	6.4 (4.73,8.56)	283 (168,413)	4.07 (2.45,6)	6.76 (5.16,8.66)
High SDI	19185 (15485,23062)	0.84 (0.69,1)	24.67 (20.79,29.08)	375066 (313626,439688)	17.81 (15.02,20.78)	27.38 (23.43,31.62)
High-income	20546 (16716,24948)	0.85 (0.7,1.02)	24.24 (20.36,28.65)	397276 (330512,473434)	18.1 (15.26,21.31)	27.09 (23.09,31.35)
High-income Asia Pacific	3038 (2407,3679)	0.53 (0.43,0.63)	24.4 (20.51,28.44)	51134 (42105,60315)	10.56 (8.79,12.27)	26.9 (23.02,30.92)

High-income North America	5554 (4464,6911)	0.8 (0.64,0.99)	23.41 (19.24,28.36)	118017 (96876,142893)	17.77 (14.64,21.36)	26.4 (22.25,31.05)
High-middle SDI	19650 (16416,23607)	0.99 (0.82,1.19)	30.33 (26.16,34.67)	423434 (356414,505774)	21.06 (17.71,25.17)	32.97 (28.73,37.07)
Honduras	7 (5,11)	0.13 (0.08,0.2)	18.18 (14.28,22.17)	165 (104,253)	2.69 (1.68,4.16)	18.41 (14.52,22.18)
Hungary	282 (225,354)	1.42 (1.14,1.78)	27.14 (22.9,31.47)	6760 (5429,8447)	36.36 (29.27,44.73)	30.96 (26.46,35.55)
Iceland	4 (3,6)	0.72 (0.56,0.91)	21.99 (17.53,26.97)	88 (70,113)	15.09 (12.04,19.24)	24.98 (20.62,29.65)
India	2494 (1952,3204)	0.24 (0.19,0.3)	19.45 (16.35,22.66)	53665 (41655,69142)	4.7 (3.65,6.05)	18.81 (16.1,21.84)
Indonesia	668 (419,1193)	0.35 (0.21,0.63)	28.86 (24.6,33.72)	16260 (10245,28369)	7.05 (4.44,12.55)	28.5 (24.44,33)
Iran (Islamic Republic of)	340 (252,423)	0.48 (0.35,0.6)	21.45 (17.55,25.3)	8311 (6262,10275)	10.77 (8.03,13.39)	23.59 (19.83,27.61)
Iraq	347 (227,502)	1.84 (1.22,2.63)	33.75 (28.02,39.16)	8248 (5397,12180)	37.44 (24.61,54.54)	33.85 (28.63,39)
Ireland	60 (47,75)	0.71 (0.56,0.89)	23.52 (19.37,27.97)	1135 (894,1396)	13.88 (10.98,17.02)	24.95 (20.85,29.1)
Israel	112 (87,141)	0.86 (0.67,1.07)	22.38 (18.22,27.15)	2192 (1742,2678)	17.74 (14.21,21.61)	25.49 (21.19,30.06)
Italy	1943 (1541,2357)	1.18 (0.96,1.41)	24.4 (20.21,28.71)	37094 (30037,44360)	25.58 (21.17,30.13)	27.62 (23.48,31.91)

Jamaica	12 (9,17)	0.4 (0.28,0.56)	15.99 (12.91,19.7)	268 (187,371)	8.73 (6.09,12.13)	17.02 (13.85,20.68)
Japan	2376 (1846,2937)	0.52 (0.42,0.62)	23.34 (19.48,27.36)	39094 (31528,46856)	10.59 (8.87,12.46)	26.11 (22.34,30.08)
Jordan	60 (42,85)	1.01 (0.71,1.41)	38.81 (33.3,43.89)	1545 (1058,2190)	21.37 (14.96,30.18)	40.28 (35.03,45.33)
Kazakhstan	88 (69,109)	0.5 (0.39,0.62)	25.31 (21.67,29.07)	2390 (1876,2931)	12.55 (9.82,15.48)	27.8 (24.05,31.42)
Kenya	21 (15,27)	0.11 (0.08,0.15)	8.92 (6.89,11)	487 (356,641)	2.28 (1.65,2.96)	9.01 (7,11.05)
Kiribati	0 (0,0)	0.15 (0.11,0.19)	26.03 (21.87,30.66)	3 (2,4)	3.66 (2.7,4.74)	27.31 (23.47,31.69)
Kuwait	18 (14,23)	0.8 (0.6,1.03)	26.9 (22.11,32.15)	461 (349,590)	16.66 (12.42,21.5)	28 (23.27,32.96)
Kyrgyzstan	24 (18,30)	0.52 (0.4,0.67)	38.12 (33.36,42.58)	637 (493,815)	12.61 (9.78,16.09)	38.37 (34.08,42.7)
Lao People's Democratic Republic	18 (11,27)	0.47 (0.3,0.73)	35.76 (29.79,41.45)	409 (263,620)	9.42 (6.05,14.44)	34.26 (28.67,39.52)
Latin America and Caribbean	2089 (1705,2531)	0.35 (0.28,0.42)	17.93 (14.84,21.51)	43847 (36334,52519)	7.11 (5.88,8.52)	18.37 (15.42,21.66)
Latvia	54 (43,66)	1.32 (1.05,1.62)	28.38 (24.15,32.7)	1189 (945,1462)	31.45 (25.3,38.6)	31.98 (27.52,36.03)
Lebanon	189 (136,259)	2.94 (2.12,4.01)	37.54 (32,43)	3592 (2622,4812)	58.87 (43.13,78.57)	39.83 (34.57,45.03)

Lesotho	5 (3,8)	0.56 (0.34,0.83)	23.86 (19.08,28.68)	139 (78,212)	12.73 (7.4,19.2)	23.45 (19.04,28)
Liberia	2 (1,3)	0.12 (0.07,0.17)	5.57 (4.36,6.97)	59 (38,90)	2.64 (1.71,3.87)	6.3 (5.05,7.72)
Libya	86 (59,124)	1.97 (1.36,2.89)	31.2 (26.15,36.03)	2045 (1413,2934)	41 (28.28,58.79)	32.15 (27.37,36.39)
Lithuania	71 (57,86)	1.14 (0.93,1.38)	27.69 (23.62,31.91)	1412 (1158,1689)	24.79 (20.3,29.62)	29.67 (25.66,33.99)
Low SDI	1127 (911,1421)	0.27 (0.22,0.35)	12.13 (10.22,14.01)	26248 (21113,32827)	5.53 (4.47,6.94)	12.03 (10.21,13.92)
Low-middle SDI	5103 (4109,7285)	0.4 (0.32,0.57)	23.67 (20.37,27.32)	116525 (94039,165601)	8.33 (6.73,11.87)	23.6 (20.49,27.07)
Luxembourg	10 (8,13)	0.93 (0.73,1.17)	23.99 (19.17,29.15)	208 (163,257)	19.71 (15.53,24.26)	27.39 (22.21,32.61)
Madagascar	9 (6,13)	0.12 (0.08,0.18)	6.15 (4.77,7.8)	231 (148,324)	2.34 (1.54,3.37)	5.66 (4.44,7.02)
Malawi	72 (49,98)	1.21 (0.85,1.66)	14.11 (10.32,18)	1671 (1104,2340)	24.5 (16.71,33.56)	13.64 (9.8,17.39)
Malaysia	175 (116,239)	0.72 (0.47,0.98)	26.87 (21.83,31.56)	3698 (2511,4942)	13.73 (9.18,18.46)	26.25 (21.71,30.72)
Maldives	1 (1,2)	0.46 (0.34,0.6)	41.81 (36.38,47.3)	22 (16,30)	7.7 (5.65,10.15)	39.62 (34.5,44.52)
Mali	67 (46,95)	1.04 (0.71,1.49)	11.52 (9.07,14.43)	1460 (1017,2109)	18.99 (13.12,27.13)	10.72 (8.6,13.23)

Malta	8 (6,10)	0.79 (0.62,0.99)	21 (17.12,25.57)	174 (140,218)	18.81 (15.26,23.45)	25.37 (21.29,30.18)
Marshall Islands	0 (0,0)	0.37 (0.23,0.53)	17.98 (14.02,22.75)	3 (2,5)	8.78 (5.55,12.8)	18.86 (14.78,24.14)
Mauritania	3 (2,5)	0.16 (0.09,0.26)	7.95 (6.09,10.11)	73 (42,119)	3.38 (1.94,5.52)	8.7 (6.65,10.85)
Mauritius	10 (9,12)	0.57 (0.48,0.67)	25.49 (21.91,29.6)	224 (193,260)	11.99 (10.3,13.94)	25.14 (21.97,28.72)
Mexico	225 (175,278)	0.19 (0.15,0.24)	13.74 (11.29,16.43)	4642 (3686,5753)	3.74 (2.96,4.64)	13.17 (11.11,15.47)
Micronesia (Federated States of)	0 (0,0)	0.44 (0.29,0.62)	22.91 (18.59,27.23)	10 (6,13)	11.38 (7.45,15.89)	24.88 (20.52,29.57)
Middle SDI	13623 (10820,17705)	0.55 (0.44,0.71)	29.16 (25.19,33.65)	295302 (233937,385222)	11.12 (8.83,14.45)	29.49 (25.51,33.73)
Monaco	1 (1,4)	1.37 (0.61,3.39)	24.4 (17.92,30.7)	28 (13,70)	29.09 (12.98,73.52)	27.49 (20.77,33.95)
Mongolia	6 (5,9)	0.3 (0.21,0.43)	24.74 (20.5,29.8)	178 (126,256)	7.3 (5.19,10.47)	25.72 (21.49,30.75)
Montenegro	15 (11,20)	1.49 (1.07,2.03)	31.98 (27.18,36.98)	347 (256,468)	33.94 (25.02,45.59)	36.94 (31.99,42.39)
Morocco	81 (55,115)	0.25 (0.17,0.36)	17.18 (13.77,21.23)	1943 (1313,2739)	5.62 (3.84,8.01)	19.71 (16.21,23.77)
Mozambique	24 (17,34)	0.27 (0.19,0.38)	8.74 (6.78,10.86)	584 (422,828)	5.58 (4.03,7.93)	8.77 (6.96,10.68)

Myanmar	122 (83,199)	0.29 (0.2,0.49)	25.4 (21.67,29.59)	2654 (1818,4181)	5.73 (3.93,9.2)	23.42 (20.07,26.91)
Namibia	3 (2,4)	0.27 (0.21,0.36)	16.04 (13.33,19.4)	61 (45,80)	5.04 (3.82,6.62)	14.13 (11.94,17.05)
Nauru	0 (0,0)	0.48 (0.29,0.7)	20.51 (16.7,24.89)	1 (0,1)	12.35 (7.25,18.15)	21.92 (17.92,26.02)
Nepal	56 (36,110)	0.29 (0.19,0.57)	23.52 (19.67,27.39)	1105 (703,2221)	5.18 (3.33,10.34)	21.72 (18.45,25.07)
Netherlands	449 (368,552)	1.16 (0.95,1.42)	27.64 (22.77,33.12)	8096 (6725,9891)	22.14 (18.47,26.84)	29.15 (24.61,34.08)
New Zealand	58 (45,74)	0.64 (0.49,0.81)	20.45 (16.37,25.34)	1064 (838,1320)	12.27 (9.77,15.16)	21.56 (17.71,26.18)
Nicaragua	4 (3,5)	0.09 (0.06,0.12)	14.01 (11.05,17.68)	85 (62,116)	1.78 (1.3,2.42)	13.9 (11.2,17.38)
Niger	4 (3,8)	0.08 (0.05,0.15)	5.76 (4.01,7.72)	96 (58,166)	1.41 (0.83,2.46)	4.93 (3.57,6.41)
Nigeria	17 (11,27)	0.02 (0.01,0.04)	2.88 (2.11,3.86)	398 (268,625)	0.45 (0.3,0.73)	3.19 (2.36,4.22)
Niue	0 (0,0)	0.33 (0.22,0.47)	18.89 (14.85,23.74)	0 (0,0)	8.27 (5.38,11.75)	20.72 (16.21,26.06)
North Africa and Middle East	3820 (3032,4991)	0.97 (0.77,1.26)	29.97 (25.83,33.98)	92413 (73519,121067)	20.98 (16.63,27.44)	31.6 (27.61,35.54)
North Africa and Middle East	3820 (3032,4991)	0.97 (0.77,1.26)	29.97 (25.83,33.98)	92413 (73519,121067)	20.98 (16.63,27.44)	31.6 (27.61,35.54)

North Macedonia	56 (40,74)	1.75 (1.28,2.29)	34.02 (29.23,38.67)	1302 (936,1709)	37.94 (27.47,49.84)	37.57 (32.47,42.25)
Northern Mariana Islands	0 (0,0)	0.49 (0.38,0.64)	14.93 (11.89,18.96)	7 (5,9)	11.87 (9.14,15.31)	16.56 (13.4,20.86)
Norway	73 (55,92)	0.66 (0.5,0.82)	17.53 (14.21,21.42)	1331 (1071,1617)	13 (10.54,15.71)	19.48 (16.21,23.1)
Oceania	15 (9,20)	0.21 (0.13,0.29)	15.18 (12.48,18.09)	423 (260,601)	5.19 (3.22,7.28)	16.53 (13.6,19.73)
Oman	3 (2,4)	0.22 (0.15,0.3)	14.58 (11.48,17.88)	83 (58,112)	4.56 (3.13,6.22)	15.17 (12.3,18.22)
Pakistan	1217 (858,1782)	1.25 (0.87,1.84)	24.18 (19.89,28.75)	27682 (19440,40572)	24.89 (17.55,36.4)	23.98 (19.84,28.25)
Palau	0 (0,0)	0.12 (0.09,0.16)	20.7 (16.75,25.86)	1 (1,1)	2.97 (2.09,4.12)	21.09 (17.1,25.91)
Palestine	23 (17,31)	1.11 (0.83,1.53)	34.13 (28.97,39.18)	580 (426,761)	23.78 (17.7,31.82)	35.56 (30.37,40.49)
Panama	6 (5,9)	0.14 (0.1,0.19)	12.66 (10.04,15.69)	127 (92,168)	2.88 (2.09,3.83)	12.62 (10.39,15.25)
Papua New Guinea	8 (4,12)	0.16 (0.09,0.26)	12.71 (9.78,16.27)	238 (127,390)	4.17 (2.27,6.6)	14.27 (10.93,18.14)
Paraguay	23 (16,32)	0.43 (0.3,0.6)	29.55 (24.49,34.77)	455 (310,638)	8.16 (5.6,11.41)	28.35 (23.81,33.25)
Peru	40 (25,61)	0.12 (0.08,0.19)	9.11 (7.1,11.4)	779 (495,1189)	2.36 (1.49,3.61)	8.94 (7.07,11.07)

Philippines	189 (144,271)	0.25 (0.19,0.37)	27.88 (23.72,32.32)	4814 (3606,6702)	5.77 (4.37,8.21)	27.91 (23.9,32.04)
Poland	1398 (1142,1681)	1.85 (1.52,2.22)	27.15 (23.03,31.49)	30413 (25065,36248)	41.86 (34.61,49.81)	30.55 (26.55,35.12)
Portugal	189 (147,235)	0.74 (0.59,0.9)	18.02 (14.7,21.8)	4089 (3261,4973)	17.96 (14.6,21.7)	21.91 (18.33,25.91)
Puerto Rico	28 (20,37)	0.35 (0.26,0.47)	16.37 (12.45,21.01)	541 (392,726)	7.75 (5.66,10.4)	17.4 (13.51,22.1)
Qatar	3 (2,5)	0.55 (0.35,0.86)	16.61 (12.93,20.71)	113 (68,174)	12.1 (7.49,18.82)	18.97 (14.9,23.33)
Republic of Korea	644 (448,851)	0.69 (0.48,0.9)	32.47 (26.97,38.06)	11638 (8309,15106)	12.28 (8.78,15.93)	33 (27.68,38.17)
Republic of Moldova	61 (51,72)	1 (0.82,1.17)	32.76 (27.97,37.99)	1565 (1299,1832)	25.57 (21.27,29.76)	36.21 (31.36,41.63)
Romania	500 (397,612)	1.31 (1.05,1.59)	27.09 (23.12,31.63)	11539 (9337,13962)	32.1 (26.09,38.68)	30.42 (26.26,35.13)
Russian Federation	2197 (1816,2588)	0.89 (0.74,1.05)	31.56 (27.82,35.69)	53476 (44416,62662)	21.89 (18.23,25.6)	35.07 (31.13,39.35)
Rwanda	27 (18,42)	0.57 (0.38,0.88)	23.08 (18.36,28.26)	587 (377,930)	10.64 (7.04,16.54)	21.5 (17.13,26.21)
Saint Kitts and Nevis	0 (0,0)	0.3 (0.22,0.42)	9.47 (7.06,12.51)	4 (3,6)	6.02 (4.31,8.2)	10.04 (7.79,13.02)
Saint Lucia	1 (1,1)	0.4 (0.29,0.53)	12.53 (9.93,15.96)	20 (15,26)	8.24 (6.1,10.9)	13.65 (11.17,16.84)

Saint Vincent and the Grenadines	0 (0,1)	0.33 (0.25,0.42)	12.36 (9.72,15.28)	10 (8,13)	6.91 (5.38,8.77)	13.32 (10.75,16.13)
Samoa	1 (0,1)	0.38 (0.26,0.53)	31.2 (26.65,36.15)	12 (8,18)	8.44 (5.73,12.01)	33.44 (28.81,38.18)
San Marino	1 (0,1)	0.91 (0.57,1.36)	23.84 (19.06,28.82)	14 (9,21)	19.44 (11.51,30.06)	26.58 (21.63,31.61)
Sao Tome and Principe	0 (0,0)	0.27 (0.17,0.42)	6.08 (4.61,7.94)	7 (4,11)	6.17 (3.88,9.36)	6.93 (5.33,8.83)
Saudi Arabia	45 (28,83)	0.27 (0.17,0.49)	16.01 (12.58,19.74)	1430 (873,2719)	6.75 (4.2,12.57)	18.73 (14.88,22.74)
Senegal	12 (8,17)	0.16 (0.1,0.24)	6.9 (5.29,8.61)	309 (206,451)	3.81 (2.5,5.67)	8.3 (6.45,10.21)
Serbia	248 (176,336)	1.44 (1.03,1.93)	29.21 (24.54,33.91)	5596 (3983,7549)	33.82 (24.38,45.25)	33.07 (28.4,38.07)
Seychelles	1 (1,1)	1.07 (0.84,1.39)	27.78 (23.72,32.18)	24 (19,32)	21.47 (16.84,28.1)	28.07 (24.37,32.19)
Sierra Leone	5 (4,8)	0.16 (0.11,0.23)	8.14 (6.74,9.9)	134 (93,197)	3.49 (2.46,5.06)	8.91 (7.57,10.67)
Singapore	17 (13,22)	0.2 (0.16,0.26)	14.3 (11.43,17.46)	367 (291,452)	4.21 (3.34,5.19)	16.39 (13.32,19.55)
Slovakia	107 (77,147)	1.08 (0.78,1.47)	26.74 (22.21,31.37)	2416 (1753,3305)	24.76 (18.08,33.52)	29.19 (24.79,33.79)
Slovenia	41 (31,53)	0.9 (0.7,1.15)	20.26 (16.33,25.04)	912 (711,1159)	21.58 (16.81,27.11)	25.13 (20.89,30.05)

Solomon Islands	1 (1,2)	0.42 (0.24,0.63)	24.44 (20.26,28.52)	39 (22,60)	10.5 (5.89,16.05)	26.2 (21.91,30.36)
Somalia	9 (5,15)	0.19 (0.11,0.31)	7.35 (4.49,10.81)	244 (133,411)	4.18 (2.33,6.99)	7.54 (4.61,11.05)
South Africa	148 (116,180)	0.34 (0.27,0.42)	14.94 (12.6,17.69)	3769 (2982,4565)	7.95 (6.29,9.66)	15.94 (13.66,18.62)
South Asia	4190 (3268,5625)	0.33 (0.25,0.44)	21.5 (18.4,24.55)	90950 (70812,121087)	6.43 (4.99,8.6)	20.81 (17.83,23.62)
South Asia	4190 (3268,5625)	0.33 (0.25,0.44)	21.5 (18.4,24.55)	90950 (70812,121087)	6.43 (4.99,8.6)	20.81 (17.83,23.62)
South Sudan	7 (4,11)	0.24 (0.15,0.36)	9.16 (6.66,12.47)	170 (105,271)	4.79 (2.97,7.38)	9.03 (6.59,12.16)
Southeast Asia	2276 (1834,3010)	0.4 (0.32,0.54)	28.8 (25.03,32.13)	51742 (41719,68489)	8.15 (6.56,10.73)	28.46 (24.96,31.85)
Southeast Asia, East Asia, and Oceania	20006 (15591,26507)	0.77 (0.6,1.01)	35.54 (31.06,40.43)	423607 (324483,568837)	15.05 (11.59,20.05)	35.89 (31.45,40.18)
Southern Latin America	518 (415,631)	0.58 (0.47,0.71)	19.83 (16.4,23.63)	11868 (9712,14206)	13.69 (11.24,16.33)	23.21 (19.56,26.84)
Southern Sub-Saharan Africa	246 (201,299)	0.47 (0.37,0.56)	16.25 (13.76,18.79)	6229 (5073,7502)	10.6 (8.62,12.79)	16.76 (14.35,19.34)
Spain	1456 (1159,1804)	1.37 (1.09,1.66)	27.15 (22.4,32.5)	28693 (23198,35050)	30.21 (24.65,36.34)	30.48 (25.84,35.33)
Sri Lanka	42 (24,61)	0.17 (0.1,0.24)	17.6 (14.76,20.71)	879 (484,1283)	3.27 (1.83,4.76)	16.99 (14.24,19.81)

Sub-Saharan Africa	881 (704,1107)	0.22 (0.18,0.28)	9.44 (7.94,11.02)	21630 (17394,27254)	4.68 (3.75,5.89)	9.72 (8.19,11.18)
Sudan	91 (61,133)	0.55 (0.37,0.8)	20.27 (16.18,24.34)	2226 (1476,3246)	11.77 (7.89,17.08)	21.02 (16.98,25.13)
Suriname	2 (1,3)	0.32 (0.21,0.45)	19.84 (16.31,23.54)	47 (31,66)	7.28 (4.81,10.26)	21.65 (18.03,25.39)
Sweden	192 (145,240)	0.77 (0.59,0.95)	22.82 (18.32,27.67)	3423 (2687,4223)	15.22 (12.1,18.71)	25.44 (20.9,30.06)
Switzerland	182 (144,223)	0.87 (0.7,1.06)	28.84 (24.37,33.52)	3234 (2620,3928)	17.18 (13.93,20.79)	30.38 (25.76,35.04)
Syrian Arab Republic	83 (55,119)	0.76 (0.5,1.1)	30.54 (25.41,35.68)	2084 (1366,3010)	15.93 (10.52,22.77)	31.85 (27.31,36.33)
Taiwan (Province of China)	382 (313,451)	0.87 (0.71,1.02)	27.17 (23.31,31.09)	7420 (6167,8646)	17.33 (14.43,20.11)	27.6 (24.07,31.24)
Tajikistan	8 (5,12)	0.16 (0.11,0.24)	21.51 (17.15,26.26)	218 (135,330)	3.66 (2.32,5.51)	22.23 (17.86,27.07)
Thailand	639 (456,890)	0.59 (0.42,0.81)	28.52 (24.39,32.38)	13031 (9172,18369)	11.87 (8.39,16.75)	27.42 (23.58,31.1)
Timor-Leste	2 (1,3)	0.26 (0.17,0.43)	25.71 (20.75,31.46)	43 (28,68)	5.23 (3.36,8.23)	25.09 (20.4,30.52)
Togo	9 (6,13)	0.28 (0.19,0.4)	13.31 (10.44,16.44)	240 (164,347)	6.25 (4.27,8.89)	14.01 (11.08,16.99)
Tokelau	0 (0,0)	0.3 (0.19,0.43)	19.12 (14.93,23.79)	0 (0,0)	7.21 (4.5,10.26)	20.57 (16.43,25.52)

Tonga	0 (0,1)	0.44 (0.27,0.7)	30.39 (24.72,36.39)	8 (5,12)	9.56 (5.67,15.53)	29.9 (24.37,35.29)
Trinidad and Tobago	6 (4,8)	0.3 (0.2,0.4)	16.02 (13.04,19.37)	134 (94,179)	6.7 (4.67,8.94)	17.18 (14.33,20.4)
Tropical Latin America	1258 (1020,1549)	0.5 (0.41,0.62)	21.14 (17.27,25.53)	26514 (21772,32137)	10.32 (8.46,12.53)	21.86 (18.13,25.99)
Tunisia	165 (103,235)	1.35 (0.84,1.91)	38.78 (33.19,44.03)	3535 (2210,5182)	27.03 (16.89,39.48)	38.99 (33.99,44.17)
Turkey	1172 (852,1599)	1.29 (0.94,1.76)	32.29 (27.56,36.94)	28109 (20166,38203)	29.46 (21.19,40.03)	34.97 (30.08,39.54)
Turkmenistan	10 (7,14)	0.26 (0.19,0.36)	22.89 (19.1,26.64)	296 (210,407)	6.85 (4.88,9.4)	23.99 (20.25,27.87)
Tuvalu	0 (0,0)	0.36 (0.24,0.52)	21.39 (17.75,26.34)	1 (1,1)	8.97 (6.01,12.41)	22.7 (18.91,27.75)
Uganda	23 (17,32)	0.2 (0.14,0.27)	6.53 (5.24,8.07)	549 (397,789)	3.98 (2.9,5.58)	6.65 (5.38,8.28)
Ukraine	697 (454,972)	0.87 (0.57,1.22)	30.92 (25.29,36.25)	16935 (10969,23876)	21.8 (14.06,30.89)	32.82 (27.07,37.98)
United Arab Emirates	17 (12,25)	0.83 (0.58,1.16)	18.12 (14.43,22.22)	580 (405,815)	16.13 (11.23,22.67)	18.47 (14.89,22.33)
United Kingdom	1633 (1288,2023)	1.12 (0.89,1.37)	24.43 (19.79,29.98)	28486 (23272,34318)	21.28 (17.62,25.31)	26.35 (21.87,31.38)
United Republic of Tanzania	55 (37,80)	0.26 (0.17,0.38)	10.69 (8.28,13.02)	1277 (852,1871)	5.24 (3.53,7.58)	10.81 (8.33,13.03)

United States of America	4957 (3948,6196)	0.8 (0.64,1)	23.52 (19.39,28.5)	107083 (87699,129617)	18.13 (14.89,21.81)	26.6 (22.34,31.4)
United States Virgin Islands	0 (0,0)	0.16 (0.1,0.23)	13.4 (9.86,17.73)	6 (4,10)	3.38 (2.16,4.98)	14.52 (10.64,18.97)
Uruguay	73 (58,89)	1.26 (1.02,1.52)	24.94 (20.65,29.66)	1519 (1240,1819)	28.43 (23.21,33.76)	28.38 (23.9,33.06)
Uzbekistan	59 (43,79)	0.24 (0.18,0.33)	20.79 (17.21,24.35)	1601 (1147,2116)	5.86 (4.24,7.78)	21.41 (18.05,24.95)
Vanuatu	0 (0,1)	0.23 (0.14,0.34)	13.93 (11.24,17.05)	10 (6,15)	5.43 (3.29,8.28)	14.46 (11.77,17.32)
Venezuela (Bolivarian Republic of)	74 (52,101)	0.26 (0.18,0.36)	14.1 (11.42,17.12)	1605 (1131,2225)	5.37 (3.8,7.38)	14.16 (11.72,16.98)
Viet Nam	351 (266,447)	0.39 (0.3,0.5)	32.91 (28.11,37.52)	8296 (6134,10720)	8.4 (6.3,10.8)	34.26 (29.81,38.82)
Western Europe	11200 (9033,13504)	1.08 (0.88,1.28)	25.16 (21.11,29.56)	211726 (175874,251353)	22.82 (19.09,26.8)	28.07 (24.04,32.47)
Western Sub-Saharan Africa	223 (170,292)	0.14 (0.11,0.18)	7.53 (6.12,9.2)	5355 (4099,7041)	2.84 (2.17,3.72)	7.71 (6.29,9.32)
Yemen	102 (69,141)	0.85 (0.58,1.18)	28.72 (24.37,33.41)	2582 (1701,3611)	18.7 (12.5,25.95)	30.49 (26.02,35.01)
Zambia	17 (10,32)	0.32 (0.19,0.55)	9.18 (7.04,11.57)	424 (234,836)	6.64 (3.78,12.16)	8.9 (6.55,11.16)
Zimbabwe	84 (62,114)	1.53 (1.13,2.04)	19.64 (15.53,24.04)	2109 (1547,2893)	32.09 (23.78,43.23)	19.12 (15.08,23.4)

ASMR, age-standardized mortality rates; ASDR, age-standardized disability-adjusted life years rates; DALYs, disability-adjusted life years; PAF, population

attributable fraction; ASM, age-standardized mortality; ASD, age-standardized disability-adjusted life year; UI, uncertainty interval.

Supplementary Table 3. Decomposition analysis of bladder, kidney, and prostate cancers attributable to smoking.

		sex_name	Overll difference	Aging	Population	Epidemiological change	a_percent	p_percent	r_percent
Bladder cancer	Mortality	Both	26938.45	14255.87	48819.95	-36137.4	52.92	181.23	-134.15
		Male	26492.62	17217.68	45622.16	-36347.2	64.99	172.21	-137.2
		Female	1482.34	1529.47	5466.701	-5513.83	103.18	368.79	-371.97
	DALYs	Both	401764.8	217384.9	983102.1	-798722	54.11	244.7	-198.8
		Male	386917.1	251962.3	914715.7	-779761	65.12	236.41	-201.53
		Female	18050.95	20987.93	99626.61	-102564	116.27	551.92	-568.19
Kidney cancer	Mortality	Both	9330.47	2692.672	11305.08	-4667.28	28.86	121.16	-50.02
		Male	8792.01	2880.207	9860.827	-3949.02	32.76	112.16	-44.92
		Female	794.36	398.666	1750.947	-1355.25	50.19	220.42	-170.61
	DALYs	Both	371529.9	-34966.7	357137.3	49359.32	-9.41	96.13	13.29
		Male	364113.2	-36043.2	361633	38523.42	-9.9	99.32	10.58
		Female	43733.72	-3884.07	60177.74	-12560	-8.88	137.6	-28.72
Prostate cancer	Mortality	Male	4346.12	5069.376	12465.44	-13188.7	116.64	286.82	-303.46
	DALYs	Male	77600.94	75408.95	243310.1	-241118	97.18	313.54	-310.72

Supplementary Table 4. Autoregressive integrated moving average (ARIMA) model predicts disease burden over the next 20 years.

	Bladder cancer		Kidney cancer		Prostate cancer	
Year	ASMR (95% CI)	ASDR (95% CI)	ASMR (95% CI)	ASDR (95% CI)	ASMR (95% CI)	ASDR (95% CI)
2022	0.69 (0.68, 0.70)	14.12 (13.91, 14.34)	0.19 (0.18, 0.19)	4.29 (4.21, 4.38)	0.35 (0.35, 0.36)	7.07 (6.97, 7.18)
2023	0.68 (0.66, 0.70)	13.88 (13.47, 14.30)	0.18 (0.18, 0.19)	4.22 (4.06, 4.37)	0.35 (0.33, 0.36)	6.96 (6.75, 7.18)
2024	0.67 (0.64, 0.69)	13.62 (13.01, 14.22)	0.18 (0.17, 0.19)	4.14 (3.90, 4.38)	0.34 (0.32, 0.36)	6.84 (6.50, 7.18)

2025	0.65 (0.62, 0.69)	13.34 (12.56, 14.12)	0.18 (0.17, 0.19)	4.06 (3.74, 4.39)	0.34 (0.31, 0.37)	6.72 (6.24, 7.19)
2026	0.64 (0.60, 0.68)	13.05 (12.11, 14.00)	0.17 (0.16, 0.19)	3.98 (3.56, 4.40)	0.33 (0.29, 0.37)	6.58 (5.98, 7.19)
2027	0.63 (0.58, 0.68)	12.76 (11.67, 13.86)	0.17 (0.15, 0.19)	3.91 (3.38, 4.43)	0.33 (0.28, 0.38)	6.45 (5.70, 7.19)
2028	0.61 (0.56, 0.67)	12.47 (11.24, 13.70)	0.17 (0.15, 0.19)	3.83 (3.19, 4.46)	0.32 (0.26, 0.38)	6.31 (5.43, 7.18)
2029	0.60 (0.54, 0.66)	12.17 (10.81, 13.53)	0.16 (0.14, 0.19)	3.75 (3.00, 4.50)	0.32 (0.25, 0.39)	6.16 (5.15, 7.17)
2030	0.59 (0.52, 0.65)	11.87 (10.39, 13.35)	0.16 (0.13, 0.19)	3.67 (2.79, 4.55)	0.32 (0.23, 0.40)	6.01 (4.88, 7.15)
2031	0.57 (0.50, 0.64)	11.57 (9.98, 13.16)	0.16 (0.13, 0.19)	3.60 (2.59, 4.60)	0.31 (0.22, 0.41)	5.86 (4.60, 7.12)
2032	0.56 (0.49, 0.64)	11.27 (9.58, 12.96)	0.15 (0.12, 0.19)	3.52 (2.37, 4.66)	0.31 (0.20, 0.42)	5.71 (4.33, 7.09)
2033	0.55 (0.47, 0.63)	10.97 (9.18, 12.76)	0.15 (0.11, 0.19)	3.44 (2.15, 4.73)	0.31 (0.19, 0.42)	5.55 (4.05, 7.06)
2034	0.54 (0.45, 0.62)	10.67 (8.79, 12.56)	0.15 (0.11, 0.19)	3.36 (1.93, 4.80)	0.30 (0.17, 0.43)	5.40 (3.78, 7.02)
2035	0.52 (0.44, 0.61)	10.37 (8.40, 12.35)	0.15 (0.10, 0.19)	3.29 (1.70, 4.87)	0.30 (0.16, 0.44)	5.24 (3.51, 6.97)
2036	0.51 (0.42, 0.60)	10.07 (8.01, 12.13)	0.14 (0.09, 0.19)	3.21 (1.47, 4.95)	0.30 (0.14, 0.45)	5.08 (3.24, 6.92)
2037	0.50 (0.40, 0.59)	9.77 (7.63, 11.91)	0.14 (0.08, 0.20)	3.13 (1.23, 5.03)	0.29 (0.12, 0.46)	4.92 (2.98, 6.87)
2038	0.48 (0.38, 0.58)	9.47 (7.24, 11.69)	0.14 (0.07, 0.20)	3.05 (0.98, 5.12)	0.29 (0.11, 0.47)	4.76 (2.72, 6.81)
2039	0.47 (0.37, 0.57)	9.17 (6.87, 11.47)	0.13 (0.07, 0.20)	2.98 (0.73, 5.22)	0.29 (0.09, 0.49)	4.60 (2.46, 6.75)
2040	0.46 (0.35, 0.56)	8.87 (6.49, 11.24)	0.13 (0.06, 0.20)	2.90 (0.48, 5.32)	0.29 (0.08, 0.50)	4.44 (2.20, 6.68)
2041	0.44 (0.34, 0.55)	8.56 (6.12, 11.01)	0.13 (0.05, 0.20)	2.82 (0.22, 5.42)	0.28 (0.06, 0.51)	4.28 (1.94, 6.61)

ASMR, age-standardized mortality rates; ASDR, and age-standardized disability-adjusted life years rates; CI, confidence interval.

Supplementary Table 5. Global average annual percentage change (AAPC) of age-standardized mortality rates (ASMR) and age-standardized disability-adjusted life year rates (ASDR) calculated for different time windows (10, 15, 20, and 25 years).

	Bladder cancer		Kidney cancer		Prostate cancer	

Start time	End time	Time size	AAPC of ASMR (95% CI)	AAPC of ASDR (95% CI)	AAPC of ASMR (95% CI)	AAPC of ASDR (95% CI)	AAPC of ASMR (95% CI)	AAPC of ASDR (95% CI)
1990	1999	10 years	-1.05 (-1.07, -1.03)	-1.12 (-1.14, -1.1)	-0.23 (-0.26, -0.21)	-0.4 (-0.43, -0.38)	-1.51 (-1.54, -1.48)	-1.45 (-1.48, -1.42)
1991	2000	10 years	-1.15 (-1.17, -1.13)	-1.23 (-1.25, -1.21)	-0.24 (-0.26, -0.22)	-0.41 (-0.44, -0.39)	-1.75 (-1.77, -1.72)	-1.66 (-1.69, -1.64)
1992	2001	10 years	-1.32 (-1.33, -1.31)	-1.41 (-1.43, -1.4)	-0.34 (-0.35, -0.32)	-0.52 (-0.54, -0.5)	-1.94 (-1.96, -1.92)	-1.83 (-1.85, -1.81)
1993	2002	10 years	-1.52 (-1.53, -1.51)	-1.63 (-1.64, -1.62)	-0.52 (-0.53, -0.51)	-0.71 (-0.72, -0.69)	-2.14 (-2.15, -2.13)	-2 (-2.01, -1.99)
1994	2003	10 years	-1.67 (-1.68, -1.67)	-1.81 (-1.82, -1.8)	-0.58 (-0.6, -0.57)	-0.78 (-0.8, -0.77)	-2.35 (-2.36, -2.35)	-2.19 (-2.2, -2.19)
1995	2004	10 years	-1.79 (-1.8, -1.78)	-1.94 (-1.95, -1.93)	-0.61 (-0.62, -0.6)	-0.78 (-0.8, -0.77)	-2.51 (-2.52, -2.51)	-2.33 (-2.34, -2.32)

1996	2005	10 years	-1.82 (-1.83, -1.81)	-1.94 (-1.95, -1.93)	-0.47 (-0.47, -0.46)	-0.61 (-0.62, -0.6)	-2.68 (-2.69, -2.67)	-2.45 (-2.45, -2.44)
1997	2006	10 years	-1.99 (-2.01, -1.98)	-2.09 (-2.11, -2.08)	-0.57 (-0.58, -0.56)	-0.7 (-0.71, -0.69)	-2.77 (-2.78, -2.75)	-2.51 (-2.52, -2.5)
1998	2007	10 years	-2.07 (-2.08, -2.05)	-2.18 (-2.2, -2.17)	-0.66 (-0.68, -0.65)	-0.79 (-0.81, -0.77)	-2.86 (-2.88, -2.85)	-2.61 (-2.63, -2.6)
1999	2008	10 years	-2.08 (-2.09, -2.07)	-2.21 (-2.22, -2.2)	-0.63 (-0.64, -0.61)	-0.76 (-0.78, -0.74)	-2.89 (-2.91, -2.88)	-2.62 (-2.63, -2.61)
2000	2009	10 years	-2.12 (-2.13, -2.11)	-2.27 (-2.28, -2.26)	-0.81 (-0.83, -0.8)	-0.95 (-0.96, -0.93)	-2.97 (-2.99, -2.96)	-2.68 (-2.69, -2.67)
2001	2010	10 years	-2.01 (-2.03, -2)	-2.15 (-2.16, -2.13)	-0.82 (-0.84, -0.81)	-0.97 (-0.99, -0.96)	-3.04 (-3.05, -3.03)	-2.73 (-2.74, -2.72)
2002	2011	10 years	-1.98 (-1.99, -1.96)	-2.11 (-2.12, -2.09)	-0.92 (-0.93, -0.91)	-1.1 (-1.11, -1.09)	-3.04 (-3.05, -3.04)	-2.77 (-2.77, -2.76)

2003	2012	10 years	-1.98 (-2, -1.97)	-2.1 (-2.11, -2.08)	-1.05 (-1.06, -1.04)	-1.25 (-1.26, -1.24)	-3.09 (-3.1, -3.08)	-2.81 (-2.82, -2.81)
2004	2013	10 years	-1.98 (-1.99, -1.96)	-2.1 (-2.11, -2.09)	-1.16 (-1.17, -1.15)	-1.38 (-1.4, -1.37)	-3.1 (-3.11, -3.09)	-2.86 (-2.87, -2.86)
2005	2014	10 years	-1.96 (-1.97, -1.94)	-2.12 (-2.13, -2.11)	-1.37 (-1.38, -1.36)	-1.64 (-1.65, -1.62)	-2.89 (-2.9, -2.88)	-2.73 (-2.73, -2.72)
2006	2015	10 years	-1.67 (-1.68, -1.66)	-1.88 (-1.89, -1.87)	-1.24 (-1.25, -1.23)	-1.54 (-1.56, -1.53)	-2.59 (-2.61, -2.57)	-2.5 (-2.51, -2.49)
2007	2016	10 years	-1.51 (-1.52, -1.5)	-1.73 (-1.74, -1.72)	-1.25 (-1.26, -1.24)	-1.57 (-1.58, -1.56)	-2.3 (-2.33, -2.28)	-2.24 (-2.26, -2.22)
2008	2017	10 years	-1.57 (-1.58, -1.56)	-1.78 (-1.79, -1.77)	-1.52 (-1.53, -1.5)	-1.84 (-1.85, -1.83)	-2.21 (-2.24, -2.18)	-2.13 (-2.16, -2.11)
2009	2018	10 years	-1.56 (-1.57, -1.55)	-1.77 (-1.78, -1.77)	-1.54 (-1.56, -1.53)	-1.89 (-1.9, -1.88)	-2 (-2.02, -1.97)	-1.94 (-1.97, -1.92)

2010	2019	10 years	-1.59 (-1.6, -1.58)	-1.82 (-1.83, -1.81)	-1.63 (-1.64, -1.63)	-1.98 (-1.99, -1.98)	-1.8 (-1.83, -1.78)	-1.79 (-1.81, -1.76)
2011	2020	10 years	-1.52 (-1.53, -1.51)	-1.76 (-1.77, -1.75)	-1.71 (-1.71, -1.7)	-2.04 (-2.05, -2.04)	-1.7 (-1.73, -1.68)	-1.69 (-1.71, -1.67)
2012	2021	10 years	-1.42 (-1.43, -1.41)	-1.64 (-1.65, -1.63)	-1.69 (-1.7, -1.69)	-2.01 (-2.01, -2)	-1.53 (-1.54, -1.51)	-1.5 (-1.52, -1.49)
1990	2004	15 years	-1.36 (-1.39, -1.34)	-1.47 (-1.5, -1.45)	-0.27 (-0.29, -0.25)	-0.42 (-0.44, -0.39)	-1.91 (-1.95, -1.87)	-1.78 (-1.82, -1.75)
1991	2005	15 years	-1.46 (-1.49, -1.44)	-1.57 (-1.6, -1.55)	-0.3 (-0.32, -0.28)	-0.44 (-0.46, -0.42)	-2.12 (-2.16, -2.09)	-1.95 (-1.98, -1.92)
1992	2006	15 years	-1.69 (-1.71, -1.67)	-1.79 (-1.82, -1.77)	-0.49 (-0.5, -0.47)	-0.64 (-0.65, -0.62)	-2.35 (-2.38, -2.32)	-2.16 (-2.18, -2.13)
1993	2007	15 years	-1.81 (-1.83, -1.79)	-1.94 (-1.96, -1.91)	-0.67 (-0.68, -0.65)	-0.84 (-0.86, -0.82)	-2.53 (-2.55, -2.5)	-2.33 (-2.35, -2.31)

1994	2008	15 years	-1.88 (-1.89, -1.86)	-2 (-2.02, -1.98)	-0.72 (-0.73, -0.7)	-0.9 (-0.91, -0.88)	-2.67 (-2.69, -2.64)	-2.45 (-2.47, -2.44)
1995	2009	15 years	-1.93 (-1.95, -1.92)	-2.06 (-2.08, -2.04)	-0.77 (-0.79, -0.76)	-0.95 (-0.96, -0.93)	-2.77 (-2.79, -2.75)	-2.54 (-2.56, -2.53)
1996	2010	15 years	-1.87 (-1.88, -1.85)	-1.99 (-2, -1.97)	-0.71 (-0.73, -0.69)	-0.88 (-0.9, -0.86)	-2.83 (-2.85, -2.81)	-2.59 (-2.6, -2.57)
1997	2011	15 years	-1.88 (-1.9, -1.87)	-1.99 (-2.01, -1.98)	-0.72 (-0.74, -0.71)	-0.9 (-0.92, -0.88)	-2.82 (-2.83, -2.8)	-2.57 (-2.58, -2.56)
1998	2012	15 years	-1.9 (-1.92, -1.89)	-2.02 (-2.04, -2.01)	-0.78 (-0.8, -0.76)	-0.95 (-0.97, -0.93)	-2.87 (-2.89, -2.86)	-2.63 (-2.64, -2.61)
1999	2013	15 years	-1.96 (-1.97, -1.94)	-2.1 (-2.11, -2.09)	-0.87 (-0.89, -0.85)	-1.05 (-1.07, -1.03)	-2.93 (-2.94, -2.92)	-2.69 (-2.7, -2.68)
2000	2014	15 years	-1.98 (-1.99, -1.97)	-2.14 (-2.16, -2.13)	-1.03 (-1.05, -1.01)	-1.23 (-1.25, -1.2)	-2.86 (-2.87, -2.85)	-2.63 (-2.64, -2.62)

2001	2015	15 years	-1.91 (-1.93, -1.9)	-2.09 (-2.11, -2.08)	-1.07 (-1.08, -1.05)	-1.3 (-1.32, - 1.27)	-2.77 (- 2.79, -2.75)	-2.58 (-2.6, -2.57)
2002	2016	15 years	-1.81 (-1.82, -1.79)	-2 (-2.02, - 1.98)	-1.14 (-1.16, -1.12)	-1.39 (-1.41, -1.37)	-2.63 (- 2.66, -2.6)	-2.48 (-2.5, -2.46)
2003	2017	15 years	-1.81 (-1.83, -1.79)	-1.98 (-2, - 1.97)	-1.32 (-1.34, -1.3)	-1.57 (-1.6, - 1.55)	-2.57 (- 2.61, -2.54)	-2.41 (- 2.44, -2.38)
2004	2018	15 years	-1.79 (-1.8, -1.77)	-1.96 (-1.97, -1.94)	-1.38 (-1.39, -1.36)	-1.66 (-1.68, -1.64)	-2.44 (- 2.48, -2.4)	-2.3 (-2.33, -2.26)
2005	2019	15 years	-1.72 (-1.74, -1.71)	-1.91 (-1.92, -1.89)	-1.46 (-1.48, -1.44)	-1.76 (-1.78, -1.74)	-2.27 (- 2.31, -2.22)	-2.16 (-2.2, -2.13)
2006	2020	15 years	-1.58 (-1.59, -1.57)	-1.78 (-1.79, -1.77)	-1.46 (-1.47, -1.44)	-1.76 (-1.78, -1.74)	-2.13 (- 2.17, -2.09)	-2.05 (- 2.08, -2.01)
2007	2021	15 years	-1.49 (-1.5, -1.48)	-1.67 (-1.68, -1.66)	-1.44 (-1.46, -1.43)	-1.73 (-1.75, -1.71)	-2.02 (- 2.05, -1.98)	-1.91 (- 1.95, -1.88)

1990	2009	20 years	-1.58 (-1.62, -1.54)	-1.69 (-1.72, -1.65)	-0.48 (-0.51, -0.46)	-0.63 (-0.66, -0.61)	-2.26 (-2.31, -2.21)	-2.09 (-2.13, -2.04)
1991	2010	20 years	-1.59 (-1.62, -1.56)	-1.7 (-1.74, -1.67)	-0.52 (-0.55, -0.5)	-0.68 (-0.71, -0.66)	-2.38 (-2.42, -2.34)	-2.19 (-2.22, -2.15)
1992	2011	20 years	-1.69 (-1.71, -1.66)	-1.8 (-1.83, -1.77)	-0.62 (-0.64, -0.6)	-0.8 (-0.82, -0.78)	-2.5 (-2.53, -2.46)	-2.29 (-2.32, -2.26)
1993	2012	20 years	-1.76 (-1.78, -1.74)	-1.88 (-1.9, -1.86)	-0.75 (-0.77, -0.73)	-0.94 (-0.97, -0.92)	-2.62 (-2.65, -2.59)	-2.41 (-2.44, -2.39)
1994	2013	20 years	-1.84 (-1.86, -1.83)	-1.98 (-1.99, -1.96)	-0.87 (-0.89, -0.85)	-1.07 (-1.1, -1.05)	-2.75 (-2.77, -2.73)	-2.55 (-2.57, -2.53)
1995	2014	20 years	-1.88 (-1.89, -1.86)	-2.02 (-2.04, -2.01)	-0.94 (-0.97, -0.92)	-1.15 (-1.18, -1.12)	-2.74 (-2.76, -2.72)	-2.55 (-2.56, -2.53)
1996	2015	20 years	-1.83 (-1.85, -1.82)	-1.99 (-2, -1.97)	-0.92 (-0.95, -0.89)	-1.14 (-1.18, -1.11)	-2.69 (-2.7, -2.67)	-2.51 (-2.53, -2.5)

1997	2016	20 years	-1.78 (-1.8, -1.76)	-1.94 (-1.96, -1.93)	-0.94 (-0.97, -0.91)	-1.17 (-1.2, - 1.14)	-2.57 (-2.6, -2.55)	-2.41 (- 2.43, -2.39)
1998	2017	20 years	-1.8 (-1.81, -1.78)	-1.96 (-1.97, -1.94)	-1.05 (-1.08, -1.02)	-1.27 (-1.31, -1.23)	-2.55 (- 2.58, -2.52)	-2.38 (-2.4, -2.35)
1999	2018	20 years	-1.82 (-1.84, -1.8)	-1.99 (-2.01, -1.98)	-1.11 (-1.14, -1.08)	-1.34 (-1.37, -1.3)	-2.49 (- 2.53, -2.45)	-2.32 (- 2.35, -2.29)
2000	2019	20 years	-1.8 (-1.82, -1.78)	-1.98 (-2, - 1.96)	-1.18 (-1.21, -1.16)	-1.43 (-1.46, -1.39)	-2.41 (- 2.45, -2.36)	-2.24 (- 2.28, -2.21)
2001	2020	20 years	-1.78 (-1.8, -1.76)	-1.96 (-1.98, -1.94)	-1.27 (-1.3, -1.25)	-1.52 (-1.55, -1.49)	-2.39 (- 2.44, -2.33)	-2.23 (- 2.27, -2.19)
2002	2021	20 years	-1.71 (-1.73, -1.69)	-1.89 (-1.91, -1.87)	-1.31 (-1.33, -1.29)	-1.56 (-1.59, -1.53)	-2.33 (- 2.39, -2.28)	-2.18 (- 2.23, -2.13)
1990	2014	25 years	-1.61 (-1.64, -1.58)	-1.74 (-1.77, -1.7)	-0.68 (-0.71, -0.64)	-0.86 (-0.9, - 0.82)	-2.34 (- 2.39, -2.29)	-2.18 (- 2.23, -2.13)

1991	2015	25 years	-1.62 (-1.65, -1.59)	-1.76 (-1.79, -1.73)	-0.73 (-0.76, -0.7)	-0.93 (-0.97, -0.89)	-2.36 (-2.4, -2.32)	-2.21 (-2.25, -2.17)
1992	2016	25 years	-1.65 (-1.67, -1.62)	-1.8 (-1.82, -1.78)	-0.81 (-0.85, -0.78)	-1.03 (-1.07, -1)	-2.37 (-2.4, -2.33)	-2.23 (-2.26, -2.2)
1993	2017	25 years	-1.7 (-1.72, -1.68)	-1.86 (-1.88, -1.84)	-0.97 (-1, -0.94)	-1.2 (-1.24, -1.16)	-2.42 (-2.45, -2.39)	-2.26 (-2.29, -2.23)
1994	2018	25 years	-1.76 (-1.78, -1.74)	-1.92 (-1.93, -1.9)	-1.06 (-1.09, -1.02)	-1.3 (-1.34, -1.25)	-2.44 (-2.47, -2.4)	-2.28 (-2.31, -2.25)
1995	2019	25 years	-1.76 (-1.78, -1.74)	-1.92 (-1.94, -1.9)	-1.08 (-1.12, -1.05)	-1.33 (-1.37, -1.28)	-2.41 (-2.45, -2.36)	-2.25 (-2.29, -2.22)
1996	2020	25 years	-1.74 (-1.76, -1.72)	-1.91 (-1.92, -1.89)	-1.11 (-1.15, -1.07)	-1.36 (-1.4, -1.31)	-2.4 (-2.45, -2.35)	-2.25 (-2.29, -2.21)
1997	2021	25 years	-1.71 (-1.74, -1.69)	-1.87 (-1.89, -1.85)	-1.12 (-1.16, -1.07)	-1.35 (-1.4, -1.3)	-2.35 (-2.4, -2.3)	-2.19 (-2.23, -2.14)

AAPC, average annual percentage change; ASMR, age-standardized mortality rates; ASDR, age-standardized disability-adjusted life year rates; CI, confidence interval.

Supplementary Table 6. Average annual percentage change (AAPC) and estimated annual percentage change (EAPC) of age-standardized mortality rates (ASMR) and age-standardized disability-adjusted life year rates (ASDR) for kidney cancer attributable to smoking.

location_name	AAPC of ASMR (95% CI)	EAPC of ASMR (95% CI)	AAPC of ASDR (95% CI)	EAPC of ASDR (95% CI)
Afghanistan	0.78 (0.71,0.85)	1.03 (0.89,1.17)	0.79 (0.72,0.87)	0.99 (0.85,1.13)
Albania	1.40 (1.31,1.48)	1.87 (1.7,2.04)	1.30 (1.21,1.39)	1.8 (1.62,1.99)
Algeria	0.90 (0.86,0.93)	1.11 (1.04,1.18)	0.89 (0.87,0.92)	1 (0.96,1.04)
American Samoa	0.31 (0.15,0.48)	0.64 (0.31,0.96)	0.25 (0.08,0.42)	0.56 (0.23,0.89)
Andean Latin America	0.37 (0.30,0.44)	0.52 (0.39,0.66)	0.24 (0.17,0.30)	0.35 (0.23,0.48)
Andorra	-1.79 (-1.88,-1.70)	-1.56 (-1.72,-1.39)	-1.87 (-1.95,-1.78)	-1.61 (-1.77,-1.45)
Angola	0.73 (0.59,0.87)	0.89 (0.62,1.16)	0.79 (0.64,0.94)	0.95 (0.67,1.24)
Antigua and Barbuda	-0.07 (-0.21,0.07)	0 (-0.26,0.27)	-0.26 (-0.39,-0.12)	-0.2 (-0.46,0.06)

Argentina	0.30 (0.17,0.42)	0.43 (0.19,0.68)	0.10 (-0.02,0.22)	0.21 (-0.02,0.45)
Armenia	1.03 (0.85,1.22)	1.49 (1.13,1.85)	0.86 (0.67,1.05)	1.33 (0.96,1.7)
Australasia	-1.97 (-2.01,-1.94)	-2.09 (-2.16,-2.03)	-2.15 (-2.19,-2.11)	-2.22 (-2.29,-2.15)
Australia	-2.14 (-2.18,-2.10)	-2.26 (-2.34,-2.18)	-2.29 (-2.33,-2.25)	-2.33 (-2.41,-2.25)
Austria	-1.52 (-1.57,-1.46)	-1.24 (-1.35,-1.13)	-1.71 (-1.77,-1.66)	-1.43 (-1.54,-1.31)
Azerbaijan	1.06 (0.95,1.17)	1.55 (1.34,1.77)	0.85 (0.75,0.94)	1.23 (1.05,1.41)
Bahrain	-0.20 (-0.33,-0.08)	-0.7 (-0.94,-0.47)	-0.31 (-0.44,-0.17)	-0.79 (-1.05,-0.53)
Bangladesh	-0.71 (-0.76,-0.66)	-0.82 (-0.91,-0.72)	-0.89 (-0.93,-0.86)	-0.94 (-1.01,-0.87)
Barbados	-0.30 (-0.44,-0.17)	-0.4 (-0.67,-0.14)	-0.59 (-0.72,-0.47)	-0.61 (-0.85,-0.37)
Belarus	3.09 (2.69,3.49)	2.73 (1.94,3.52)	2.98 (2.56,3.39)	2.58 (1.76,3.39)

Belgium	-1.47 (-1.61,-1.34)	-1.51 (-1.77,-1.26)	-1.67 (-1.83,-1.52)	-1.66 (-1.95,-1.37)
Belize	0.60 (0.33,0.86)	0.7 (0.18,1.21)	0.48 (0.23,0.74)	0.62 (0.13,1.11)
Benin	0.65 (0.53,0.78)	0.54 (0.3,0.78)	0.69 (0.56,0.82)	0.58 (0.33,0.82)
Bermuda	-1.25 (-1.37,-1.13)	-0.83 (-1.05,-0.6)	-1.29 (-1.42,-1.17)	-0.91 (-1.14,-0.68)
Bhutan	0.65 (0.63,0.67)	0.59 (0.55,0.63)	0.48 (0.45,0.50)	0.41 (0.36,0.46)
Bolivarian Republic of Venezuela	-0.88 (-0.96,-0.80)	-0.75 (-0.91,-0.6)	-1.08 (-1.16,-1.00)	-0.97 (-1.13,-0.81)
Bosnia and Herzegovina	2.46 (2.21,2.71)	3.27 (2.78,3.76)	2.35 (2.10,2.60)	3.18 (2.69,3.67)
Botswana	0.81 (0.76,0.86)	0.73 (0.63,0.82)	0.77 (0.71,0.83)	0.65 (0.53,0.77)
Brazil	-0.44 (-0.48,-0.40)	-0.52 (-0.6,-0.44)	-0.68 (-0.72,-0.63)	-0.81 (-0.9,-0.72)
Brunei Darussalam	-1.29 (-1.41,-1.16)	-0.69 (-0.92,-0.45)	-1.36 (-1.49,-1.24)	-0.84 (-1.08,-0.59)

Bulgaria	2.88 (2.60,3.16)	3.8 (3.25,4.35)	2.93 (2.65,3.21)	3.77 (3.22,4.33)
Burkina Faso	2.13 (2.11,2.16)	2.26 (2.2,2.32)	2.08 (2.04,2.11)	2.16 (2.09,2.23)
Burundi	-1.48 (-1.67,-1.30)	-1.4 (-1.76,-1.05)	-1.52 (-1.71,-1.33)	-1.44 (-1.79,-1.09)
Cambodia	0.45 (0.42,0.47)	0.34 (0.29,0.38)	0.40 (0.36,0.43)	0.25 (0.18,0.31)
Cameroon	1.55 (1.48,1.61)	1.71 (1.58,1.84)	1.58 (1.50,1.65)	1.72 (1.58,1.86)
Canada	-1.17 (-1.27,-1.07)	-0.96 (-1.15,-0.77)	-1.48 (-1.59,-1.37)	-1.3 (-1.5,-1.09)
Caribbean	-0.16 (-0.22,-0.11)	-0.15 (-0.26,-0.03)	-0.27 (-0.33,-0.21)	-0.25 (-0.36,-0.13)
Central African Republic	-0.54 (-0.61,-0.47)	-0.58 (-0.72,-0.44)	-0.51 (-0.59,-0.43)	-0.57 (-0.72,-0.41)
Central Asia	1.16 (0.98,1.34)	1.35 (1,1.7)	0.93 (0.75,1.10)	1.03 (0.69,1.38)
Central Europe	-0.11 (-0.22,-0.00)	-0.05 (-0.26,0.16)	-0.31 (-0.43,-0.18)	-0.24 (-0.47,0)

Central Europe, Eastern Europe, and Central Asia	0.33 (0.19,0.48)	0.19 (-0.08,0.46)	0.13 (-0.02,0.29)	-0.05 (-0.35,0.24)
Central Latin America	-1.24 (-1.30,-1.18)	-1.38 (-1.49,-1.27)	-1.28 (-1.34,-1.22)	-1.46 (-1.57,-1.34)
Central Sub-Saharan Africa	0.58 (0.42,0.75)	0.76 (0.43,1.08)	0.66 (0.50,0.81)	0.83 (0.52,1.13)
Chad	2.07 (1.96,2.17)	2.05 (1.84,2.25)	2.13 (2.02,2.24)	2.14 (1.93,2.35)
Chile	-0.98 (-1.05,-0.91)	-0.78 (-0.91,-0.65)	-1.21 (-1.27,-1.15)	-1.01 (-1.13,-0.89)
China	0.85 (0.76,0.94)	1.14 (0.97,1.31)	0.93 (0.83,1.03)	1.29 (1.09,1.49)
Colombia	-1.50 (-1.60,-1.41)	-1.94 (-2.12,-1.76)	-1.74 (-1.84,-1.65)	-2.21 (-2.4,-2.03)
Commonwealth of the Bahamas	0.29 (0.21,0.38)	0.58 (0.41,0.76)	0.12 (0.02,0.21)	0.41 (0.23,0.59)
Comoros	0.13 (0.10,0.17)	-0.07 (-0.14,-0.01)	-0.02 (-0.07,0.02)	-0.28 (-0.37,-0.19)
Congo	1.19 (1.07,1.30)	1.4 (1.19,1.62)	1.08 (0.97,1.19)	1.28 (1.06,1.49)

Cook Islands	-1.11 (-1.26,-0.96)	-1.65 (-1.93,-1.37)	-1.12 (-1.26,-0.99)	-1.63 (-1.89,-1.37)
Costa Rica	0.52 (0.43,0.61)	0.48 (0.31,0.65)	0.53 (0.45,0.61)	0.43 (0.27,0.58)
Croatia	-0.29 (-0.48,-0.10)	0.56 (0.2,0.92)	-0.47 (-0.65,-0.28)	0.32 (-0.03,0.68)
Cuba	-0.04 (-0.12,0.03)	-0.01 (-0.15,0.14)	-0.14 (-0.22,-0.07)	-0.09 (-0.23,0.05)
Cyprus	-0.44 (-0.56,-0.32)	0.04 (-0.18,0.27)	-0.11 (-0.23,0.00)	0.46 (0.24,0.68)
Czech Republic	-0.66 (-0.86,-0.46)	-0.96 (-1.33,-0.59)	-0.97 (-1.17,-0.78)	-1.24 (-1.61,-0.88)
Democratic People's Republic of Korea	0.40 (0.34,0.46)	0.4 (0.29,0.52)	0.50 (0.42,0.58)	0.48 (0.34,0.63)
Democratic Republic of the Congo	-0.44 (-0.64,-0.25)	-0.41 (-0.77,-0.04)	-0.34 (-0.52,-0.16)	-0.28 (-0.63,0.07)
Denmark	-0.44 (-0.72,-0.16)	-1.23 (-1.76,-0.7)	-0.76 (-1.04,-0.48)	-1.46 (-1.98,-0.94)
Djibouti	0.85 (0.78,0.92)	1.14 (1,1.27)	0.65 (0.58,0.73)	0.93 (0.79,1.08)

Dominica	-0.02 (-0.06,0.03)	-0.01 (-0.09,0.08)	-0.02 (-0.06,0.02)	0.06 (-0.02,0.14)
Dominican Republic	0.29 (0.17,0.41)	0.54 (0.31,0.77)	0.30 (0.20,0.41)	0.52 (0.33,0.71)
East Asia	0.89 (0.80,0.97)	1.15 (0.99,1.31)	0.95 (0.86,1.05)	1.27 (1.09,1.46)
Eastern Europe	0.62 (0.45,0.78)	0.31 (-0.01,0.63)	0.45 (0.27,0.62)	0.08 (-0.26,0.41)
Eastern Sub-Saharan Africa	0.15 (0.11,0.19)	0.09 (0.01,0.16)	0.17 (0.13,0.21)	0.11 (0.03,0.18)
Ecuador	-0.40 (-0.60,-0.21)	0.04 (-0.33,0.42)	-0.58 (-0.78,-0.38)	-0.18 (-0.56,0.2)
Egypt	2.41 (2.21,2.60)	2.92 (2.52,3.31)	2.14 (1.95,2.34)	2.57 (2.19,2.96)
El Salvador	1.37 (1.30,1.44)	1.46 (1.32,1.6)	1.39 (1.33,1.46)	1.5 (1.37,1.63)
Equatorial Guinea	2.52 (2.38,2.66)	3.12 (2.83,3.4)	2.43 (2.29,2.57)	2.98 (2.7,3.26)
Eritrea	0.16 (0.09,0.23)	-0.09 (-0.22,0.04)	0.02 (-0.05,0.09)	-0.25 (-0.39,-0.11)

Estonia	1.39 (1.06,1.72)	1.25 (0.62,1.88)	1.14 (0.80,1.48)	0.92 (0.26,1.59)
Ethiopia	-0.05 (-0.21,0.10)	0.2 (-0.11,0.5)	-0.32 (-0.48,-0.15)	-0.14 (-0.45,0.18)
Federated States of Micronesia	0.29 (0.26,0.32)	0.19 (0.13,0.26)	0.32 (0.29,0.35)	0.25 (0.2,0.31)
Fiji	-2.24 (-2.48,-2.00)	-2.97 (-3.42,-2.53)	-2.32 (-2.55,-2.09)	-3.05 (-3.48,-2.62)
Finland	-1.73 (-1.78,-1.67)	-1.64 (-1.75,-1.54)	-2.03 (-2.08,-1.97)	-1.94 (-2.04,-1.83)
France	-1.24 (-1.29,-1.19)	-0.97 (-1.06,-0.87)	-1.37 (-1.43,-1.32)	-1.06 (-1.17,-0.95)
Gabon	1.64 (1.60,1.67)	1.67 (1.6,1.73)	1.67 (1.63,1.70)	1.73 (1.66,1.8)
Georgia	3.70 (3.41,3.99)	5.05 (4.47,5.64)	3.48 (3.20,3.77)	4.81 (4.24,5.38)
Germany	-1.60 (-1.66,-1.53)	-1.56 (-1.69,-1.44)	-1.97 (-2.03,-1.92)	-1.93 (-2.04,-1.82)
Ghana	2.50 (2.41,2.58)	3.04 (2.87,3.21)	2.53 (2.45,2.62)	3.07 (2.9,3.24)

Greece	0.02 (-0.05,0.10)	-0.2 (-0.34,-0.06)	-0.03 (-0.10,0.04)	-0.16 (-0.28,-0.03)
Greenland	-0.90 (-1.01,-0.80)	-0.84 (-1.05,-0.64)	-1.15 (-1.26,-1.03)	-1.1 (-1.33,-0.88)
Grenada	0.48 (0.24,0.72)	0.29 (-0.17,0.76)	0.30 (0.13,0.47)	0.23 (-0.09,0.55)
Guam	-0.74 (-0.99,-0.48)	-0.25 (-0.74,0.24)	-0.40 (-0.65,-0.16)	0.03 (-0.44,0.5)
Guatemala	-0.28 (-0.43,-0.13)	-0.16 (-0.45,0.13)	-0.17 (-0.33,-0.02)	-0.06 (-0.36,0.24)
Guinea	1.16 (1.13,1.20)	1.26 (1.2,1.32)	1.22 (1.18,1.26)	1.34 (1.26,1.42)
Guinea-Bissau	2.76 (2.65,2.86)	3.28 (3.08,3.49)	2.89 (2.78,3.00)	3.47 (3.25,3.68)
Guyana	-0.24 (-0.35,-0.13)	0.1 (-0.11,0.31)	-0.23 (-0.35,-0.12)	0.11 (-0.11,0.33)
Haiti	-0.98 (-1.02,-0.94)	-0.95 (-1.02,-0.88)	-1.26 (-1.31,-1.21)	-1.27 (-1.37,-1.18)
High SDI	-1.29 (-1.35,-1.23)	-1.34 (-1.44,-1.23)	-1.60 (-1.66,-1.53)	-1.64 (-1.76,-1.51)

High-income	-1.29 (-1.34,-1.24)	-1.3 (-1.4,-1.21)	-1.57 (-1.63,-1.51)	-1.58 (-1.69,-1.46)
High-income Asia Pacific	-1.01 (-1.09,-0.92)	-1.2 (-1.36,-1.04)	-1.40 (-1.50,-1.30)	-1.55 (-1.74,-1.36)
High-income North America	-1.34 (-1.43,-1.25)	-1.46 (-1.63,-1.29)	-1.73 (-1.82,-1.64)	-1.86 (-2.04,-1.69)
High-middle SDI	-0.24 (-0.33,-0.15)	-0.32 (-0.49,-0.15)	-0.40 (-0.49,-0.31)	-0.5 (-0.67,-0.32)
Honduras	0.86 (0.81,0.91)	1.02 (0.92,1.11)	0.77 (0.73,0.81)	0.87 (0.79,0.96)
Hungary	-0.68 (-0.85,-0.52)	-0.74 (-1.05,-0.43)	-0.72 (-0.90,-0.54)	-0.8 (-1.14,-0.45)
Iceland	-1.46 (-1.57,-1.36)	-1.47 (-1.68,-1.27)	-1.56 (-1.66,-1.46)	-1.62 (-1.81,-1.42)
India	0.08 (0.02,0.13)	-0.16 (-0.26,-0.06)	-0.07 (-0.12,-0.02)	-0.29 (-0.39,-0.2)
Indonesia	1.60 (1.52,1.69)	1.55 (1.38,1.72)	1.57 (1.50,1.64)	1.53 (1.39,1.67)
Iraq	0.12 (0.02,0.22)	0.11 (-0.08,0.3)	-0.02 (-0.11,0.07)	-0.05 (-0.22,0.13)

Ireland	-1.92 (-2.07,-1.77)	-1.94 (-2.21,-1.66)	-2.11 (-2.26,-1.95)	-2.12 (-2.41,-1.83)
Islamic Republic of Iran	0.91 (0.84,0.97)	1.38 (1.25,1.51)	0.75 (0.68,0.81)	1.2 (1.08,1.33)
Israel	-1.18 (-1.34,-1.01)	-1.49 (-1.8,-1.18)	-1.33 (-1.51,-1.15)	-1.59 (-1.93,-1.25)
Italy	-1.53 (-1.58,-1.47)	-1.42 (-1.52,-1.31)	-1.83 (-1.89,-1.78)	-1.7 (-1.81,-1.59)
Jamaica	0.04 (-0.19,0.26)	-0.01 (-0.43,0.42)	-0.04 (-0.28,0.21)	-0.15 (-0.62,0.32)
Japan	-1.10 (-1.18,-1.03)	-1.29 (-1.43,-1.15)	-1.47 (-1.55,-1.38)	-1.62 (-1.78,-1.45)
Jordan	0.46 (0.40,0.52)	0.67 (0.55,0.79)	0.41 (0.35,0.47)	0.62 (0.51,0.74)
Kazakhstan	-0.15 (-0.51,0.21)	-0.77 (-1.45,-0.09)	-0.26 (-0.62,0.10)	-0.95 (-1.63,-0.26)
Kenya	0.18 (0.16,0.20)	0.06 (0.02,0.1)	0.36 (0.33,0.38)	0.29 (0.24,0.34)
Kingdom of Eswatini	0.01 (-0.16,0.18)	0.06 (-0.26,0.39)	0.04 (-0.13,0.21)	0.01 (-0.32,0.34)

Kiribati	1.14 (0.98,1.31)	0.93 (0.62,1.24)	1.21 (1.05,1.37)	1.02 (0.71,1.33)
Kuwait	0.79 (0.46,1.11)	0.54 (-0.07,1.17)	0.47 (0.15,0.80)	0.17 (-0.44,0.79)
Kyrgyzstan	3.57 (3.32,3.83)	4.02 (3.52,4.52)	3.45 (3.18,3.71)	3.72 (3.2,4.25)
Lao People's Democratic Republic	0.12 (0.08,0.15)	0.23 (0.16,0.29)	-0.09 (-0.13,-0.06)	0.02 (-0.05,0.08)
Latin America and Caribbean	-0.62 (-0.65,-0.58)	-0.7 (-0.77,-0.63)	-0.79 (-0.83,-0.75)	-0.91 (-0.99,-0.83)
Latvia	2.60 (2.29,2.91)	2.7 (2.09,3.32)	2.50 (2.18,2.82)	2.46 (1.84,3.09)
Lebanon	1.68 (1.47,1.89)	2.31 (1.89,2.73)	1.51 (1.30,1.71)	2.1 (1.7,2.5)
Lesotho	2.32 (2.24,2.41)	2.94 (2.78,3.11)	2.51 (2.42,2.59)	3.1 (2.93,3.28)
Liberia	1.33 (1.21,1.44)	1.82 (1.6,2.04)	1.44 (1.31,1.57)	1.99 (1.74,2.24)
Libya	0.60 (0.52,0.67)	1.02 (0.88,1.16)	0.54 (0.48,0.61)	0.91 (0.79,1.03)

Lithuania	2.34 (2.02,2.65)	2.32 (1.7,2.94)	2.21 (1.88,2.54)	2.13 (1.49,2.77)
Low SDI	-0.14 (-0.17,-0.10)	-0.29 (-0.36,-0.22)	-0.17 (-0.20,-0.13)	-0.35 (-0.41,-0.28)
Low-middle SDI	0.23 (0.21,0.26)	0.21 (0.17,0.26)	0.20 (0.18,0.22)	0.18 (0.14,0.22)
Luxembourg	-1.06 (-1.19,-0.94)	-1.15 (-1.39,-0.92)	-1.35 (-1.48,-1.22)	-1.47 (-1.72,-1.23)
Madagascar	-1.85 (-2.00,-1.71)	-1.83 (-2.1,-1.55)	-1.93 (-2.07,-1.80)	-1.91 (-2.16,-1.66)
Malawi	0.58 (0.53,0.63)	0.51 (0.42,0.61)	0.79 (0.73,0.85)	0.69 (0.56,0.81)
Malaysia	-0.36 (-0.50,-0.22)	-0.86 (-1.12,-0.59)	-0.47 (-0.59,-0.34)	-0.93 (-1.16,-0.69)
Maldives	-1.01 (-1.14,-0.89)	-1.25 (-1.49,-1.02)	-1.04 (-1.18,-0.91)	-1.34 (-1.59,-1.08)
Mali	1.88 (1.82,1.94)	2.19 (2.07,2.3)	1.84 (1.78,1.90)	2.14 (2.01,2.26)
Malta	-1.16 (-1.24,-1.08)	-0.88 (-1.03,-0.72)	-1.00 (-1.09,-0.91)	-0.67 (-0.84,-0.5)

Marshall Islands	0.96 (0.92,1.01)	1.1 (1.01,1.2)	0.78 (0.73,0.83)	0.89 (0.79,0.98)
Mauritania	1.96 (1.92,1.99)	2 (1.94,2.07)	1.87 (1.82,1.91)	1.92 (1.84,2)
Mauritius	0.71 (-0.14,1.56)	-2.06 (-3.63,-0.46)	0.56 (-0.29,1.40)	-2.18 (-3.75,-0.58)
Mexico	-1.55 (-1.64,-1.46)	-1.74 (-1.91,-1.56)	-1.55 (-1.64,-1.45)	-1.78 (-1.96,-1.59)
Middle SDI	0.28 (0.24,0.31)	0.36 (0.29,0.43)	0.22 (0.17,0.26)	0.33 (0.25,0.42)
Mongolia	9.27 (8.57,9.96)	12.35 (10.87,13.86)	9.31 (8.61,10.02)	12.43 (10.92,13.95)
Montenegro	0.73 (0.68,0.78)	0.8 (0.71,0.89)	0.59 (0.54,0.64)	0.69 (0.58,0.79)
Morocco	0.37 (0.25,0.50)	0.32 (0.08,0.56)	0.43 (0.30,0.55)	0.43 (0.2,0.67)
Mozambique	0.32 (0.27,0.37)	0.7 (0.6,0.8)	0.51 (0.44,0.57)	0.92 (0.8,1.04)
Myanmar	-1.94 (-1.98,-1.90)	-2.12 (-2.19,-2.04)	-2.21 (-2.25,-2.16)	-2.42 (-2.51,-2.34)

Namibia	0.09 (-0.04,0.23)	-0.06 (-0.32,0.2)	0.06 (-0.08,0.20)	-0.12 (-0.38,0.15)
Nepal	-0.41 (-0.48,-0.34)	-0.63 (-0.77,-0.5)	-0.55 (-0.63,-0.48)	-0.81 (-0.95,-0.66)
Netherlands	-1.65 (-1.72,-1.58)	-1.55 (-1.69,-1.42)	-2.07 (-2.14,-2.00)	-1.98 (-2.12,-1.84)
New Zealand	-1.22 (-1.28,-1.17)	-1.35 (-1.45,-1.25)	-1.52 (-1.58,-1.46)	-1.69 (-1.8,-1.57)
Nicaragua	0.67 (0.56,0.78)	1.13 (0.91,1.34)	0.67 (0.57,0.77)	1.1 (0.91,1.29)
Niger	1.29 (1.20,1.39)	1.28 (1.09,1.46)	1.11 (1.04,1.17)	1.13 (1.01,1.25)
Nigeria	0.36 (0.32,0.40)	0.49 (0.41,0.57)	0.39 (0.35,0.43)	0.47 (0.4,0.55)
North Africa and Middle East	0.15 (0.11,0.18)	0.25 (0.2,0.29)	0.09 (0.06,0.12)	0.14 (0.09,0.18)
Northern Mariana Islands	0.05 (-0.10,0.21)	-0.01 (-0.31,0.29)	-0.06 (-0.23,0.10)	-0.06 (-0.37,0.25)
Norway	-2.99 (-3.13,-2.85)	-3.23 (-3.48,-2.97)	-3.10 (-3.25,-2.95)	-3.28 (-3.56,-3.01)

Oceania	-0.52 (-0.58,-0.46)	-0.64 (-0.75,-0.52)	-0.46 (-0.52,-0.40)	-0.59 (-0.7,-0.48)
Oman	0.41 (0.30,0.51)	1.02 (0.81,1.22)	0.32 (0.22,0.42)	0.91 (0.71,1.11)
Pakistan	-0.62 (-0.76,-0.49)	-0.86 (-1.12,-0.6)	-0.61 (-0.74,-0.47)	-0.85 (-1.1,-0.59)
Palestine	0.05 (-0.02,0.13)	-0.24 (-0.38,-0.1)	0.14 (0.07,0.21)	-0.14 (-0.28,-0.01)
Panama	-0.32 (-0.41,-0.24)	-0.4 (-0.56,-0.24)	-0.35 (-0.43,-0.27)	-0.43 (-0.59,-0.27)
Papua New Guinea	-0.15 (-0.22,-0.08)	-0.31 (-0.44,-0.17)	-0.09 (-0.16,-0.01)	-0.26 (-0.41,-0.12)
Paraguay	0.28 (0.22,0.35)	0.45 (0.33,0.57)	0.26 (0.19,0.32)	0.34 (0.21,0.47)
Peru	0.79 (0.70,0.89)	0.67 (0.49,0.85)	0.69 (0.60,0.78)	0.5 (0.32,0.68)
Philippines	-0.22 (-0.29,-0.14)	-0.18 (-0.32,-0.05)	-0.14 (-0.22,-0.06)	-0.22 (-0.37,-0.06)
Plurinational State of Bolivia	0.41 (0.34,0.48)	0.81 (0.68,0.95)	0.27 (0.19,0.34)	0.66 (0.53,0.8)

Poland	-1.13 (-1.21,-1.04)	-1.17 (-1.34,-1)	-1.51 (-1.61,-1.40)	-1.54 (-1.73,-1.34)
Portugal	-0.77 (-0.91,-0.62)	-0.72 (-1,-0.45)	-0.73 (-0.88,-0.58)	-0.63 (-0.91,-0.34)
Principality of Monaco	0.44 (0.30,0.57)	0.5 (0.24,0.76)	0.38 (0.24,0.51)	0.43 (0.17,0.7)
Puerto Rico	0.06 (-0.04,0.16)	-0.2 (-0.38,-0.02)	0.04 (-0.06,0.14)	-0.23 (-0.42,-0.04)
Qatar	-0.94 (-1.11,-0.76)	-0.87 (-1.2,-0.54)	-1.01 (-1.16,-0.86)	-0.84 (-1.12,-0.56)
Republic of Cabo Verde	10.59 (9.36,11.82)	9.26 (6.72,11.86)	10.50 (9.28,11.72)	9.17 (6.65,11.75)
Republic of C 么 te d'Ivoire	0.43 (0.32,0.53)	0.13 (-0.07,0.33)	0.48 (0.37,0.59)	0.18 (-0.03,0.38)
Republic of Korea	0.11 (-0.08,0.30)	-0.25 (-0.62,0.12)	-0.47 (-0.67,-0.27)	-0.79 (-1.18,-0.41)
Republic of Moldova	1.09 (0.91,1.28)	1.67 (1.3,2.04)	1.07 (0.89,1.25)	1.66 (1.3,2.01)
Republic of Nauru	-0.83 (-0.90,-0.75)	-1.03 (-1.17,-0.89)	-0.66 (-0.73,-0.59)	-0.87 (-1.01,-0.73)

Republic of Niue	0.68 (0.63,0.73)	0.52 (0.42,0.62)	0.54 (0.50,0.59)	0.36 (0.27,0.45)
Republic of Palau	-0.16 (-0.25,-0.07)	-0.47 (-0.64,-0.29)	-0.18 (-0.28,-0.09)	-0.52 (-0.7,-0.33)
Republic of San Marino	-2.29 (-2.48,-2.10)	-1.31 (-1.66,-0.95)	-2.27 (-2.44,-2.09)	-1.36 (-1.68,-1.03)
Republic of the Gambia	-0.02 (-0.10,0.05)	-0.29 (-0.44,-0.15)	-0.05 (-0.13,0.04)	-0.34 (-0.5,-0.18)
Romania	2.06 (1.90,2.21)	2.07 (1.76,2.37)	2.01 (1.84,2.18)	2.01 (1.68,2.34)
Russian Federation	0.47 (0.32,0.62)	0.19 (-0.1,0.47)	0.27 (0.11,0.43)	-0.07 (-0.38,0.23)
Rwanda	1.24 (1.17,1.32)	1.02 (0.88,1.17)	1.09 (1.01,1.17)	0.83 (0.67,0.98)
Saint Kitts and Nevis	0.00 (-0.09,0.10)	0.26 (0.07,0.45)	-0.25 (-0.35,-0.14)	-0.03 (-0.23,0.16)
Saint Lucia	-0.53 (-0.68,-0.39)	-0.72 (-0.99,-0.44)	-0.69 (-0.82,-0.56)	-0.82 (-1.07,-0.57)
Saint Vincent and the Grenadines	0.23 (0.11,0.36)	0.24 (0,0.48)	0.16 (0.03,0.28)	0.16 (-0.08,0.4)

Samoa	-0.36 (-0.46,-0.27)	-0.78 (-0.96,-0.61)	-0.40 (-0.49,-0.31)	-0.81 (-0.99,-0.64)
Sao Tome and Principe	2.15 (2.05,2.24)	2.22 (2.04,2.41)	2.12 (2.03,2.21)	2.18 (1.99,2.36)
Saudi Arabia	2.73 (2.61,2.86)	2.85 (2.61,3.1)	2.65 (2.52,2.78)	2.8 (2.54,3.06)
Senegal	1.35 (1.31,1.39)	1.4 (1.32,1.48)	1.32 (1.27,1.37)	1.35 (1.26,1.45)
Serbia	0.41 (0.31,0.52)	0.65 (0.44,0.86)	0.54 (0.42,0.66)	0.74 (0.51,0.97)
Seychelles	0.19 (-0.17,0.55)	-0.84 (-1.51,-0.16)	0.02 (-0.34,0.38)	-0.97 (-1.65,-0.29)
Sierra Leone	0.45 (0.40,0.50)	0.71 (0.61,0.81)	0.52 (0.46,0.58)	0.85 (0.73,0.97)
Singapore	-1.66 (-1.88,-1.43)	-1.55 (-1.98,-1.12)	-2.02 (-2.24,-1.80)	-1.86 (-2.27,-1.44)
Slovakia	0.56 (0.45,0.67)	0.49 (0.29,0.69)	0.37 (0.25,0.48)	0.24 (0.02,0.46)
Slovenia	0.66 (0.44,0.87)	1.02 (0.6,1.44)	0.37 (0.14,0.60)	0.77 (0.33,1.22)

Socialist Republic of Viet Nam	1.76 (1.68,1.83)	1.94 (1.8,2.08)	1.86 (1.78,1.94)	2.13 (1.98,2.28)
Solomon Islands	0.16 (0.10,0.22)	0.22 (0.1,0.33)	0.29 (0.22,0.35)	0.36 (0.23,0.49)
Somalia	-0.24 (-0.27,-0.21)	-0.11 (-0.17,-0.06)	-0.17 (-0.19,-0.15)	-0.07 (-0.11,-0.04)
South Africa	-1.34 (-1.38,-1.30)	-1.3 (-1.38,-1.23)	-1.32 (-1.37,-1.28)	-1.25 (-1.33,-1.16)
South Asia	-0.14 (-0.19,-0.09)	-0.51 (-0.58,-0.45)	-0.17 (-0.21,-0.13)	-0.56 (-0.62,-0.5)
South Sudan	0.11 (0.06,0.17)	0.1 (-0.01,0.21)	0.03 (-0.05,0.11)	0 (-0.15,0.15)
Southeast Asia	0.17 (0.12,0.23)	-0.07 (-0.17,0.04)	0.17 (0.12,0.23)	-0.05 (-0.16,0.05)
Southeast Asia, East Asia, and Oceania	0.84 (0.77,0.91)	1.04 (0.9,1.18)	0.85 (0.76,0.93)	1.1 (0.94,1.25)
Southern Latin America	-0.01 (-0.12,0.10)	0.1 (-0.11,0.32)	-0.21 (-0.31,-0.11)	-0.12 (-0.32,0.08)
Southern Sub-Saharan Africa	-0.94 (-0.99,-0.88)	-0.86 (-0.96,-0.76)	-0.89 (-0.95,-0.84)	-0.79 (-0.89,-0.69)

Spain	-0.41 (-0.48,-0.35)	-0.31 (-0.43,-0.18)	-0.51 (-0.59,-0.42)	-0.4 (-0.56,-0.23)
Sri Lanka	-5.90 (-6.51,-5.29)	-8.21 (-9.28,-7.12)	-5.96 (-6.60,-5.32)	-8.38 (-9.5,-7.25)
Sub-Saharan Africa	-0.23 (-0.24,-0.21)	-0.23 (-0.25,-0.2)	-0.16 (-0.17,-0.15)	-0.15 (-0.18,-0.12)
Sudan	0.81 (0.78,0.85)	0.84 (0.78,0.9)	0.71 (0.68,0.74)	0.71 (0.66,0.77)
Suriname	-0.49 (-0.60,-0.38)	-0.24 (-0.45,-0.04)	-0.48 (-0.59,-0.36)	-0.25 (-0.46,-0.03)
Sweden	-2.25 (-2.34,-2.16)	-2.01 (-2.19,-1.84)	-2.61 (-2.71,-2.50)	-2.29 (-2.49,-2.1)
Switzerland	-0.20 (-0.40,0.00)	-0.34 (-0.72,0.05)	-0.63 (-0.84,-0.43)	-0.84 (-1.23,-0.45)
Syrian Arab Republic	0.01 (-0.05,0.07)	-0.07 (-0.18,0.05)	-0.15 (-0.20,-0.11)	-0.24 (-0.33,-0.15)
Taiwan (Province of China)	2.03 (1.66,2.40)	1.34 (0.63,2.05)	1.75 (1.39,2.10)	1.02 (0.34,1.7)
Tajikistan	-2.19 (-2.31,-2.07)	-2.34 (-2.56,-2.12)	-2.18 (-2.28,-2.08)	-2.35 (-2.54,-2.17)

Thailand	-0.53 (-0.57,-0.48)	-0.75 (-0.83,-0.67)	-0.53 (-0.57,-0.49)	-0.7 (-0.77,-0.62)
The former Yugoslav Republic of Macedonia	1.46 (1.25,1.67)	1.7 (1.3,2.11)	1.40 (1.20,1.59)	1.66 (1.28,2.04)
Timor-Leste	-0.01 (-0.05,0.02)	0.01 (-0.06,0.08)	0.01 (-0.05,0.07)	0.05 (-0.06,0.16)
Togo	0.89 (0.88,0.91)	0.82 (0.78,0.85)	1.05 (1.03,1.06)	0.98 (0.95,1.02)
Tokelau	0.16 (0.12,0.19)	0.03 (-0.04,0.1)	0.20 (0.17,0.23)	0.09 (0.02,0.15)
Tonga	0.26 (0.18,0.34)	0 (-0.15,0.16)	0.30 (0.23,0.37)	0.07 (-0.07,0.21)
Trinidad and Tobago	-0.94 (-1.03,-0.85)	-0.95 (-1.12,-0.78)	-1.00 (-1.09,-0.90)	-1.01 (-1.19,-0.84)
Tropical Latin America	-0.43 (-0.47,-0.39)	-0.5 (-0.58,-0.43)	-0.66 (-0.71,-0.61)	-0.79 (-0.88,-0.7)
Tunisia	0.30 (0.27,0.32)	0.24 (0.18,0.29)	0.33 (0.31,0.35)	0.28 (0.24,0.32)
Turkey	-0.74 (-0.83,-0.65)	-1.13 (-1.3,-0.95)	-0.77 (-0.84,-0.69)	-1.11 (-1.25,-0.96)

Turkmenistan	5.51 (4.79,6.24)	7.03 (5.55,8.53)	5.39 (4.65,6.12)	6.86 (5.37,8.38)
Tuvalu	0.62 (0.58,0.66)	0.54 (0.46,0.61)	0.56 (0.53,0.58)	0.48 (0.43,0.53)
Uganda	0.91 (0.73,1.10)	0.52 (0.17,0.87)	0.97 (0.78,1.15)	0.52 (0.16,0.88)
Ukraine	0.30 (0.10,0.51)	-0.16 (-0.56,0.24)	0.25 (0.03,0.47)	-0.28 (-0.7,0.14)
United Arab Emirates	0.65 (0.41,0.88)	2.2 (1.73,2.66)	0.02 (-0.18,0.23)	1.24 (0.84,1.64)
United Kingdom of Great Britain and Northern Ireland	-1.03 (-1.09,-0.97)	-1.02 (-1.14,-0.91)	-1.41 (-1.48,-1.33)	-1.4 (-1.54,-1.26)
United Republic of Tanzania	-0.16 (-0.21,-0.11)	-0.43 (-0.53,-0.34)	-0.14 (-0.19,-0.10)	-0.4 (-0.49,-0.31)
United States of America	-1.35 (-1.45,-1.26)	-1.5 (-1.68,-1.33)	-1.75 (-1.84,-1.65)	-1.91 (-2.09,-1.73)
United States Virgin Islands	-1.64 (-1.84,-1.44)	-1.87 (-2.25,-1.49)	-1.76 (-1.95,-1.56)	-1.94 (-2.3,-1.58)
Uruguay	0.90 (0.80,0.99)	0.65 (0.46,0.84)	0.75 (0.66,0.85)	0.47 (0.29,0.65)

Uzbekistan	3.12 (2.93,3.32)	3.14 (2.76,3.53)	2.85 (2.65,3.05)	2.79 (2.4,3.19)
Vanuatu	-0.93 (-0.97,-0.89)	-1.04 (-1.11,-0.96)	-0.92 (-0.96,-0.88)	-1.04 (-1.11,-0.97)
Western Europe	-1.32 (-1.36,-1.29)	-1.26 (-1.32,-1.19)	-1.59 (-1.64,-1.54)	-1.51 (-1.59,-1.42)
Western Sub-Saharan Africa	0.96 (0.93,0.99)	1.07 (1.01,1.13)	1.02 (0.99,1.05)	1.1 (1.04,1.17)
Yemen	0.37 (0.33,0.40)	0.48 (0.42,0.54)	0.16 (0.13,0.19)	0.25 (0.19,0.31)
Zambia	1.80 (1.67,1.92)	2.1 (1.86,2.33)	2.00 (1.86,2.14)	2.34 (2.07,2.61)
Zimbabwe	0.74 (0.44,1.05)	1.1 (0.51,1.69)	0.83 (0.52,1.14)	1.12 (0.52,1.72)

AAPC, average annual percentage change; EAPC, estimated annual percentage change; ASMR, age-standardized mortality rates; ASDR, age-standardized disability-adjusted life year rates; CI, confidence interval.

Supplementary Table 7. The global burden of kidney cancer attributable to smoking in 2021.

location_name	Deaths cases (95% UI)	ASMR (95% UI)	PAF of ASM (95% UI)	DALYs cases (95% UI)	ASDR (95% UI)	PAF of ADM (95% UI)
Afghanistan	4 (2,7)	0.05 (0.03,0.09)	4.3 (2.18,7.25)	120 (59,206)	1.19 (0.6,1.99)	3.55 (1.78,5.98)

Albania	17 (10,26)	0.38 (0.23,0.58)	17.81 (11.37,24.78)	377 (219,573)	8.45 (4.92,12.83)	16.25 (10.51,22.27)
Algeria	20 (11,32)	0.06 (0.04,0.1)	11.28 (6.7,16.73)	466 (263,735)	1.32 (0.75,2.12)	9.88 (5.96,14.43)
American Samoa	0 (0,0)	0.07 (0.04,0.11)	7.58 (4.44,11.45)	1 (0,1)	1.64 (0.91,2.61)	6.8 (3.86,10.12)
Andean Latin America	31 (17,51)	0.05 (0.03,0.09)	2.59 (1.41,4.12)	666 (371,1058)	1.14 (0.63,1.82)	2.05 (1.17,3.15)
Andorra	0 (0,1)	0.26 (0.12,0.44)	11.22 (6.47,17.02)	9 (5,16)	6.13 (3.02,10.66)	11.1 (6.39,16.67)
Angola	5 (2,9)	0.04 (0.02,0.08)	5.22 (2.87,7.97)	138 (59,259)	1.08 (0.46,2.01)	4.64 (2.53,7.12)
Antigua and Barbuda	0 (0,0)	0.1 (0.05,0.16)	5.74 (3.01,9.14)	3 (1,4)	2.3 (1.22,3.5)	4.88 (2.68,7.59)
Argentina	249 (146,375)	0.45 (0.26,0.67)	9.6 (5.63,13.99)	6396 (3757,9421)	11.78 (6.95,17.32)	9.39 (5.7,13.59)
Armenia	19 (12,28)	0.43 (0.26,0.63)	15.03 (10.09,19.99)	473 (291,688)	10.77 (6.62,15.64)	14.77 (9.85,19.63)
Australasia	110 (58,181)	0.2 (0.11,0.33)	7 (3.75,11.22)	2403 (1339,3786)	4.76 (2.69,7.4)	7.04 (3.99,10.79)
Australia	86 (46,145)	0.19 (0.1,0.31)	6.63 (3.56,10.67)	1905 (1057,3039)	4.53 (2.56,7.13)	6.83 (3.85,10.47)
Austria	60 (35,89)	0.33 (0.2,0.48)	10.58 (6.36,15.27)	1315 (802,1904)	7.88 (4.84,11.26)	11.4 (7.04,15.98)

Azerbaijan	43 (22,72)	0.39 (0.2,0.67)	13.38 (8.28,18.62)	1263 (658,2123)	10.56 (5.56,17.85)	12.34 (7.73,17.02)
Bahrain	2 (1,3)	0.26 (0.15,0.39)	10.3 (6.17,14.86)	57 (32,90)	5.7 (3.23,8.81)	9.92 (6.01,14.51)
Bangladesh	70 (37,114)	0.05 (0.03,0.09)	9.01 (5.57,12.83)	1563 (812,2502)	1.14 (0.6,1.83)	7.04 (4.43,10.06)
Barbados	1 (0,1)	0.11 (0.05,0.18)	4.19 (2.22,6.78)	12 (6,20)	2.3 (1.15,3.71)	3.27 (1.81,5.13)
Belarus	95 (58,140)	0.58 (0.35,0.85)	14.32 (9.29,19.43)	2546 (1559,3724)	15.76 (9.62,23.1)	14.37 (9.29,19.47)
Belgium	80 (45,124)	0.33 (0.19,0.51)	10.98 (6.35,16.31)	1684 (977,2558)	7.78 (4.56,11.68)	11.28 (6.74,16.34)
Belize	0 (0,0)	0.07 (0.04,0.11)	4.53 (2.48,6.79)	5 (3,8)	1.73 (0.92,2.65)	3.52 (1.98,5.23)
Benin	0 (0,1)	0.01 (0,0.01)	1.11 (0.65,1.71)	9 (5,13)	0.17 (0.09,0.26)	0.74 (0.42,1.16)
Bermuda	0 (0,0)	0.15 (0.08,0.24)	6.79 (3.56,11.06)	5 (2,7)	3.45 (1.82,5.56)	5.8 (3.15,9.13)
Bhutan	0 (0,0)	0.04 (0.02,0.08)	4.99 (2.46,8.43)	5 (2,10)	0.84 (0.36,1.66)	3.93 (1.93,6.68)
Bolivarian Republic of Venezuela	31 (16,49)	0.1 (0.05,0.16)	3.8 (2.25,5.78)	738 (389,1179)	2.38 (1.25,3.8)	3.11 (1.86,4.69)
Bosnia and Herzegovina	34 (20,50)	0.53 (0.32,0.78)	14.88 (9.66,20.6)	821 (486,1211)	13.28 (7.86,19.6)	15.1 (9.96,20.7)

Botswana	1 (0,1)	0.07 (0.04,0.11)	5.47 (3.25,8)	23 (11,38)	1.57 (0.81,2.51)	4.31 (2.51,6.25)
Brazil	435 (244,672)	0.17 (0.1,0.27)	8.62 (4.68,13.49)	10328 (5901,15507)	4.04 (2.3,6.07)	7.46 (4.16,11.45)
Brunei Darussalam	1 (0,1)	0.25 (0.14,0.38)	9.05 (5.12,13.49)	22 (13,33)	5.78 (3.27,8.91)	8.86 (5.15,13.2)
Bulgaria	55 (32,82)	0.41 (0.24,0.61)	13.15 (8.21,18.27)	1487 (871,2194)	11.85 (7,17.47)	14.24 (8.99,19.75)
Burkina Faso	0 (0,1)	0.01 (0,0.01)	0.91 (0.49,1.51)	13 (6,21)	0.13 (0.06,0.22)	0.64 (0.34,1.03)
Burundi	1 (0,1)	0.01 (0.01,0.03)	1.4 (0.8,2.18)	16 (6,29)	0.32 (0.12,0.58)	1.14 (0.65,1.85)
Cambodia	15 (9,22)	0.13 (0.08,0.18)	13.02 (8.44,17.54)	398 (235,572)	3.07 (1.83,4.37)	10.56 (6.84,14.36)
Cameroon	2 (1,3)	0.01 (0.01,0.03)	1.78 (1.02,2.78)	53 (26,98)	0.38 (0.19,0.71)	1.41 (0.8,2.22)
Canada	237 (128,374)	0.32 (0.17,0.5)	10.69 (5.82,16.58)	5124 (2909,7963)	7.31 (4.16,11.23)	10.64 (6.03,16.14)
Caribbean	67 (39,101)	0.12 (0.07,0.19)	7.42 (4.37,11.1)	1598 (931,2361)	2.95 (1.72,4.36)	6.07 (3.65,8.85)
Central African Republic	0 (0,1)	0.02 (0.01,0.03)	2.32 (1.31,3.59)	9 (4,16)	0.38 (0.17,0.71)	1.96 (1.11,3.04)
Central Asia	186 (119,262)	0.22 (0.14,0.31)	9.76 (6.28,13.45)	5299 (3398,7461)	5.84 (3.75,8.25)	9.15 (5.91,12.56)

Central Europe	1091 (657,1590)	0.49 (0.3,0.71)	11.57 (7.15,16.39)	26128 (16114,37549)	12.35 (7.66,17.67)	12.01 (7.55,16.62)
Central Europe, Eastern Europe, and Central Asia	2935 (1838,4087)	0.44 (0.28,0.62)	11.4 (7.17,15.83)	76066 (47931,104873)	11.72 (7.4,16.13)	11.7 (7.45,16.11)
Central Latin America	186 (108,278)	0.08 (0.04,0.11)	3.09 (1.82,4.57)	4249 (2514,6243)	1.69 (1,2.48)	2.51 (1.49,3.63)
Central Sub-Saharan Africa	9 (4,17)	0.02 (0.01,0.03)	2.41 (1.43,3.68)	256 (115,470)	0.44 (0.19,0.8)	2.12 (1.27,3.18)
Chad	0 (0,1)	0.01 (0,0.02)	1.72 (0.89,3.02)	13 (6,25)	0.21 (0.1,0.41)	1.16 (0.58,2.07)
Chile	58 (31,91)	0.22 (0.12,0.35)	5.66 (3.04,8.83)	1425 (807,2198)	5.57 (3.17,8.57)	5.56 (3.18,8.39)
China	3481 (2068,4957)	0.16 (0.1,0.23)	13.19 (8.24,17.74)	87271 (51675,124166)	3.98 (2.36,5.66)	11.62 (7.5,15.58)
Colombia	23 (12,37)	0.04 (0.02,0.07)	2.79 (1.57,4.38)	514 (281,825)	0.93 (0.51,1.49)	2.32 (1.34,3.57)
Commonwealth of the Bahamas	0 (0,1)	0.11 (0.06,0.19)	5.17 (2.65,8.32)	12 (6,20)	2.77 (1.43,4.64)	4.22 (2.26,6.69)
Comoros	0 (0,0)	0.04 (0.02,0.07)	2.8 (1.5,4.69)	4 (2,8)	0.85 (0.34,1.57)	2.04 (1.05,3.46)
Congo	1 (0,2)	0.04 (0.02,0.07)	3.68 (2.11,5.64)	24 (12,40)	0.9 (0.46,1.54)	3 (1.79,4.55)
Cook Islands	0 (0,0)	0.02 (0.01,0.03)	6.46 (3.55,10.22)	0 (0,0)	0.36 (0.19,0.6)	5.64 (3.22,9.15)

Costa Rica	8 (5,13)	0.15 (0.08,0.24)	6.1 (3.37,9.3)	196 (107,304)	3.53 (1.93,5.48)	5.15 (2.88,7.73)
Croatia	54 (31,81)	0.58 (0.34,0.88)	14.23 (8.95,20.28)	1189 (708,1775)	13.96 (8.38,20.79)	14.27 (9.07,19.96)
Cuba	38 (22,56)	0.19 (0.11,0.28)	10.19 (6.15,14.6)	921 (544,1355)	4.73 (2.8,6.89)	9.35 (5.81,13.07)
Cyprus	6 (3,9)	0.26 (0.14,0.41)	13.71 (8.02,20.2)	123 (69,189)	5.96 (3.37,9.14)	14.47 (8.8,20.9)
Czech Republic	152 (87,231)	0.69 (0.4,1.04)	11.6 (6.89,16.82)	3431 (2032,5142)	16.69 (10.1,25.07)	11.95 (7.29,17.16)
Democratic People's Republic of Korea	28 (16,42)	0.08 (0.05,0.12)	9 (5.72,12.53)	820 (464,1206)	2.33 (1.32,3.42)	8.07 (5.12,11.44)
Democratic Republic of the Congo	3 (1,5)	0.01 (0,0.01)	1.12 (0.66,1.7)	72 (29,143)	0.18 (0.08,0.36)	1 (0.6,1.5)
Denmark	60 (35,92)	0.47 (0.28,0.73)	14.27 (8.4,21)	1189 (708,1787)	10.17 (6.07,14.95)	13.96 (8.43,20.07)
Djibouti	0 (0,1)	0.06 (0.02,0.12)	4.36 (2.31,7.18)	8 (3,16)	1.36 (0.51,2.6)	3.32 (1.77,5.58)
Dominica	0 (0,0)	0.09 (0.05,0.16)	3.97 (2.07,6.45)	2 (1,3)	2.1 (1.09,3.69)	3.14 (1.72,5.02)
Dominican Republic	8 (5,14)	0.08 (0.05,0.15)	7.84 (4.69,11.77)	194 (108,334)	1.94 (1.07,3.34)	5.58 (3.35,8.34)
East Asia	3614 (2156,5143)	0.17 (0.1,0.23)	12.95 (8.11,17.37)	90535 (53869,127788)	3.99 (2.37,5.65)	11.45 (7.41,15.34)

Eastern Europe	1659 (1053,2309)	0.46 (0.29,0.65)	11.67 (7.51,16.21)	44638 (28604,61835)	12.81 (8.22,17.74)	12.11 (7.88,16.63)
Eastern Sub-Saharan Africa	33 (16,51)	0.02 (0.01,0.03)	1.68 (1.02,2.45)	823 (403,1302)	0.49 (0.25,0.77)	1.31 (0.8,1.89)
Ecuador	8 (5,13)	0.05 (0.03,0.08)	3.07 (1.69,4.83)	181 (102,289)	1.11 (0.63,1.77)	2.44 (1.39,3.75)
Egypt	62 (38,89)	0.11 (0.07,0.16)	11.83 (7.52,16.23)	1700 (1028,2434)	2.53 (1.54,3.63)	10.77 (6.89,14.79)
El Salvador	3 (2,5)	0.05 (0.03,0.08)	3.45 (1.84,5.47)	73 (39,117)	1.22 (0.66,1.95)	3.08 (1.72,4.76)
Equatorial Guinea	0 (0,0)	0.03 (0.01,0.05)	2.35 (1.07,4.19)	3 (1,6)	0.66 (0.29,1.21)	2.07 (0.95,3.71)
Eritrea	0 (0,1)	0.01 (0,0.03)	1.29 (0.57,2.42)	12 (4,27)	0.38 (0.13,0.82)	1.12 (0.5,2.13)
Estonia	13 (7,19)	0.48 (0.28,0.72)	10.37 (6.24,15.32)	299 (177,447)	12.56 (7.53,18.52)	11.2 (6.94,15.98)
Ethiopia	5 (2,8)	0.01 (0.01,0.02)	0.85 (0.51,1.3)	112 (48,189)	0.27 (0.12,0.45)	0.69 (0.4,1.08)
Federated States of Micronesia	0 (0,0)	0.04 (0.02,0.07)	7.1 (4.28,10.38)	1 (0,1)	1.08 (0.53,1.69)	6.4 (3.86,9.29)
Fiji	0 (0,0)	0.01 (0.01,0.02)	3.87 (2.24,5.87)	2 (1,4)	0.3 (0.17,0.49)	3.21 (1.93,4.73)
Finland	33 (18,55)	0.26 (0.14,0.42)	7.72 (4.1,12.18)	718 (392,1130)	6.24 (3.47,9.62)	7.98 (4.48,12.14)

France	484 (270,748)	0.33 (0.19,0.51)	9.62 (5.47,14.42)	10025 (5831,15036)	7.76 (4.6,11.47)	9.31 (5.5,13.5)
Gabon	0 (0,1)	0.03 (0.02,0.06)	2.46 (1.38,3.85)	9 (5,15)	0.8 (0.42,1.34)	2.17 (1.25,3.37)
Georgia	29 (18,41)	0.48 (0.3,0.7)	15.45 (10.19,20.77)	759 (477,1107)	13.3 (8.38,19.34)	15.25 (10.21,20.3)
Germany	809 (452,1233)	0.4 (0.23,0.6)	11.05 (6.34,16.57)	16487 (9598,24283)	9.12 (5.39,13.18)	11.33 (6.68,16.52)
Ghana	1 (1,2)	0.01 (0,0.01)	1.19 (0.66,1.91)	29 (17,49)	0.18 (0.1,0.3)	0.81 (0.44,1.32)
Global	16216 (9663,23217)	0.19 (0.11,0.27)	9.87 (5.92,14.1)	382927 (233635,536755)	4.37 (2.66,6.14)	9.23 (5.72,13.01)
Greece	120 (73,175)	0.5 (0.31,0.71)	15.73 (9.78,21.97)	2494 (1536,3501)	11.96 (7.48,16.56)	16.04 (10.15,22.23)
Greenland	0 (0,1)	0.68 (0.36,1.08)	13.02 (7.88,19.02)	13 (7,20)	16.46 (8.94,26)	12.51 (7.46,18.36)
Grenada	0 (0,0)	0.08 (0.04,0.13)	3.9 (2.07,6.36)	2 (1,4)	1.94 (1.04,3.12)	3.25 (1.8,5.11)
Guam	0 (0,0)	0.07 (0.04,0.11)	5.8 (3.42,9.14)	4 (2,7)	1.92 (1.07,3.1)	5.09 (3.03,7.87)
Guatemala	3 (2,5)	0.03 (0.02,0.05)	2.19 (1.21,3.39)	75 (42,115)	0.68 (0.38,1.05)	1.7 (0.96,2.61)
Guinea	1 (0,1)	0.01 (0.01,0.02)	2.64 (1.51,4.04)	20 (11,31)	0.35 (0.19,0.55)	1.84 (0.98,2.97)

Guinea-Bissau	0 (0,0)	0.01 (0.01,0.02)	1.62 (0.85,2.71)	2 (1,3)	0.26 (0.13,0.44)	1.29 (0.67,2.16)
Guyana	0 (0,1)	0.06 (0.03,0.11)	3.5 (1.92,5.5)	11 (5,18)	1.57 (0.78,2.6)	2.8 (1.58,4.32)
Haiti	1 (1,3)	0.02 (0.01,0.05)	1.75 (0.84,3.13)	39 (16,76)	0.53 (0.21,1.04)	1.27 (0.61,2.15)
High SDI	7046 (4030,10669)	0.33 (0.19,0.49)	10.98 (6.27,16.42)	152961 (89126,226095)	7.68 (4.54,11.29)	10.98 (6.51,16.12)
High-income	7247 (4066,11086)	0.32 (0.19,0.48)	10.81 (6.11,16.24)	156081 (90501,232106)	7.63 (4.47,11.22)	10.76 (6.33,15.89)
High-income Asia Pacific	980 (574,1443)	0.2 (0.12,0.28)	10.34 (6.17,14.95)	18364 (11191,26517)	4.26 (2.63,6.08)	10.33 (6.32,14.74)
High-income North America	2365 (1276,3741)	0.35 (0.19,0.55)	11.6 (6.29,18.01)	55145 (31522,84516)	8.66 (5.04,13.2)	11.63 (6.69,17.62)
High-middle SDI	5509 (3390,7688)	0.27 (0.17,0.38)	11.41 (7.14,15.7)	138425 (86981,191405)	6.88 (4.32,9.5)	11.24 (7.19,15.45)
Honduras	3 (2,5)	0.05 (0.03,0.09)	5.3 (3.17,8)	81 (45,132)	1.24 (0.69,2.04)	4.6 (2.75,7.01)
Hungary	91 (53,137)	0.49 (0.29,0.74)	11.28 (6.95,16.23)	2344 (1411,3491)	13.57 (8.26,20.06)	11.96 (7.49,16.85)
Iceland	3 (2,4)	0.48 (0.26,0.75)	10.23 (5.75,15.69)	63 (35,95)	11.37 (6.3,16.99)	10.47 (6.13,15.62)
India	373 (229,542)	0.03 (0.02,0.05)	4.83 (2.96,6.84)	8861 (5386,12708)	0.74 (0.45,1.07)	4.01 (2.53,5.72)

Indonesia	170 (100,252)	0.08 (0.05,0.11)	7.61 (4.73,10.91)	4580 (2672,6875)	1.78 (1.04,2.65)	6.16 (3.86,8.89)
Iraq	51 (29,79)	0.23 (0.13,0.36)	14.14 (8.88,19.98)	1390 (795,2194)	5.47 (3.15,8.57)	12.52 (8.12,17.75)
Ireland	24 (13,38)	0.29 (0.16,0.46)	10.01 (5.62,15.31)	500 (284,778)	6.36 (3.64,9.85)	9.53 (5.56,14.31)
Islamic Republic of Iran	64 (39,98)	0.09 (0.05,0.13)	7.44 (4.46,11.09)	1682 (1031,2479)	2.08 (1.25,3.11)	6.75 (4.19,9.84)
Israel	31 (18,49)	0.25 (0.14,0.38)	9.7 (5.49,14.8)	669 (391,1018)	5.61 (3.31,8.45)	9.91 (5.9,14.67)
Italy	480 (268,737)	0.32 (0.18,0.48)	10.25 (6.03,15.26)	9852 (5764,14394)	7.43 (4.49,10.68)	10.26 (6.2,14.79)
Jamaica	3 (2,5)	0.1 (0.05,0.16)	6.3 (3.44,9.66)	70 (37,116)	2.3 (1.2,3.79)	5.24 (2.93,7.91)
Japan	778 (452,1152)	0.2 (0.12,0.29)	10.13 (6.02,14.57)	14025 (8408,20228)	4.34 (2.67,6.18)	10.21 (6.27,14.47)
Jordan	13 (8,20)	0.18 (0.11,0.27)	15.69 (10.11,21.72)	369 (220,567)	4.44 (2.63,6.76)	14.57 (9.53,20.04)
Kazakhstan	38 (23,54)	0.2 (0.12,0.28)	7.65 (4.9,10.56)	1110 (679,1590)	5.53 (3.39,7.94)	7.56 (4.93,10.44)
Kenya	3 (1,4)	0.01 (0.01,0.02)	1.75 (1.05,2.6)	69 (38,106)	0.3 (0.16,0.46)	1.5 (0.89,2.2)
Kingdom of Eswatini	0 (0,0)	0.03 (0.01,0.05)	1.45 (0.83,2.26)	3 (1,6)	0.56 (0.26,1.07)	1.08 (0.61,1.64)

Kiribati	0 (0,0)	0.17 (0.1,0.27)	13.15 (8.53,18.23)	3 (2,5)	4.23 (2.36,6.83)	10.96 (7.16,15.12)
Kuwait	4 (2,6)	0.15 (0.08,0.23)	10.81 (6.47,15.8)	112 (66,166)	3.4 (1.97,5.19)	9.76 (5.92,14.18)
Kyrgyzstan	13 (8,19)	0.26 (0.16,0.38)	12.14 (8.01,16.32)	387 (242,568)	7.06 (4.46,10.35)	11.51 (7.64,15.59)
Lao People's Democratic Republic	4 (2,6)	0.1 (0.06,0.14)	10.42 (6.58,14.47)	105 (62,161)	2.23 (1.31,3.33)	8.13 (5.05,11.45)
Latin America and Caribbean	729 (408,1095)	0.12 (0.07,0.18)	5.48 (3.1,8.39)	17068 (9809,25050)	2.72 (1.56,4)	4.6 (2.67,6.97)
Latvia	20 (12,29)	0.52 (0.32,0.77)	10.01 (6.3,14.1)	496 (306,725)	14.18 (8.73,20.63)	10.5 (6.76,14.59)
Lebanon	18 (11,27)	0.29 (0.17,0.45)	16.77 (10.79,23.22)	392 (237,591)	6.68 (4.03,10.02)	16.08 (10.51,22.23)
Lesotho	1 (1,2)	0.1 (0.06,0.16)	7.6 (4.63,11.01)	29 (16,47)	2.56 (1.44,4.11)	6.57 (3.98,9.55)
Liberia	0 (0,0)	0.01 (0,0.01)	1.37 (0.74,2.24)	5 (3,8)	0.21 (0.11,0.35)	1.05 (0.59,1.7)
Libya	16 (9,27)	0.32 (0.18,0.53)	11.97 (7.53,16.74)	452 (249,734)	7.95 (4.41,12.99)	11.17 (7.03,15.38)
Lithuania	29 (17,42)	0.5 (0.31,0.74)	9.61 (5.87,13.92)	679 (421,989)	12.97 (8.15,18.87)	9.59 (6,13.59)
Low SDI	101 (56,151)	0.02 (0.01,0.03)	2.63 (1.63,3.75)	2536 (1407,3762)	0.5 (0.28,0.75)	1.94 (1.2,2.78)

Low-middle SDI	735 (455,1050)	0.05 (0.03,0.08)	6.32 (3.96,8.93)	18224 (11317,25873)	1.25 (0.77,1.77)	5.1 (3.24,7.08)
Luxembourg	2 (1,3)	0.19 (0.11,0.29)	11.05 (6.17,16.55)	46 (25,69)	4.47 (2.44,6.67)	11.3 (6.35,16.82)
Madagascar	1 (0,1)	0.01 (0,0.02)	0.97 (0.53,1.51)	20 (8,37)	0.18 (0.07,0.33)	0.69 (0.38,1.06)
Malawi	5 (2,8)	0.07 (0.04,0.12)	3.26 (1.79,5.03)	116 (56,197)	1.6 (0.79,2.69)	2.35 (1.25,3.69)
Malaysia	30 (19,44)	0.11 (0.07,0.16)	7.98 (4.99,11.61)	723 (446,1060)	2.5 (1.54,3.66)	7.18 (4.62,10.33)
Maldives	0 (0,0)	0.09 (0.05,0.12)	16.51 (10.96,22.54)	6 (3,8)	1.74 (1.06,2.6)	12.84 (8.36,17.51)
Mali	1 (0,1)	0.01 (0.01,0.02)	1.73 (1.05,2.72)	21 (12,33)	0.24 (0.14,0.39)	1.12 (0.61,1.76)
Malta	3 (1,4)	0.28 (0.16,0.43)	9.92 (5.71,14.95)	62 (35,94)	7.44 (4.22,11.02)	10.66 (6.35,15.24)
Marshall Islands	0 (0,0)	0.04 (0.02,0.08)	7 (3.7,11.26)	0 (0,1)	0.94 (0.4,1.85)	6.16 (3.31,10.04)
Mauritania	0 (0,1)	0.02 (0.01,0.02)	2.03 (1.21,3)	9 (4,14)	0.38 (0.19,0.62)	1.67 (0.98,2.47)
Mauritius	1 (1,2)	0.08 (0.05,0.11)	7.76 (4.95,10.9)	34 (21,49)	1.78 (1.11,2.52)	6.48 (4.12,9.08)
Mexico	108 (63,162)	0.09 (0.05,0.13)	2.87 (1.68,4.26)	2419 (1416,3649)	1.91 (1.11,2.87)	2.28 (1.34,3.34)

Middle SDI	2802 (1718,3871)	0.11 (0.06,0.15)	8.7 (5.4,12.02)	70248 (43364,97386)	2.51 (1.55,3.48)	7.49 (4.69,10.28)
Mongolia	4 (2,6)	0.18 (0.1,0.26)	6.89 (4.32,9.81)	118 (67,176)	4.62 (2.61,6.87)	6.32 (3.96,8.85)
Montenegro	5 (3,8)	0.53 (0.32,0.75)	14.89 (9.49,20.81)	138 (85,200)	13.7 (8.34,19.98)	15.97 (10.23,22.08)
Morocco	5 (3,9)	0.02 (0.01,0.02)	5.76 (3.36,8.7)	146 (81,231)	0.4 (0.22,0.62)	5.61 (3.33,8.3)
Mozambique	1 (1,2)	0.01 (0.01,0.02)	1.47 (0.88,2.24)	25 (14,39)	0.23 (0.13,0.36)	1.18 (0.69,1.85)
Myanmar	20 (11,31)	0.04 (0.03,0.07)	5.31 (3.22,7.8)	462 (262,712)	0.95 (0.54,1.47)	3.71 (2.24,5.45)
Namibia	0 (0,1)	0.04 (0.03,0.06)	2.48 (1.55,3.61)	11 (6,16)	0.84 (0.49,1.26)	1.77 (1.12,2.5)
Nepal	6 (3,10)	0.03 (0.02,0.05)	4.48 (2.79,6.38)	127 (67,219)	0.57 (0.3,0.97)	3.34 (2.05,4.77)
Netherlands	170 (93,264)	0.46 (0.25,0.7)	12.26 (7.01,18.5)	3422 (1920,5234)	9.75 (5.56,14.91)	11.59 (6.78,17.21)
New Zealand	23 (12,37)	0.27 (0.14,0.42)	8.94 (4.82,14.28)	498 (270,761)	5.99 (3.26,9.11)	8.08 (4.51,12.54)
Nicaragua	2 (1,4)	0.05 (0.03,0.08)	4.3 (2.55,6.46)	61 (35,94)	1.23 (0.72,1.91)	3.6 (2.16,5.33)
Niger	0 (0,1)	0 (0,0.01)	1.01 (0.55,1.62)	7 (3,12)	0.09 (0.04,0.15)	0.6 (0.33,0.99)

Nigeria	6 (3,11)	0.01 (0,0.01)	0.94 (0.49,1.6)	159 (85,259)	0.17 (0.09,0.29)	0.61 (0.32,1.01)
North Africa and Middle East	582 (360,849)	0.13 (0.08,0.2)	10.67 (6.64,15.14)	15605 (9791,22589)	3.25 (2.03,4.76)	9.89 (6.25,13.69)
North Africa and Middle East	582 (360,849)	0.13 (0.08,0.2)	10.67 (6.64,15.14)	15605 (9791,22589)	3.25 (2.03,4.76)	9.89 (6.25,13.69)
Northern Mariana Islands	0 (0,0)	0.11 (0.06,0.18)	6.87 (3.97,10.61)	1 (1,2)	2.63 (1.41,4.24)	6.3 (3.68,9.57)
Norway	22 (12,33)	0.21 (0.12,0.32)	6.84 (4.02,10.29)	453 (266,677)	4.77 (2.81,7.04)	7.07 (4.28,10.38)
Oceania	1 (1,2)	0.02 (0.01,0.03)	4.92 (2.94,7.32)	40 (18,68)	0.49 (0.23,0.84)	4.24 (2.68,6.29)
Oman	1 (0,1)	0.05 (0.03,0.08)	5.69 (3.34,8.35)	24 (14,38)	1.12 (0.65,1.83)	5.41 (3.25,7.99)
Pakistan	78 (45,118)	0.07 (0.04,0.11)	7.39 (4.71,10.32)	1956 (1114,2947)	1.63 (0.93,2.46)	5.71 (3.61,8.11)
Palestine	4 (2,5)	0.15 (0.09,0.22)	11.57 (7.21,15.87)	96 (57,143)	3.56 (2.13,5.24)	10.69 (6.72,14.68)
Panama	4 (2,7)	0.09 (0.05,0.16)	4 (2.26,6.11)	93 (50,153)	2.12 (1.14,3.47)	3.27 (1.88,4.89)
Papua New Guinea	1 (0,1)	0.01 (0,0.03)	3.25 (1.59,5.47)	17 (4,36)	0.29 (0.08,0.62)	2.8 (1.37,4.84)
Paraguay	10 (5,17)	0.18 (0.09,0.31)	9.02 (5.34,13.5)	225 (116,375)	3.9 (2.02,6.52)	7.1 (4.18,10.65)

Peru	16 (8,29)	0.05 (0.03,0.09)	2.31 (1.25,3.77)	341 (179,588)	1.03 (0.54,1.78)	1.8 (0.99,2.83)
Philippines	78 (48,112)	0.1 (0.06,0.14)	8.68 (5.4,12.26)	2160 (1313,3083)	2.44 (1.49,3.49)	7.07 (4.46,9.91)
Plurinational State of Bolivia	6 (3,11)	0.07 (0.04,0.13)	2.86 (1.65,4.63)	145 (77,247)	1.57 (0.84,2.74)	2.36 (1.4,3.68)
Poland	406 (246,605)	0.55 (0.34,0.82)	11.3 (6.92,16.38)	9394 (5765,13703)	13.35 (8.26,19.32)	11.79 (7.4,16.65)
Portugal	34 (18,54)	0.14 (0.08,0.22)	7.36 (4.17,11)	792 (449,1193)	3.72 (2.17,5.49)	7.85 (4.61,11.26)
Principality of Monaco	0 (0,1)	0.48 (0.26,0.76)	10.47 (5.81,16.59)	10 (6,16)	11.6 (6.37,18.79)	10.41 (5.85,16.18)
Puerto Rico	9 (4,16)	0.12 (0.06,0.21)	6.38 (3.22,10.66)	194 (96,337)	2.98 (1.47,5.16)	5.34 (2.78,8.91)
Qatar	1 (1,2)	0.14 (0.07,0.26)	7.24 (4.15,11.21)	39 (19,70)	3.37 (1.59,6.07)	7.38 (4.27,11.26)
Republic of Cabo Verde	0 (0,0)	0.03 (0.01,0.06)	1.96 (1.09,3.13)	4 (2,6)	0.75 (0.39,1.35)	1.69 (0.95,2.79)
Republic of C 么 te d'Ivoire	1 (1,2)	0.01 (0.01,0.02)	3.15 (1.85,4.8)	31 (15,53)	0.26 (0.13,0.43)	2.45 (1.43,3.73)
Republic of Korea	193 (113,286)	0.2 (0.12,0.3)	12.59 (7.63,18.02)	4135 (2466,6042)	4.34 (2.6,6.32)	11.78 (7.29,16.62)
Republic of Moldova	20 (12,28)	0.33 (0.2,0.46)	13.86 (8.64,19.56)	572 (356,793)	9.74 (6.1,13.46)	14.5 (9.2,20.13)

Republic of Nauru	0 (0,0)	0.04 (0.02,0.07)	5.99 (3.33,8.94)	0 (0,0)	1.01 (0.53,1.69)	5.12 (2.88,7.69)
Republic of Niue	0 (0,0)	0.05 (0.03,0.08)	6.67 (3.64,10.39)	0 (0,0)	1.22 (0.64,2.08)	4.99 (2.67,7.93)
Republic of Palau	0 (0,0)	0.02 (0.01,0.03)	6.52 (3.66,10.6)	0 (0,0)	0.48 (0.27,0.81)	5.48 (3.1,8.79)
Republic of San Marino	0 (0,0)	0.14 (0.07,0.25)	10.94 (5.92,16.87)	2 (1,4)	3.45 (1.59,5.97)	10.85 (6.08,15.99)
Republic of the Gambia	0 (0,0)	0.01 (0,0.02)	2.29 (1.2,3.75)	2 (1,4)	0.24 (0.13,0.4)	1.83 (0.94,3.13)
Romania	114 (67,169)	0.32 (0.19,0.47)	10.12 (6.33,14.3)	2946 (1734,4326)	8.71 (5.14,12.7)	10.39 (6.55,14.33)
Russian Federation	1180 (753,1656)	0.48 (0.31,0.67)	11.79 (7.69,16.45)	31699 (20501,43854)	13.18 (8.51,18.14)	12.45 (8.27,17.23)
Rwanda	4 (2,6)	0.07 (0.03,0.12)	5.22 (3.05,7.7)	90 (42,152)	1.48 (0.71,2.48)	3.86 (2.3,5.78)
Saint Kitts and Nevis	0 (0,0)	0.1 (0.05,0.18)	4.09 (2.03,6.78)	2 (1,3)	2.35 (1.18,3.85)	3.39 (1.75,5.43)
Saint Lucia	0 (0,0)	0.09 (0.04,0.15)	5.02 (2.69,8.05)	5 (3,8)	2.04 (1.05,3.39)	4.04 (2.26,6.27)
Saint Vincent and the Grenadines	0 (0,0)	0.06 (0.03,0.09)	3.23 (1.68,5.12)	2 (1,3)	1.29 (0.69,2.06)	2.47 (1.34,3.82)
Samoa	0 (0,0)	0.03 (0.02,0.05)	8.49 (5.24,12.22)	1 (1,2)	0.8 (0.47,1.19)	7.69 (4.84,10.76)

Sao Tome and Principe	0 (0,0)	0.01 (0,0.02)	2.02 (1.1,3.55)	0 (0,0)	0.23 (0.12,0.41)	1.47 (0.68,2.95)
Saudi Arabia	22 (12,34)	0.11 (0.06,0.17)	7.22 (4.33,10.72)	701 (386,1127)	2.86 (1.64,4.54)	7.51 (4.56,11.03)
Senegal	1 (1,2)	0.02 (0.01,0.03)	2.52 (1.39,4.11)	36 (16,63)	0.43 (0.2,0.76)	2.06 (1.1,3.21)
Serbia	71 (42,104)	0.43 (0.25,0.63)	12.44 (7.65,17.57)	1744 (1040,2565)	11.14 (6.67,16.32)	13.08 (8.25,18.13)
Seychelles	0 (0,0)	0.13 (0.08,0.19)	10.14 (6.42,14.35)	4 (2,5)	3.03 (1.93,4.48)	8.86 (5.72,12.63)
Sierra Leone	0 (0,0)	0.01 (0,0.01)	1.46 (0.92,2.14)	7 (4,12)	0.19 (0.1,0.31)	1.02 (0.64,1.48)
Singapore	8 (5,12)	0.09 (0.05,0.14)	5.68 (3.27,8.41)	182 (109,273)	2.05 (1.22,3.08)	5.66 (3.34,8.27)
Slovakia	51 (30,77)	0.52 (0.3,0.78)	10.15 (6.03,14.7)	1250 (744,1886)	13.08 (7.78,19.73)	10.09 (6.19,14.22)
Slovenia	16 (8,26)	0.37 (0.2,0.6)	10.46 (5.71,15.69)	389 (208,616)	9.69 (5.29,15.24)	11.78 (6.83,17.19)
Socialist Republic of Viet Nam	57 (34,87)	0.06 (0.04,0.09)	10.65 (6.78,14.82)	1488 (900,2296)	1.42 (0.86,2.17)	9.99 (6.47,13.91)
Solomon Islands	0 (0,0)	0.04 (0.02,0.07)	9.02 (5.54,12.84)	4 (2,7)	1.09 (0.48,1.8)	8.08 (5.03,11.21)
Somalia	1 (0,2)	0.02 (0.01,0.03)	1.91 (0.9,3.33)	24 (10,47)	0.38 (0.16,0.72)	1.59 (0.75,2.85)

South Africa	26 (16,37)	0.06 (0.03,0.08)	4.03 (2.5,5.7)	692 (427,978)	1.43 (0.87,2.01)	3.57 (2.21,5.02)
South Asia	528 (329,739)	0.04 (0.02,0.05)	5.4 (3.37,7.54)	12512 (7705,17580)	0.84 (0.52,1.19)	4.34 (2.76,5.99)
South Asia	528 (329,739)	0.04 (0.02,0.05)	5.4 (3.37,7.54)	12512 (7705,17580)	0.84 (0.52,1.19)	4.34 (2.76,5.99)
South Sudan	1 (0,2)	0.02 (0.01,0.05)	1.72 (0.91,3.1)	22 (10,41)	0.56 (0.27,1.03)	1.28 (0.65,2.37)
Southeast Asia	487 (310,685)	0.08 (0.05,0.11)	7.98 (5.02,10.96)	12609 (7990,17738)	1.83 (1.16,2.57)	6.52 (4.11,8.98)
Southeast Asia, East Asia, and Oceania	4103 (2495,5730)	0.15 (0.09,0.2)	12.05 (7.58,16.19)	103184 (61903,144702)	3.47 (2.08,4.86)	10.51 (6.74,14.11)
Southern Latin America	345 (195,525)	0.4 (0.22,0.6)	8.69 (5.03,12.76)	8732 (5156,12825)	10.25 (6.06,14.98)	8.52 (5.08,12.32)
Southern Sub-Saharan Africa	34 (20,47)	0.06 (0.04,0.09)	4.17 (2.56,5.91)	890 (543,1247)	1.47 (0.9,2.06)	3.65 (2.3,5.12)
Spain	309 (168,463)	0.32 (0.18,0.47)	11.52 (6.67,16.83)	7075 (3961,10421)	8.09 (4.57,11.9)	11.83 (7.05,16.8)
Sri Lanka	8 (4,13)	0.03 (0.01,0.05)	3.51 (2.19,5.12)	168 (79,284)	0.61 (0.29,1.03)	2.85 (1.77,4.17)
Sub-Saharan Africa	92 (52,136)	0.02 (0.01,0.03)	2.08 (1.25,3.04)	2413 (1376,3541)	0.49 (0.28,0.73)	1.61 (0.99,2.31)
Sudan	15 (8,27)	0.08 (0.04,0.14)	7.2 (4.22,11.21)	416 (209,719)	1.99 (1,3.49)	6.11 (3.38,9.43)

Suriname	1 (0,1)	0.09 (0.05,0.15)	6.43 (3.72,9.98)	16 (9,25)	2.33 (1.26,3.83)	5.23 (3.02,7.97)
Sweden	75 (40,119)	0.32 (0.17,0.51)	10.98 (6.02,16.91)	1414 (773,2189)	6.82 (3.74,10.31)	11.22 (6.35,16.89)
Switzerland	44 (25,66)	0.23 (0.13,0.34)	11.64 (6.89,16.89)	892 (524,1315)	5.11 (3.04,7.47)	11.27 (6.81,16.22)
Syrian Arab Republic	17 (8,30)	0.14 (0.07,0.24)	10.7 (6.44,16.09)	438 (223,792)	3.12 (1.57,5.58)	9.61 (5.78,14.62)
Taiwan (Province of China)	105 (65,149)	0.24 (0.15,0.35)	8.83 (5.42,12.14)	2445 (1515,3439)	5.78 (3.61,8.09)	8.37 (5.25,11.42)
Tajikistan	6 (3,11)	0.11 (0.06,0.19)	7.56 (4.48,11.43)	191 (100,328)	2.86 (1.48,4.92)	6.83 (4.07,10.34)
Thailand	103 (60,167)	0.09 (0.05,0.15)	8.14 (5.12,11.33)	2455 (1427,4045)	2.19 (1.27,3.63)	6.54 (4.16,9.09)
The former Yugoslav Republic of Macedonia	9 (5,14)	0.27 (0.16,0.39)	14.04 (8.82,19.42)	239 (141,347)	6.82 (4.02,9.9)	14.93 (9.63,20.52)
Timor-Leste	0 (0,1)	0.04 (0.02,0.07)	6.38 (3.66,9.83)	9 (4,15)	1 (0.46,1.78)	4.96 (2.84,7.86)
Togo	1 (0,1)	0.02 (0.01,0.04)	4.15 (2.42,6.31)	24 (13,42)	0.59 (0.31,1.01)	3.22 (1.76,4.99)
Tokelau	0 (0,0)	0.04 (0.02,0.08)	5.95 (3.14,9.53)	0 (0,0)	0.98 (0.4,1.81)	3.8 (1.99,6.45)
Tonga	0 (0,0)	0.07 (0.04,0.12)	12.1 (7.24,17.49)	1 (1,2)	1.62 (0.78,2.68)	10.19 (6.1,15.09)

Trinidad and Tobago	2 (1,3)	0.09 (0.05,0.15)	4.67 (2.73,7.03)	45 (24,74)	2.26 (1.18,3.67)	3.78 (2.26,5.67)
Tropical Latin America	445 (248,686)	0.17 (0.1,0.27)	8.62 (4.7,13.49)	10553 (6022,15842)	4.03 (2.3,6.06)	7.44 (4.17,11.43)
Tunisia	24 (13,39)	0.19 (0.1,0.3)	14.28 (9.2,19.65)	581 (315,967)	4.24 (2.31,7)	12.96 (8.42,17.87)
Turkey	222 (129,335)	0.23 (0.14,0.35)	12 (7.42,16.9)	5924 (3462,8886)	6.02 (3.5,9.03)	11.58 (7.2,16.25)
Turkmenistan	12 (7,18)	0.27 (0.15,0.41)	8.17 (4.93,11.75)	362 (204,559)	7.75 (4.36,11.82)	7.81 (4.81,11.14)
Tuvalu	0 (0,0)	0.04 (0.02,0.07)	7.5 (4.41,11.54)	0 (0,0)	0.95 (0.49,1.59)	6.56 (3.86,10.03)
Uganda	2 (1,4)	0.02 (0.01,0.03)	1.05 (0.61,1.6)	62 (33,100)	0.41 (0.23,0.66)	0.82 (0.47,1.28)
Ukraine	303 (154,493)	0.39 (0.2,0.64)	10.8 (6.2,15.59)	8346 (4271,13602)	11.19 (5.74,18.23)	10.6 (6.07,15.07)
United Arab Emirates	4 (2,7)	0.15 (0.08,0.24)	5.82 (3.12,9.21)	138 (76,223)	3.02 (1.63,4.84)	5.36 (2.99,8.25)
United Kingdom of Great Britain and Northern Ireland	594 (318,938)	0.44 (0.24,0.68)	11.78 (6.36,18.38)	12078 (6803,18613)	9.76 (5.63,14.82)	11.27 (6.37,17.31)
United Republic of Tanzania	8 (3,14)	0.03 (0.01,0.06)	2.3 (1.25,3.74)	194 (83,362)	0.75 (0.32,1.39)	1.72 (0.92,2.83)
United States of America	2128 (1143,3337)	0.36 (0.2,0.56)	11.71 (6.34,18.16)	50007 (28671,76466)	8.82 (5.13,13.46)	11.74 (6.79,17.72)

United States Virgin Islands	0 (0,0)	0.09 (0.04,0.18)	4.71 (2.26,8.2)	4 (2,8)	2.25 (0.99,4.32)	3.47 (1.66,6.28)
Uruguay	38 (21,57)	0.71 (0.41,1.06)	10.98 (6.31,16.12)	910 (534,1331)	18.13 (10.82,26.34)	10.65 (6.35,15.31)
Uzbekistan	22 (12,34)	0.08 (0.05,0.13)	5.7 (3.35,8.24)	636 (350,977)	2.16 (1.2,3.3)	5.12 (3.04,7.48)
Vanuatu	0 (0,0)	0.02 (0.01,0.03)	3.92 (2.28,5.83)	1 (0,1)	0.4 (0.19,0.65)	3.28 (1.96,4.81)
Western Europe	3447 (1972,5219)	0.36 (0.21,0.53)	10.89 (6.26,16.29)	71438 (41622,104623)	8.25 (4.85,11.9)	10.88 (6.49,15.88)
Western Sub-Saharan Africa	17 (10,26)	0.01 (0.01,0.01)	1.31 (0.73,1.99)	444 (256,681)	0.22 (0.12,0.34)	0.91 (0.53,1.37)
Yemen	12 (6,20)	0.09 (0.04,0.15)	11.12 (6.91,15.85)	345 (163,554)	2.3 (1.07,3.72)	10 (6.16,14.18)
Zambia	2 (1,3)	0.03 (0.01,0.05)	1.35 (0.79,2)	49 (15,89)	0.71 (0.24,1.28)	1.05 (0.61,1.57)
Zimbabwe	5 (3,8)	0.08 (0.04,0.12)	5.37 (3.31,7.95)	132 (74,201)	1.84 (1.05,2.82)	4.51 (2.8,6.72)

ASMR, age-standardized mortality rates; ASDR, age-standardized disability-adjusted life years rates; DALYs, disability-adjusted life years; PAF, population attributable fraction; ASM, age-standardized mortality; ASD, age-standardized disability-adjusted life year; UI, uncertainty interval.

Supplementary Table 8. Average annual percentage change (AAPC) and estimated annual percentage change (EAPC) of age-standardized mortality rates (ASMR) and age-standardized disability-adjusted life year rates (ASDR) for prostate cancer attributable to smoking.

location_name	AAPC of ASMR (95% CI)	EAPC of ASMR (95% CI)	AAPC of ASDR (95% CI)	EAPC of ASDR (95% CI)
Afghanistan	0.48 (0.38,0.58)	0.8 (0.6,1)	0.56 (0.45,0.66)	0.85 (0.65,1.06)
Albania	-0.54 (-0.61,-0.46)	-0.64 (-0.78,-0.49)	-0.46 (-0.53,-0.39)	-0.46 (-0.6,-0.32)
Algeria	-0.70 (-0.77,-0.62)	-0.72 (-0.86,-0.58)	-0.64 (-0.71,-0.56)	-0.75 (-0.9,-0.61)
American Samoa	-0.12 (-0.21,-0.03)	0.24 (0.07,0.41)	-0.04 (-0.13,0.06)	0.33 (0.15,0.5)
Andean Latin America	-0.69 (-0.75,-0.63)	-0.91 (-1.02,-0.8)	-0.66 (-0.72,-0.59)	-0.9 (-1.02,-0.78)
Andorra	-1.68 (-1.79,-1.58)	-1.41 (-1.61,-1.2)	-1.50 (-1.61,-1.40)	-1.2 (-1.4,-1)
Angola	0.62 (0.50,0.74)	0.68 (0.46,0.91)	0.60 (0.47,0.72)	0.66 (0.42,0.9)
Antigua and Barbuda	-0.48 (-0.66,-0.30)	-0.64 (-0.98,-0.3)	-0.53 (-0.70,-0.36)	-0.9 (-1.22,-0.58)
Argentina	-1.29 (-1.50,-1.07)	-1.27 (-1.68,-0.86)	-1.34 (-1.56,-1.12)	-1.36 (-1.78,-0.95)

Armenia	1.35 (1.21,1.49)	2.01 (1.74,2.28)	1.20 (1.08,1.32)	1.78 (1.55,2.01)
Australasia	-4.01 (-4.21,-3.82)	-4.53 (-4.89,-4.16)	-3.81 (-4.06,-3.56)	-4.26 (-4.72,-3.81)
Australia	-4.32 (-4.57,-4.06)	-4.84 (-5.3,-4.37)	-4.04 (-4.35,-3.73)	-4.5 (-5.06,-3.94)
Austria	-1.63 (-1.78,-1.48)	-1.86 (-2.14,-1.58)	-1.29 (-1.45,-1.13)	-1.46 (-1.77,-1.16)
Azerbaijan	-0.26 (-0.43,-0.09)	0.46 (0.14,0.79)	-0.16 (-0.32,-0.00)	0.42 (0.12,0.73)
Bahamas	0.37 (0.28,0.47)	0.19 (0.01,0.37)	0.15 (0.07,0.22)	0.04 (-0.11,0.18)
Bahrain	-0.89 (-1.05,-0.74)	-1.43 (-1.73,-1.14)	-0.71 (-0.85,-0.58)	-1.26 (-1.52,-1)
Bangladesh	-0.39 (-0.51,-0.27)	-0.6 (-0.82,-0.37)	-0.55 (-0.68,-0.42)	-0.69 (-0.94,-0.44)
Barbados	-0.94 (-1.09,-0.80)	-1.24 (-1.51,-0.96)	-0.97 (-1.10,-0.83)	-1.27 (-1.53,-1.02)
Belarus	1.65 (1.51,1.78)	1.24 (0.97,1.51)	1.81 (1.65,1.96)	1.4 (1.1,1.7)

Belgium	-3.71 (-3.84,-3.58)	-4.24 (-4.48,-3.99)	-3.32 (-3.51,-3.13)	-3.78 (-4.12,-3.43)
Belize	0.85 (0.49,1.21)	0.31 (-0.38,1.01)	0.90 (0.56,1.25)	0.32 (-0.34,0.98)
Benin	0.24 (0.14,0.35)	0.3 (0.1,0.51)	0.18 (0.07,0.29)	0.22 (0.01,0.43)
Bermuda	-0.73 (-0.80,-0.66)	-0.73 (-0.86,-0.6)	-0.59 (-0.66,-0.52)	-0.62 (-0.75,-0.49)
Bhutan	-0.37 (-0.44,-0.30)	-0.41 (-0.54,-0.29)	-0.42 (-0.49,-0.35)	-0.48 (-0.61,-0.35)
Bolivia (Plurinational State of)	-0.81 (-0.86,-0.75)	-0.71 (-0.81,-0.61)	-0.85 (-0.90,-0.79)	-0.77 (-0.88,-0.67)
Bosnia and Herzegovina	0.78 (0.51,1.04)	1.17 (0.66,1.69)	0.80 (0.53,1.06)	1.27 (0.76,1.78)
Botswana	-0.71 (-0.84,-0.59)	-0.78 (-1.01,-0.54)	-0.65 (-0.77,-0.52)	-0.79 (-1.04,-0.55)
Brazil	-1.25 (-1.32,-1.18)	-1.43 (-1.56,-1.3)	-1.36 (-1.45,-1.28)	-1.66 (-1.82,-1.5)
Brunei Darussalam	-1.81 (-1.95,-1.66)	-1.27 (-1.54,-0.99)	-2.04 (-2.15,-1.93)	-1.67 (-1.87,-1.47)

Bulgaria	-0.36 (-0.53,-0.20)	-0.69 (-1,-0.37)	-0.21 (-0.38,-0.03)	-0.6 (-0.94,-0.27)
Burkina Faso	1.41 (1.28,1.53)	1.79 (1.55,2.03)	1.30 (1.17,1.43)	1.66 (1.41,1.92)
Burundi	-2.39 (-2.61,-2.16)	-2.55 (-2.96,-2.13)	-2.39 (-2.62,-2.16)	-2.56 (-2.99,-2.13)
Cabo Verde	1.10 (0.88,1.32)	0.37 (-0.06,0.79)	1.10 (0.88,1.32)	0.37 (-0.06,0.79)
Cambodia	1.20 (1.11,1.30)	1.31 (1.13,1.49)	1.04 (0.94,1.14)	1.1 (0.91,1.29)
Cameroon	0.75 (0.66,0.83)	0.97 (0.81,1.12)	0.73 (0.64,0.82)	0.93 (0.76,1.1)
Canada	-3.99 (-4.14,-3.84)	-4.76 (-5.03,-4.5)	-3.88 (-4.05,-3.71)	-4.74 (-5.05,-4.44)
Caribbean	-0.45 (-0.56,-0.35)	-0.64 (-0.83,-0.44)	-0.31 (-0.41,-0.21)	-0.49 (-0.68,-0.29)
Central African Republic	-0.63 (-0.74,-0.51)	-0.7 (-0.92,-0.47)	-0.61 (-0.73,-0.49)	-0.7 (-0.93,-0.47)
Central Asia	0.44 (0.27,0.61)	1.51 (1.18,1.84)	0.18 (0.03,0.33)	1.14 (0.85,1.43)

Central Europe	-0.91 (-1.05,-0.77)	-1.27 (-1.53,-1)	-0.87 (-1.00,-0.74)	-1.19 (-1.43,-0.94)
Central Europe, Eastern Europe, and Central Asia	0.18 (0.06,0.31)	0.17 (-0.07,0.42)	0.31 (0.18,0.44)	0.31 (0.07,0.56)
Central Latin America	-1.78 (-1.93,-1.62)	-2.26 (-2.55,-1.96)	-1.60 (-1.75,-1.46)	-2.12 (-2.4,-1.84)
Central Sub-Saharan Africa	0.34 (0.20,0.47)	0.4 (0.14,0.66)	0.28 (0.15,0.41)	0.34 (0.08,0.59)
Chad	1.41 (1.35,1.46)	1.52 (1.41,1.62)	1.42 (1.36,1.47)	1.52 (1.41,1.63)
Chile	-1.31 (-1.48,-1.13)	-1.53 (-1.85,-1.2)	-1.40 (-1.54,-1.26)	-1.64 (-1.9,-1.38)
China	-0.26 (-0.32,-0.20)	-0.38 (-0.5,-0.25)	-0.31 (-0.37,-0.26)	-0.41 (-0.51,-0.31)
Colombia	-1.71 (-1.89,-1.54)	-2.72 (-3.04,-2.39)	-1.61 (-1.79,-1.43)	-2.62 (-2.95,-2.28)
Comoros	-0.60 (-0.66,-0.54)	-0.88 (-0.98,-0.77)	-0.76 (-0.83,-0.69)	-1.08 (-1.21,-0.95)
Congo	0.36 (0.21,0.50)	0.4 (0.12,0.67)	0.30 (0.16,0.45)	0.33 (0.05,0.61)

Cook Islands	-0.61 (-0.65,-0.57)	-0.7 (-0.78,-0.63)	-0.51 (-0.55,-0.47)	-0.59 (-0.66,-0.51)
Costa Rica	-0.00 (-0.22,0.22)	-0.03 (-0.46,0.39)	0.29 (0.10,0.47)	0.22 (-0.13,0.58)
Croatia	-1.80 (-1.95,-1.66)	-1.33 (-1.6,-1.06)	-1.55 (-1.69,-1.40)	-1.06 (-1.34,-0.79)
Cuba	-0.21 (-0.28,-0.15)	-0.38 (-0.5,-0.27)	-0.04 (-0.11,0.03)	-0.19 (-0.32,-0.06)
Cyprus	-2.20 (-2.37,-2.04)	-1.79 (-2.1,-1.48)	-1.53 (-1.63,-1.43)	-1.19 (-1.38,-1)
Czechia	-1.30 (-1.45,-1.15)	-1.62 (-1.9,-1.34)	-1.10 (-1.25,-0.94)	-1.28 (-1.57,-0.99)
C 么 te d'Ivoire	-0.49 (-0.67,-0.30)	-1.17 (-1.51,-0.82)	-0.44 (-0.62,-0.26)	-1.12 (-1.45,-0.78)
Democratic People's Republic of Korea	-0.65 (-0.71,-0.59)	-0.72 (-0.83,-0.61)	-0.58 (-0.64,-0.52)	-0.64 (-0.75,-0.53)
Democratic Republic of the Congo	-0.32 (-0.46,-0.18)	-0.34 (-0.61,-0.08)	-0.35 (-0.49,-0.21)	-0.37 (-0.64,-0.1)
Denmark	-2.08 (-2.25,-1.91)	-2.16 (-2.47,-1.84)	-2.24 (-2.44,-2.04)	-2.21 (-2.58,-1.84)

Djibouti	-0.68 (-0.71,-0.65)	-0.76 (-0.81,-0.7)	-0.73 (-0.76,-0.70)	-0.83 (-0.89,-0.77)
Dominica	-0.28 (-0.39,-0.17)	-0.51 (-0.72,-0.3)	-0.33 (-0.45,-0.22)	-0.58 (-0.8,-0.37)
Dominican Republic	-0.49 (-0.75,-0.23)	-0.39 (-0.88,0.11)	-0.30 (-0.54,-0.06)	-0.24 (-0.7,0.23)
East Asia	-0.24 (-0.31,-0.17)	-0.37 (-0.49,-0.24)	-0.29 (-0.35,-0.24)	-0.41 (-0.51,-0.31)
Eastern Europe	1.11 (0.96,1.26)	1.26 (0.97,1.54)	1.25 (1.10,1.41)	1.37 (1.07,1.68)
Eastern Sub-Saharan Africa	-0.61 (-0.69,-0.53)	-0.86 (-1.01,-0.71)	-0.50 (-0.58,-0.42)	-0.76 (-0.91,-0.61)
Ecuador	-2.02 (-2.17,-1.87)	-2.09 (-2.37,-1.81)	-1.98 (-2.13,-1.82)	-2.13 (-2.42,-1.83)
Egypt	1.74 (1.55,1.92)	2.25 (1.9,2.62)	1.69 (1.53,1.86)	2.14 (1.81,2.46)
El Salvador	1.01 (0.88,1.14)	0.72 (0.47,0.98)	1.28 (1.15,1.41)	1.04 (0.79,1.29)
Equatorial Guinea	0.15 (0.11,0.19)	0.08 (0,0.16)	0.10 (0.05,0.15)	0.01 (-0.08,0.11)

Eritrea	-0.43 (-0.47,-0.39)	-0.59 (-0.66,-0.51)	-0.58 (-0.62,-0.53)	-0.76 (-0.84,-0.67)
Estonia	0.88 (0.41,1.35)	0.81 (-0.08,1.72)	0.88 (0.43,1.33)	0.92 (0.05,1.79)
Eswatini	-0.71 (-0.79,-0.63)	-0.34 (-0.49,-0.18)	-0.49 (-0.58,-0.41)	-0.28 (-0.43,-0.12)
Ethiopia	-0.65 (-0.83,-0.47)	-0.34 (-0.69,0)	-0.85 (-1.03,-0.67)	-0.6 (-0.94,-0.27)
Fiji	-0.28 (-0.38,-0.17)	-0.18 (-0.38,0.03)	-0.29 (-0.39,-0.19)	-0.19 (-0.38,0.01)
Finland	-1.93 (-2.08,-1.79)	-2.25 (-2.53,-1.98)	-1.59 (-1.78,-1.40)	-1.9 (-2.25,-1.55)
France	-3.87 (-3.99,-3.75)	-4.31 (-4.54,-4.09)	-3.31 (-3.41,-3.22)	-3.59 (-3.77,-3.42)
Gabon	0.45 (0.42,0.48)	0.42 (0.36,0.47)	0.47 (0.44,0.49)	0.44 (0.39,0.48)
Gambia	-0.62 (-0.69,-0.55)	-0.93 (-1.06,-0.8)	-0.63 (-0.71,-0.55)	-0.96 (-1.12,-0.81)
Georgia	2.97 (2.52,3.41)	5.14 (4.24,6.05)	2.80 (2.39,3.20)	4.82 (4.02,5.63)

Germany	-3.04 (-3.20,-2.87)	-3.7 (-4.01,-3.39)	-2.55 (-2.69,-2.42)	-3.13 (-3.38,-2.88)
Ghana	0.66 (0.47,0.85)	1.01 (0.65,1.37)	0.71 (0.52,0.89)	1.07 (0.72,1.42)
Greece	-2.75 (-2.93,-2.57)	-3.59 (-3.91,-3.26)	-2.54 (-2.70,-2.39)	-3.32 (-3.6,-3.03)
Greenland	-2.18 (-2.24,-2.11)	-2.21 (-2.33,-2.1)	-2.15 (-2.21,-2.09)	-2.16 (-2.27,-2.05)
Grenada	0.45 (-0.26,1.15)	0.21 (-1.13,1.57)	0.01 (-0.58,0.60)	-0.35 (-1.48,0.79)
Guam	-0.67 (-0.80,-0.55)	0.05 (-0.19,0.29)	-0.43 (-0.54,-0.32)	0.26 (0.04,0.47)
Guatemala	0.60 (0.19,1.01)	0.02 (-0.77,0.81)	0.82 (0.43,1.21)	0.18 (-0.57,0.94)
Guinea	0.63 (0.59,0.68)	0.68 (0.6,0.77)	0.67 (0.62,0.72)	0.72 (0.62,0.81)
Guinea-Bissau	1.91 (1.83,1.99)	2.36 (2.21,2.52)	1.92 (1.84,2.00)	2.39 (2.23,2.55)
Guyana	-0.17 (-0.27,-0.07)	-0.06 (-0.26,0.13)	-0.11 (-0.21,-0.01)	0 (-0.19,0.19)

Haiti	-0.30 (-0.35,-0.24)	-0.21 (-0.32,-0.1)	-0.45 (-0.52,-0.39)	-0.4 (-0.52,-0.27)
High SDI	-3.12 (-3.21,-3.04)	-3.63 (-3.78,-3.47)	-2.88 (-2.94,-2.81)	-3.3 (-3.43,-3.18)
High-income	-3.22 (-3.31,-3.14)	-3.74 (-3.9,-3.58)	-2.96 (-3.03,-2.89)	-3.4 (-3.53,-3.27)
High-income Asia Pacific	-1.67 (-1.84,-1.50)	-2.26 (-2.57,-1.94)	-1.60 (-1.76,-1.44)	-2.09 (-2.39,-1.79)
High-income North America	-3.35 (-3.48,-3.22)	-3.95 (-4.18,-3.71)	-3.14 (-3.27,-3.02)	-3.68 (-3.91,-3.46)
High-middle SDI	-1.57 (-1.66,-1.49)	-1.81 (-1.97,-1.64)	-1.38 (-1.46,-1.30)	-1.59 (-1.74,-1.44)
Honduras	0.46 (0.39,0.53)	0.51 (0.37,0.65)	0.57 (0.50,0.64)	0.63 (0.5,0.76)
Hungary	-2.45 (-2.60,-2.29)	-3.04 (-3.33,-2.75)	-2.16 (-2.31,-2.02)	-2.73 (-3,-2.46)
Iceland	-2.27 (-2.35,-2.18)	-2.5 (-2.66,-2.34)	-2.29 (-2.38,-2.20)	-2.64 (-2.8,-2.48)
India	-0.18 (-0.29,-0.08)	-0.61 (-0.81,-0.41)	-0.39 (-0.49,-0.29)	-0.83 (-1.02,-0.64)

Indonesia	1.54 (1.45,1.63)	1.51 (1.33,1.69)	1.44 (1.37,1.52)	1.41 (1.27,1.55)
Iran (Islamic Republic of)	0.31 (0.21,0.41)	0.87 (0.67,1.07)	0.41 (0.31,0.51)	0.95 (0.76,1.14)
Iraq	0.05 (-0.05,0.14)	-0.08 (-0.27,0.1)	0.01 (-0.08,0.11)	-0.08 (-0.26,0.1)
Ireland	-4.14 (-4.40,-3.87)	-4.87 (-5.36,-4.39)	-4.09 (-4.34,-3.84)	-4.68 (-5.14,-4.22)
Israel	-3.06 (-3.31,-2.81)	-3.82 (-4.27,-3.36)	-2.93 (-3.20,-2.66)	-3.59 (-4.09,-3.09)
Italy	-2.95 (-3.07,-2.82)	-3.43 (-3.66,-3.19)	-2.88 (-3.00,-2.77)	-3.3 (-3.52,-3.09)
Jamaica	0.96 (0.61,1.32)	0.62 (-0.06,1.3)	0.86 (0.48,1.23)	0.51 (-0.21,1.23)
Japan	-1.72 (-1.89,-1.55)	-2.34 (-2.66,-2.02)	-1.58 (-1.74,-1.42)	-2.11 (-2.42,-1.81)
Jordan	-1.36 (-1.39,-1.33)	-1.38 (-1.43,-1.32)	-1.14 (-1.17,-1.11)	-1.13 (-1.19,-1.07)
Kazakhstan	-0.97 (-1.15,-0.79)	-0.21 (-0.55,0.13)	-0.95 (-1.14,-0.75)	-0.26 (-0.63,0.1)

Kenya	0.19 (0.15,0.22)	0.14 (0.07,0.2)	0.15 (0.12,0.18)	0.06 (0,0.11)
Kiribati	0.91 (0.79,1.04)	0.82 (0.58,1.07)	0.85 (0.73,0.97)	0.76 (0.52,1)
Kuwait	1.65 (1.36,1.94)	1.27 (0.71,1.84)	1.73 (1.44,2.02)	1.32 (0.75,1.88)
Kyrgyzstan	0.62 (0.32,0.91)	1.77 (1.19,2.35)	0.45 (0.20,0.69)	1.51 (1.04,1.99)
Lao People's Democratic Republic	0.12 (0.10,0.15)	0.28 (0.23,0.33)	-0.05 (-0.08,-0.03)	0.08 (0.03,0.13)
Latin America and Caribbean	-1.28 (-1.37,-1.18)	-1.56 (-1.74,-1.39)	-1.28 (-1.38,-1.19)	-1.63 (-1.81,-1.45)
Latvia	0.56 (0.36,0.75)	1.03 (0.65,1.41)	0.58 (0.38,0.78)	0.99 (0.6,1.37)
Lebanon	0.90 (0.72,1.08)	1.2 (0.86,1.55)	0.95 (0.78,1.12)	1.24 (0.92,1.57)
Lesotho	1.18 (1.09,1.26)	1.69 (1.53,1.85)	1.46 (1.39,1.54)	1.84 (1.69,1.99)
Liberia	0.68 (0.63,0.72)	0.68 (0.6,0.77)	0.77 (0.73,0.82)	0.79 (0.7,0.88)

Libya	-0.23 (-0.27,-0.18)	-0.05 (-0.14,0.04)	-0.06 (-0.10,-0.02)	0.08 (0,0.16)
Lithuania	0.87 (0.57,1.17)	1.06 (0.48,1.64)	1.07 (0.77,1.37)	1.28 (0.7,1.87)
Low SDI	-0.32 (-0.38,-0.26)	-0.52 (-0.64,-0.41)	-0.30 (-0.36,-0.24)	-0.53 (-0.64,-0.42)
Low-middle SDI	0.04 (0.01,0.08)	-0.04 (-0.12,0.03)	0.02 (-0.01,0.05)	-0.09 (-0.15,-0.03)
Luxembourg	-2.79 (-2.85,-2.74)	-3.01 (-3.12,-2.91)	-2.64 (-2.69,-2.58)	-2.87 (-2.97,-2.76)
Madagascar	-2.84 (-3.09,-2.60)	-3.12 (-3.57,-2.66)	-2.88 (-3.13,-2.64)	-3.17 (-3.62,-2.72)
Malawi	0.98 (0.90,1.06)	1.07 (0.92,1.23)	1.09 (1.00,1.17)	1.15 (0.99,1.32)
Malaysia	-1.11 (-1.27,-0.96)	-1.51 (-1.8,-1.22)	-1.12 (-1.24,-1.00)	-1.48 (-1.71,-1.26)
Maldives	-1.63 (-1.70,-1.55)	-1.97 (-2.11,-1.83)	-1.80 (-1.88,-1.71)	-2.21 (-2.37,-2.05)
Mali	1.91 (1.79,2.04)	2.47 (2.23,2.72)	1.90 (1.77,2.03)	2.45 (2.2,2.71)

Malta	-2.98 (-3.12,-2.85)	-3.58 (-3.84,-3.33)	-2.78 (-2.91,-2.65)	-3.32 (-3.55,-3.08)
Marshall Islands	0.46 (0.44,0.48)	0.53 (0.48,0.57)	0.45 (0.42,0.48)	0.54 (0.48,0.59)
Mauritania	0.60 (0.53,0.67)	0.65 (0.52,0.79)	0.60 (0.53,0.67)	0.64 (0.51,0.78)
Mauritius	0.88 (0.69,1.08)	0.01 (-0.37,0.39)	1.04 (0.83,1.25)	0.04 (-0.36,0.45)
Mexico	-2.81 (-2.99,-2.64)	-3.2 (-3.52,-2.87)	-2.64 (-2.80,-2.48)	-3.07 (-3.37,-2.77)
Micronesia (Federated States of)	0.26 (0.25,0.28)	0.23 (0.2,0.26)	0.28 (0.27,0.29)	0.25 (0.23,0.28)
Middle SDI	-0.86 (-0.93,-0.80)	-1.05 (-1.17,-0.94)	-0.81 (-0.86,-0.75)	-1.02 (-1.11,-0.92)
Monaco	-1.00 (-1.02,-0.97)	-1.09 (-1.14,-1.04)	-0.86 (-0.88,-0.84)	-0.93 (-0.98,-0.89)
Mongolia	2.06 (1.97,2.16)	2.66 (2.47,2.85)	2.06 (1.95,2.16)	2.7 (2.5,2.9)
Montenegro	0.39 (0.27,0.51)	-0.02 (-0.24,0.21)	0.34 (0.25,0.43)	0.03 (-0.16,0.21)

Morocco	-0.51 (-0.64,-0.38)	-0.68 (-0.93,-0.43)	-0.22 (-0.36,-0.07)	-0.28 (-0.57,0)
Mozambique	0.35 (0.28,0.42)	0.66 (0.52,0.8)	0.45 (0.38,0.52)	0.75 (0.62,0.88)
Myanmar	-1.71 (-1.77,-1.64)	-1.79 (-1.91,-1.67)	-1.80 (-1.87,-1.74)	-1.91 (-2.04,-1.78)
Namibia	0.39 (0.32,0.46)	0.79 (0.66,0.93)	0.44 (0.37,0.50)	0.78 (0.65,0.9)
Nauru	0.40 (0.37,0.42)	0.39 (0.34,0.45)	0.41 (0.38,0.44)	0.41 (0.35,0.47)
Nepal	-0.83 (-0.94,-0.71)	-0.94 (-1.16,-0.72)	-0.90 (-1.02,-0.79)	-1.04 (-1.26,-0.82)
Netherlands	-2.49 (-2.58,-2.40)	-2.87 (-3.03,-2.7)	-2.21 (-2.31,-2.11)	-2.43 (-2.61,-2.24)
New Zealand	-2.69 (-2.82,-2.56)	-3.07 (-3.31,-2.82)	-2.75 (-2.88,-2.63)	-3.11 (-3.33,-2.88)
Nicaragua	-0.51 (-0.60,-0.41)	-0.3 (-0.48,-0.12)	-0.18 (-0.27,-0.09)	0.06 (-0.11,0.22)
Niger	1.52 (1.46,1.58)	1.87 (1.76,1.99)	1.46 (1.40,1.51)	1.81 (1.7,1.92)

Nigeria	0.80 (0.76,0.84)	0.95 (0.88,1.03)	0.94 (0.89,0.99)	1.1 (1,1.19)
Niue	0.42 (0.40,0.44)	0.44 (0.4,0.47)	0.38 (0.36,0.40)	0.38 (0.35,0.42)
North Africa and Middle East	-0.38 (-0.43,-0.33)	-0.95 (-1.02,-0.89)	-0.30 (-0.34,-0.26)	-0.77 (-0.83,-0.72)
North Macedonia	-0.15 (-0.35,0.05)	-0.22 (-0.6,0.17)	-0.13 (-0.29,0.02)	-0.18 (-0.49,0.12)
Northern Mariana Islands	0.59 (0.50,0.67)	0.81 (0.64,0.97)	0.63 (0.55,0.71)	0.87 (0.71,1.02)
Norway	-3.31 (-3.50,-3.12)	-3.95 (-4.31,-3.6)	-3.39 (-3.60,-3.18)	-3.96 (-4.35,-3.57)
Oceania	-0.19 (-0.23,-0.14)	-0.1 (-0.19,-0.02)	-0.05 (-0.09,-0.02)	0.03 (-0.05,0.1)
Oman	-0.76 (-0.88,-0.65)	-0.41 (-0.63,-0.2)	-0.64 (-0.75,-0.54)	-0.32 (-0.52,-0.11)
Pakistan	-0.64 (-0.79,-0.49)	-0.89 (-1.17,-0.6)	-0.62 (-0.78,-0.47)	-0.89 (-1.18,-0.6)
Palau	-0.83 (-0.87,-0.79)	-0.86 (-0.93,-0.79)	-0.70 (-0.74,-0.67)	-0.74 (-0.81,-0.68)

Palestine	-0.56 (-0.71,-0.40)	-0.6 (-0.9,-0.31)	-0.42 (-0.55,-0.30)	-0.51 (-0.75,-0.27)
Panama	-0.96 (-1.19,-0.72)	-1.22 (-1.67,-0.78)	-0.76 (-0.97,-0.54)	-1.07 (-1.47,-0.66)
Papua New Guinea	0.50 (0.46,0.54)	0.58 (0.51,0.65)	0.54 (0.50,0.58)	0.61 (0.53,0.69)
Paraguay	0.55 (0.43,0.66)	0.47 (0.24,0.69)	0.58 (0.47,0.70)	0.41 (0.19,0.63)
Peru	0.06 (-0.06,0.18)	-0.33 (-0.56,-0.1)	0.13 (0.01,0.26)	-0.28 (-0.52,-0.04)
Philippines	-0.78 (-0.83,-0.74)	-0.49 (-0.58,-0.41)	-0.62 (-0.66,-0.58)	-0.47 (-0.54,-0.39)
Poland	-1.12 (-1.33,-0.90)	-1.84 (-2.24,-1.44)	-1.32 (-1.51,-1.14)	-1.99 (-2.33,-1.64)
Portugal	-3.25 (-3.43,-3.06)	-4.17 (-4.51,-3.83)	-2.77 (-2.93,-2.60)	-3.61 (-3.92,-3.3)
Puerto Rico	-1.85 (-1.95,-1.74)	-2.3 (-2.5,-2.1)	-1.45 (-1.55,-1.34)	-1.86 (-2.05,-1.66)
Qatar	-0.89 (-1.12,-0.67)	-1.08 (-1.5,-0.66)	-0.66 (-0.85,-0.47)	-0.61 (-0.96,-0.25)

Republic of Korea	-1.05 (-1.24,-0.86)	-1.49 (-1.85,-1.14)	-1.06 (-1.23,-0.89)	-1.39 (-1.71,-1.07)
Republic of Moldova	1.64 (1.33,1.94)	2.83 (2.23,3.43)	1.86 (1.58,2.14)	3.09 (2.54,3.65)
Romania	0.08 (-0.06,0.21)	-0.37 (-0.62,-0.11)	0.24 (0.12,0.37)	-0.18 (-0.42,0.06)
Russian Federation	2.26 (2.03,2.49)	2.47 (2.03,2.92)	2.24 (2.01,2.46)	2.39 (1.94,2.83)
Rwanda	0.07 (0.01,0.13)	-0.24 (-0.36,-0.13)	-0.07 (-0.14,-0.00)	-0.44 (-0.57,-0.31)
Saint Kitts and Nevis	-0.25 (-0.34,-0.17)	-0.57 (-0.74,-0.41)	-0.22 (-0.31,-0.13)	-0.39 (-0.56,-0.21)
Saint Lucia	-1.44 (-1.65,-1.23)	-2.28 (-2.67,-1.89)	-1.42 (-1.62,-1.22)	-2.17 (-2.54,-1.8)
Saint Vincent and the Grenadines	0.84 (0.69,0.99)	0.53 (0.25,0.82)	0.73 (0.59,0.87)	0.37 (0.1,0.64)
Samoa	-0.34 (-0.36,-0.33)	-0.4 (-0.43,-0.38)	-0.31 (-0.32,-0.29)	-0.38 (-0.41,-0.35)
San Marino	-3.08 (-3.30,-2.86)	-2.05 (-2.46,-1.64)	-2.78 (-2.97,-2.60)	-1.93 (-2.27,-1.58)

Sao Tome and Principe	0.11 (0.05,0.18)	0.1 (-0.03,0.23)	0.33 (0.25,0.42)	0.19 (0.04,0.35)
Saudi Arabia	-0.39 (-0.54,-0.24)	-0.67 (-0.97,-0.38)	0.17 (0.03,0.31)	-0.04 (-0.31,0.22)
Senegal	1.37 (1.27,1.46)	1.68 (1.5,1.87)	1.26 (1.17,1.35)	1.55 (1.37,1.74)
Serbia	-1.20 (-1.47,-0.94)	-1.06 (-1.56,-0.56)	-0.88 (-1.15,-0.61)	-0.79 (-1.31,-0.27)
Seychelles	0.21 (0.01,0.40)	0.12 (-0.25,0.49)	0.14 (-0.05,0.33)	0.04 (-0.32,0.4)
Sierra Leone	0.41 (0.36,0.46)	0.67 (0.57,0.77)	0.50 (0.44,0.56)	0.79 (0.68,0.91)
Singapore	-1.85 (-1.95,-1.74)	-2.2 (-2.4,-2)	-1.84 (-1.93,-1.74)	-2.09 (-2.26,-1.91)
Slovakia	-0.50 (-0.55,-0.45)	-0.35 (-0.45,-0.26)	-0.45 (-0.50,-0.41)	-0.32 (-0.4,-0.23)
Slovenia	-0.52 (-0.75,-0.28)	-0.54 (-0.98,-0.09)	-0.56 (-0.81,-0.31)	-0.48 (-0.95,0)
Solomon Islands	0.72 (0.62,0.83)	0.99 (0.79,1.19)	0.79 (0.68,0.91)	1.07 (0.85,1.29)

Somalia	-0.77 (-0.81,-0.72)	-0.77 (-0.86,-0.67)	-0.76 (-0.81,-0.71)	-0.82 (-0.91,-0.72)
South Africa	-2.43 (-2.47,-2.39)	-2.52 (-2.59,-2.45)	-2.04 (-2.08,-2.01)	-2.09 (-2.15,-2.02)
South Asia	-0.21 (-0.29,-0.12)	-0.8 (-0.92,-0.69)	-0.27 (-0.36,-0.19)	-0.94 (-1.05,-0.83)
South Sudan	-0.48 (-0.58,-0.39)	-0.66 (-0.84,-0.48)	-0.50 (-0.61,-0.40)	-0.7 (-0.9,-0.5)
Southeast Asia	-0.01 (-0.05,0.04)	-0.03 (-0.12,0.06)	0.06 (0.02,0.10)	0.03 (-0.04,0.1)
Southeast Asia, East Asia, and Oceania	-0.19 (-0.24,-0.14)	-0.26 (-0.35,-0.17)	-0.20 (-0.23,-0.17)	-0.28 (-0.34,-0.22)
Southern Latin America	-1.33 (-1.53,-1.13)	-1.39 (-1.77,-1.01)	-1.42 (-1.62,-1.22)	-1.51 (-1.9,-1.13)
Southern Sub-Saharan Africa	-1.72 (-1.78,-1.67)	-1.71 (-1.82,-1.61)	-1.36 (-1.41,-1.31)	-1.34 (-1.44,-1.25)
Spain	-3.87 (-3.99,-3.76)	-4.49 (-4.71,-4.28)	-3.62 (-3.73,-3.52)	-4.19 (-4.38,-4)
Sri Lanka	-2.13 (-2.27,-1.98)	-2.19 (-2.46,-1.92)	-2.04 (-2.16,-1.91)	-2.14 (-2.38,-1.91)

Sub-Saharan Africa	-0.53 (-0.56,-0.50)	-0.58 (-0.63,-0.52)	-0.31 (-0.33,-0.28)	-0.35 (-0.39,-0.3)
Sudan	0.30 (0.27,0.33)	0.36 (0.3,0.42)	0.29 (0.25,0.32)	0.32 (0.26,0.38)
Suriname	-0.72 (-0.89,-0.54)	-0.81 (-1.14,-0.48)	-0.69 (-0.88,-0.51)	-0.86 (-1.21,-0.5)
Sweden	-1.89 (-2.05,-1.72)	-2.13 (-2.43,-1.82)	-2.06 (-2.25,-1.88)	-2.14 (-2.48,-1.79)
Switzerland	-4.06 (-4.20,-3.92)	-4.21 (-4.47,-3.96)	-3.91 (-4.00,-3.83)	-4.1 (-4.26,-3.94)
Syrian Arab Republic	-0.92 (-0.99,-0.84)	-1.13 (-1.27,-0.98)	-0.79 (-0.88,-0.70)	-1.06 (-1.23,-0.89)
Taiwan (Province of China)	0.28 (0.11,0.45)	-0.05 (-0.37,0.27)	0.33 (0.16,0.50)	-0.06 (-0.39,0.27)
Tajikistan	-3.55 (-3.68,-3.43)	-3.66 (-3.89,-3.44)	-3.28 (-3.39,-3.17)	-3.33 (-3.53,-3.12)
Thailand	-1.39 (-1.46,-1.32)	-1.63 (-1.77,-1.5)	-1.11 (-1.18,-1.05)	-1.28 (-1.4,-1.16)
Timor-Leste	0.23 (0.19,0.27)	0.23 (0.15,0.31)	0.23 (0.18,0.28)	0.22 (0.13,0.31)

Togo	0.87 (0.82,0.92)	1.14 (1.03,1.24)	0.95 (0.90,1.00)	1.21 (1.1,1.31)
Tokelau	-0.19 (-0.22,-0.17)	-0.23 (-0.28,-0.18)	-0.15 (-0.18,-0.13)	-0.2 (-0.25,-0.15)
Tonga	-0.09 (-0.14,-0.04)	-0.14 (-0.24,-0.04)	-0.05 (-0.10,-0.00)	-0.09 (-0.18,0.01)
Trinidad and Tobago	-1.17 (-1.31,-1.03)	-1.78 (-2.05,-1.52)	-1.06 (-1.20,-0.92)	-1.7 (-1.96,-1.44)
Tropical Latin America	-1.21 (-1.28,-1.14)	-1.39 (-1.52,-1.26)	-1.32 (-1.41,-1.24)	-1.62 (-1.78,-1.46)
Tunisia	-0.45 (-0.51,-0.40)	-0.65 (-0.75,-0.56)	-0.29 (-0.32,-0.25)	-0.45 (-0.51,-0.39)
Turkmenistan	-0.97 (-1.15,-0.80)	-0.43 (-0.77,-0.09)	-0.87 (-1.04,-0.70)	-0.26 (-0.59,0.06)
Tuvalu	0.31 (0.28,0.34)	0.33 (0.28,0.38)	0.30 (0.27,0.33)	0.32 (0.26,0.37)
T 眉 rkiye	-2.36 (-2.54,-2.18)	-3.16 (-3.5,-2.82)	-2.10 (-2.25,-1.94)	-2.82 (-3.1,-2.53)
Uganda	0.17 (-0.01,0.34)	-0.6 (-0.93,-0.27)	0.23 (0.05,0.42)	-0.57 (-0.91,-0.23)

Ukraine	-1.00 (-1.12,-0.88)	-1.13 (-1.36,-0.91)	-0.80 (-0.91,-0.68)	-0.87 (-1.08,-0.65)
United Arab Emirates	-0.38 (-0.65,-0.11)	1.24 (0.72,1.76)	-0.58 (-0.83,-0.34)	0.85 (0.38,1.32)
United Kingdom	-2.95 (-3.05,-2.84)	-3.48 (-3.67,-3.29)	-2.91 (-3.00,-2.83)	-3.39 (-3.55,-3.24)
United Republic of Tanzania	-1.10 (-1.20,-1.00)	-1.54 (-1.73,-1.36)	-1.07 (-1.17,-0.97)	-1.52 (-1.71,-1.33)
United States of America	-3.28 (-3.41,-3.15)	-3.86 (-4.09,-3.62)	-3.07 (-3.19,-2.95)	-3.57 (-3.8,-3.35)
United States Virgin Islands	-1.42 (-1.60,-1.23)	-1.71 (-2.05,-1.36)	-1.61 (-1.81,-1.41)	-1.96 (-2.34,-1.58)
Uruguay	-0.42 (-0.60,-0.23)	-0.65 (-1.01,-0.3)	-0.60 (-0.81,-0.39)	-0.85 (-1.24,-0.45)
Uzbekistan	0.40 (0.05,0.74)	0.52 (-0.14,1.18)	0.36 (0.02,0.70)	0.49 (-0.17,1.15)
Vanuatu	0.12 (0.06,0.18)	-0.07 (-0.18,0.04)	0.07 (0.02,0.13)	-0.15 (-0.25,-0.04)
Venezuela (Bolivarian Republic of)	-0.65 (-0.85,-0.44)	-1.31 (-1.7,-0.92)	-0.61 (-0.81,-0.41)	-1.33 (-1.7,-0.95)

Viet Nam	0.10 (-0.03,0.23)	-0.17 (-0.42,0.08)	0.20 (0.09,0.32)	-0.03 (-0.25,0.19)
Western Europe	-3.18 (-3.28,-3.08)	-3.69 (-3.87,-3.51)	-2.90 (-2.99,-2.81)	-3.32 (-3.49,-3.16)
Western Sub-Saharan Africa	0.77 (0.74,0.80)	0.85 (0.8,0.9)	0.84 (0.81,0.88)	0.91 (0.84,0.98)
Yemen	0.25 (0.23,0.28)	0.19 (0.15,0.24)	0.25 (0.22,0.27)	0.19 (0.14,0.23)
Zambia	1.11 (0.89,1.32)	1.3 (0.89,1.72)	1.32 (1.09,1.55)	1.53 (1.08,1.98)
Zimbabwe	0.71 (0.55,0.86)	1.01 (0.71,1.31)	0.90 (0.73,1.06)	1.12 (0.8,1.44)

AAPC, average annual percentage change; EAPC, estimated annual percentage change; ASMR, age-standardized mortality rates; ASDR, age-standardized disability-adjusted life year rates; CI, confidence interval.

Supplementary Table 9. The global burden of prostate cancer attributable to smoking in 2021.

location_name	Deaths cases (95% UI)	ASMR (95% UI)	PAF of ASM (95% UI)	DALYs cases (95% UI)	ASDR (95% UI)	PAF of ADM (95% UI)
Afghanistan	6 (2,12)	0.2 (0.07,0.36)	2.3 (1,4.12)	130 (48,250)	3.7 (1.37,6.89)	2.54 (1.13,4.49)
Albania	23 (9,41)	1.14 (0.47,2.05)	7.26 (3.28,11.3)	413 (176,732)	19.49 (8.3,34.41)	7.66 (3.44,11.66)

Algeria	18 (7,34)	0.13 (0.05,0.24)	4.44 (1.95,7.58)	367 (142,673)	2.28 (0.87,4.14)	4.92 (2.24,8.16)
American Samoa	0 (0,0)	1.03 (0.45,1.87)	1.95 (0.87,3.27)	5 (2,9)	20.85 (9.1,37.42)	2.42 (1.09,4)
Andean Latin America	95 (40,183)	0.37 (0.15,0.71)	1.65 (0.74,2.89)	1823 (739,3502)	6.79 (2.76,13.04)	1.81 (0.83,3.07)
Andorra	1 (0,1)	0.69 (0.27,1.36)	3.26 (1.41,5.91)	11 (4,21)	14.21 (5.53,27.58)	3.86 (1.69,6.75)
Angola	19 (7,35)	0.53 (0.2,0.97)	1.82 (0.82,3.02)	472 (173,862)	10.43 (3.83,19.06)	2.14 (0.99,3.53)
Antigua and Barbuda	1 (0,1)	1.34 (0.54,2.61)	1.75 (0.72,3.28)	12 (5,23)	25.37 (10.39,48.28)	2.01 (0.84,3.61)
Argentina	105 (47,181)	0.44 (0.19,0.76)	2 (0.9,3.43)	2343 (1060,3957)	9.51 (4.3,16.07)	2.54 (1.19,4.19)
Armenia	19 (9,30)	1.04 (0.49,1.7)	6.67 (3.08,10.47)	424 (201,674)	22.39 (10.63,35.94)	7.42 (3.49,11.55)
Australasia	80 (33,149)	0.3 (0.12,0.56)	1.52 (0.64,2.73)	1745 (744,3054)	6.82 (2.92,11.9)	1.92 (0.85,3.24)
Australia	61 (24,116)	0.27 (0.11,0.52)	1.39 (0.6,2.49)	1359 (572,2395)	6.33 (2.67,11.08)	1.8 (0.81,3.01)
Austria	50 (22,87)	0.59 (0.26,1.03)	3.44 (1.57,5.8)	1096 (494,1877)	13.49 (6.08,22.96)	4.42 (2.06,7.14)
Azerbaijan	22 (9,37)	0.59 (0.25,1.01)	6.5 (2.95,10.25)	564 (242,937)	12.77 (5.53,21.63)	7 (3.22,11.02)

Bahamas	2 (1,4)	1.43 (0.56,2.69)	2.09 (0.87,3.84)	47 (19,86)	27.81 (11.17,50.76)	2.35 (1,4.25)
Bahrain	2 (1,3)	0.64 (0.27,1.2)	3.28 (1.43,5.65)	44 (16,86)	12.09 (4.99,22.43)	3.65 (1.62,6.12)
Bangladesh	125 (48,252)	0.19 (0.07,0.39)	3.51 (1.6,5.65)	2544 (962,5268)	3.68 (1.41,7.54)	3.9 (1.82,6.22)
Barbados	2 (1,4)	0.91 (0.34,1.78)	1.74 (0.72,3.21)	38 (15,71)	15.91 (6.14,29.74)	1.83 (0.79,3.28)
Belarus	72 (31,120)	1.29 (0.55,2.1)	6.11 (2.92,9.77)	1810 (769,3050)	29.57 (12.69,49.56)	6.83 (3.23,10.85)
Belgium	75 (33,131)	0.66 (0.29,1.15)	3.69 (1.67,6.36)	1447 (658,2458)	13.31 (6.09,22.51)	4.33 (2,7.21)
Belize	1 (0,1)	0.66 (0.28,1.16)	1.93 (0.86,3.2)	19 (8,32)	13.18 (5.65,22.7)	2.19 (0.99,3.58)
Benin	3 (1,6)	0.17 (0.06,0.33)	0.59 (0.26,1.03)	77 (26,142)	3.52 (1.21,6.57)	0.72 (0.3,1.21)
Bermuda	1 (0,1)	0.93 (0.37,1.69)	2.09 (0.85,3.68)	12 (5,22)	19.22 (7.77,34.48)	2.45 (1.04,4.18)
Bhutan	0 (0,1)	0.12 (0.04,0.28)	2.13 (0.88,4)	6 (2,14)	2.05 (0.67,4.87)	2.23 (0.94,4.19)
Bolivia (Plurinational State of)	17 (6,33)	0.46 (0.16,0.93)	1.51 (0.65,2.7)	352 (127,688)	8.76 (3.11,17.38)	1.72 (0.76,2.98)
Bosnia and Herzegovina	21 (8,38)	0.76 (0.3,1.38)	4.7 (2.16,7.5)	431 (171,787)	15.17 (6.03,27.89)	5.23 (2.41,8.15)

Botswana	5 (2,9)	1.01 (0.41,1.8)	2.32 (1.1,3.78)	117 (48,212)	20.39 (8.54,37.32)	2.66 (1.26,4.29)
Brazil	668 (291,1183)	0.65 (0.28,1.17)	3.02 (1.33,5.39)	13850 (6101,23775)	12.68 (5.59,21.95)	3.38 (1.5,5.86)
Brunei Darussalam	0 (0,1)	0.4 (0.16,0.74)	2.66 (1.14,4.86)	10 (4,18)	7.07 (2.91,12.97)	3.1 (1.33,5.41)
Bulgaria	43 (19,71)	0.68 (0.3,1.13)	2.94 (1.32,4.64)	976 (431,1604)	15.55 (6.84,25.5)	3.79 (1.73,5.86)
Burkina Faso	6 (2,12)	0.17 (0.06,0.33)	0.62 (0.27,1.12)	146 (49,274)	3.52 (1.17,6.69)	0.76 (0.34,1.31)
Burundi	3 (1,7)	0.16 (0.05,0.32)	0.7 (0.32,1.17)	82 (28,166)	3.39 (1.14,6.74)	0.83 (0.38,1.38)
Cabo Verde	1 (0,1)	0.32 (0.11,0.67)	0.59 (0.25,1.03)	12 (4,26)	6.74 (2.45,14.23)	0.75 (0.32,1.27)
Cambodia	30 (11,54)	0.85 (0.31,1.52)	5.57 (2.49,9.06)	655 (232,1158)	15.22 (5.53,27.16)	5.95 (2.68,9.58)
Cameroon	12 (4,23)	0.24 (0.07,0.47)	0.73 (0.33,1.28)	314 (97,597)	5.31 (1.66,10.09)	0.94 (0.43,1.59)
Canada	162 (67,299)	0.46 (0.19,0.86)	2.89 (1.24,5.11)	3186 (1392,5555)	9.2 (4.03,16.03)	3.46 (1.54,5.84)
Caribbean	227 (97,402)	0.93 (0.39,1.65)	2.41 (1.09,4)	4711 (2087,8324)	18.84 (8.34,33.35)	2.77 (1.27,4.48)
Central African Republic	2 (1,3)	0.27 (0.1,0.53)	1.05 (0.44,1.86)	45 (16,87)	5.48 (1.97,10.57)	1.21 (0.53,2.08)

Central Asia	125 (59,193)	0.43 (0.2,0.67)	4.7 (2.27,7.3)	3070 (1436,4702)	9.06 (4.27,14)	5.2 (2.52,8.01)
Central Europe	644 (297,1041)	0.67 (0.31,1.09)	3.13 (1.47,5)	13865 (6446,22402)	14.15 (6.59,22.85)	3.71 (1.75,5.8)
Central Europe, Eastern Europe, and Central Asia	1878 (893,3038)	0.74 (0.35,1.2)	3.91 (1.87,6.29)	44957 (21377,71229)	16.62 (7.91,26.67)	4.68 (2.25,7.4)
Central Latin America	291 (129,497)	0.27 (0.12,0.47)	1.41 (0.65,2.32)	6351 (2878,10677)	5.7 (2.55,9.63)	1.59 (0.74,2.58)
Central Sub-Saharan Africa	44 (17,76)	0.25 (0.1,0.46)	0.92 (0.42,1.53)	1087 (413,1901)	5.07 (1.98,8.8)	1.1 (0.51,1.79)
Chad	6 (2,11)	0.22 (0.07,0.43)	0.86 (0.37,1.56)	124 (41,244)	4.35 (1.47,8.47)	0.98 (0.42,1.77)
Chile	32 (13,60)	0.29 (0.12,0.54)	1.22 (0.51,2.18)	693 (311,1233)	5.99 (2.69,10.68)	1.53 (0.69,2.56)
China	1935 (826,3286)	0.23 (0.1,0.38)	4.6 (2.11,7.14)	40781 (17562,68287)	4.19 (1.79,7.03)	5.42 (2.5,8.24)
Colombia	51 (22,90)	0.21 (0.09,0.38)	1.2 (0.52,2.05)	1133 (491,1963)	4.62 (2,8.05)	1.37 (0.6,2.24)
Comoros	1 (0,1)	0.36 (0.13,0.7)	1.35 (0.62,2.32)	14 (5,28)	6.91 (2.52,13.66)	1.45 (0.66,2.5)
Congo	4 (2,8)	0.5 (0.18,0.95)	1.58 (0.72,2.68)	104 (35,194)	9.71 (3.33,18.21)	1.84 (0.85,3.06)
Cook Islands	0 (0,0)	1.21 (0.5,2.19)	1.94 (0.83,3.31)	3 (1,5)	23.91 (9.78,42.92)	2.32 (1,3.86)

Costa Rica	12 (5,22)	0.5 (0.21,0.89)	2.28 (1.01,3.93)	280 (121,481)	11.19 (4.83,19.28)	2.69 (1.23,4.48)
Croatia	38 (16,64)	0.98 (0.42,1.64)	3.99 (1.83,6.44)	738 (321,1226)	18.69 (8.13,30.94)	4.42 (2.02,7.25)
Cuba	111 (46,184)	1.2 (0.5,2)	3.04 (1.37,4.99)	2354 (1001,3963)	25.59 (10.8,43.08)	3.63 (1.67,5.82)
Cyprus	9 (4,17)	1.01 (0.4,1.86)	4.42 (1.92,7.51)	200 (83,362)	20.09 (8.36,36.08)	5.27 (2.44,8.69)
Czechia	67 (29,113)	0.68 (0.3,1.15)	3.46 (1.55,5.59)	1500 (661,2450)	15.25 (6.68,24.78)	4.32 (1.96,6.79)
C 么 te d'Ivoire	21 (8,38)	0.5 (0.2,0.92)	1.12 (0.47,1.95)	502 (188,898)	9.81 (3.84,17.86)	1.27 (0.55,2.18)
Democratic People's Republic of Korea	19 (8,33)	0.17 (0.07,0.29)	3.09 (1.36,4.89)	463 (186,827)	3.52 (1.42,6.2)	3.77 (1.67,5.91)
Democratic Republic of the Congo	17 (7,31)	0.14 (0.05,0.27)	0.53 (0.24,0.91)	424 (167,774)	2.87 (1.1,5.37)	0.65 (0.29,1.09)
Denmark	79 (35,132)	1.35 (0.59,2.26)	4.54 (2.04,7.63)	1397 (625,2277)	23.87 (10.67,38.84)	5.01 (2.3,8.18)
Djibouti	1 (0,3)	0.55 (0.17,1.09)	1.89 (0.83,3.15)	33 (10,68)	10.88 (3.34,22.25)	2.06 (0.87,3.44)
Dominica	0 (0,1)	0.94 (0.35,1.94)	1.45 (0.61,2.62)	7 (3,13)	18.06 (6.88,36.18)	1.68 (0.72,2.99)
Dominican Republic	39 (14,88)	0.87 (0.31,1.98)	2.75 (1.18,4.68)	739 (266,1659)	16.17 (5.86,36.6)	3.03 (1.34,5.16)

East Asia	2019 (861,3407)	0.23 (0.1,0.38)	4.5 (2.06,6.93)	42557 (18315,70623)	4.23 (1.81,7.06)	5.29 (2.45,8.03)
Eastern Europe	1109 (525,1787)	0.85 (0.4,1.37)	4.62 (2.19,7.53)	28022 (13268,44380)	20.19 (9.56,32.26)	5.44 (2.6,8.69)
Eastern Sub-Saharan Africa	150 (59,252)	0.23 (0.09,0.39)	1.15 (0.54,1.8)	3629 (1436,6208)	4.88 (1.93,8.25)	1.31 (0.62,2.05)
Ecuador	22 (9,40)	0.31 (0.13,0.58)	1.32 (0.56,2.3)	423 (177,753)	5.7 (2.37,10.13)	1.53 (0.68,2.6)
Egypt	83 (33,144)	0.33 (0.14,0.57)	4.23 (1.97,6.67)	1954 (731,3358)	6.67 (2.67,11.49)	4.74 (2.23,7.34)
El Salvador	9 (4,18)	0.33 (0.14,0.68)	1.53 (0.61,2.81)	190 (80,379)	7.39 (3.09,14.65)	1.85 (0.78,3.25)
Equatorial Guinea	1 (0,1)	0.32 (0.1,0.61)	1.01 (0.41,1.75)	12 (4,23)	6.43 (2.09,12.38)	1.2 (0.51,2.09)
Eritrea	2 (1,4)	0.2 (0.06,0.41)	0.74 (0.31,1.29)	48 (14,96)	4.4 (1.28,8.96)	0.88 (0.38,1.55)
Estonia	11 (5,19)	1.1 (0.47,1.87)	3.22 (1.42,5.46)	267 (117,450)	26.13 (11.47,43.97)	4.01 (1.86,6.59)
Eswatini	1 (0,1)	0.4 (0.14,0.75)	0.93 (0.41,1.54)	15 (5,29)	8.04 (2.89,15.15)	0.99 (0.44,1.64)
Ethiopia	8 (3,17)	0.05 (0.02,0.09)	0.51 (0.25,0.87)	189 (65,376)	0.93 (0.32,1.84)	0.6 (0.29,1)
Fiji	1 (0,2)	0.44 (0.1,0.88)	1.78 (0.79,2.97)	27 (6,57)	8.43 (1.91,17.2)	2.2 (0.99,3.55)

Finland	27 (11,50)	0.44 (0.18,0.8)	2.33 (0.97,4.14)	595 (251,1053)	10.12 (4.27,17.86)	2.86 (1.24,4.97)
France	345 (145,635)	0.51 (0.22,0.92)	2.79 (1.2,5)	7032 (3108,11764)	11.14 (4.98,18.18)	3.44 (1.56,5.55)
Gabon	1 (0,2)	0.34 (0.11,0.67)	0.94 (0.4,1.64)	30 (9,59)	6.68 (2.07,12.98)	1.13 (0.49,1.91)
Gambia	0 (0,1)	0.08 (0.03,0.16)	1.14 (0.53,1.99)	7 (3,14)	1.71 (0.72,3.3)	1.38 (0.63,2.37)
Georgia	40 (17,62)	1.69 (0.74,2.67)	6.28 (2.85,9.88)	890 (392,1407)	36.72 (16.18,57.91)	7.02 (3.31,11.03)
Germany	534 (232,929)	0.57 (0.25,0.97)	2.98 (1.3,5.15)	11106 (4980,18588)	12.48 (5.6,20.81)	3.57 (1.62,5.85)
Ghana	21 (9,39)	0.44 (0.18,0.81)	1.19 (0.49,2.09)	456 (186,849)	7.71 (3.12,14.15)	1.2 (0.51,2.11)
Global	12992 (5893,21582)	0.36 (0.16,0.6)	2.82 (1.32,4.68)	281979 (131119,458053)	7.17 (3.32,11.81)	3.29 (1.56,5.33)
Greece	108 (48,186)	0.82 (0.38,1.38)	4.62 (2.13,7.6)	1852 (853,3103)	16.1 (7.48,26.48)	5.33 (2.51,8.6)
Greenland	0 (0,0)	0.39 (0.17,0.71)	3.13 (1.37,5.31)	3 (1,6)	8.36 (3.55,14.77)	3.89 (1.74,6.38)
Grenada	0 (0,1)	1.11 (0.45,2.09)	1.18 (0.5,2.12)	10 (4,17)	20.84 (8.87,37.86)	1.35 (0.61,2.31)
Guam	0 (0,0)	0.25 (0.11,0.47)	2 (0.86,3.48)	6 (3,11)	5.81 (2.51,10.72)	2.42 (1.06,4.15)

Guatemala	15 (6,26)	0.32 (0.13,0.57)	1.4 (0.56,2.42)	295 (123,510)	6.08 (2.5,10.53)	1.59 (0.68,2.65)
Guinea	5 (2,10)	0.22 (0.08,0.39)	1.13 (0.5,1.93)	119 (47,217)	4.4 (1.69,7.95)	1.33 (0.59,2.22)
Guinea-Bissau	1 (0,1)	0.24 (0.08,0.48)	0.73 (0.31,1.3)	16 (5,32)	5.11 (1.61,10.45)	0.89 (0.37,1.56)
Guyana	2 (1,4)	0.83 (0.33,1.57)	1.58 (0.68,2.85)	49 (20,92)	17.12 (6.99,32.66)	1.82 (0.8,3.16)
Haiti	12 (4,23)	0.47 (0.17,0.92)	0.87 (0.35,1.59)	263 (101,490)	8.83 (3.32,16.82)	0.98 (0.41,1.75)
High SDI	4627 (2061,7895)	0.46 (0.21,0.79)	3.01 (1.34,5.1)	99057 (45349,162756)	10.14 (4.66,16.59)	3.61 (1.66,5.94)
High-income	4904 (2183,8453)	0.46 (0.21,0.79)	2.96 (1.32,5.08)	103397 (47627,170201)	10.21 (4.71,16.73)	3.56 (1.61,5.85)
High-income Asia Pacific	536 (239,919)	0.23 (0.1,0.39)	2.96 (1.33,4.96)	9667 (4462,15619)	4.38 (2.03,7.03)	3.4 (1.56,5.48)
High-income North America	1556 (652,2810)	0.5 (0.21,0.92)	3.07 (1.29,5.52)	38256 (17307,64627)	12.37 (5.58,20.82)	3.78 (1.69,6.45)
High-middle SDI	3515 (1591,5643)	0.42 (0.19,0.69)	3.58 (1.69,5.73)	78210 (35829,122601)	8.72 (3.97,13.82)	4.33 (2.09,6.76)
Honduras	11 (4,28)	0.46 (0.16,1.08)	2.32 (1.03,3.92)	251 (87,594)	9.09 (3.13,21.7)	2.72 (1.21,4.59)
Hungary	44 (19,75)	0.55 (0.24,0.92)	2.94 (1.37,4.85)	999 (433,1675)	12.27 (5.34,20.45)	3.57 (1.68,5.8)

Iceland	2 (1,4)	0.79 (0.34,1.43)	3.45 (1.45,6.35)	46 (19,80)	15.81 (6.67,27.58)	4.05 (1.7,6.87)
India	636 (272,1131)	0.14 (0.06,0.25)	2.48 (1.12,3.96)	12860 (5545,22766)	2.48 (1.06,4.43)	2.57 (1.15,4.05)
Indonesia	358 (148,621)	0.43 (0.18,0.76)	3.25 (1.54,5.09)	8173 (3345,14251)	8.03 (3.33,13.99)	3.6 (1.71,5.58)
Iran (Islamic Republic of)	94 (40,162)	0.28 (0.12,0.48)	2.58 (1.19,4.41)	2104 (861,3624)	5.75 (2.37,9.92)	3.05 (1.43,4.96)
Iraq	30 (13,54)	0.38 (0.16,0.7)	5.2 (2.39,8.34)	656 (270,1206)	6.99 (2.95,12.83)	5.49 (2.5,8.76)
Ireland	18 (8,33)	0.48 (0.2,0.86)	2.8 (1.24,4.9)	360 (153,615)	9.34 (3.97,15.89)	3.2 (1.43,5.52)
Israel	16 (7,29)	0.27 (0.11,0.49)	2.53 (1.12,4.34)	320 (145,548)	5.56 (2.53,9.5)	3.09 (1.44,5.17)
Italy	306 (129,525)	0.42 (0.18,0.72)	2.98 (1.3,5.06)	5948 (2611,9690)	8.94 (3.99,14.35)	3.63 (1.62,5.86)
Jamaica	18 (7,36)	1.29 (0.52,2.5)	2.28 (0.99,3.97)	376 (151,719)	25.93 (10.45,49.63)	2.58 (1.13,4.27)
Japan	436 (199,772)	0.23 (0.11,0.39)	2.93 (1.33,4.99)	7696 (3602,12811)	4.48 (2.12,7.19)	3.41 (1.59,5.46)
Jordan	9 (4,17)	0.33 (0.13,0.6)	4.36 (2.06,6.92)	232 (94,416)	6.74 (2.72,12.02)	5 (2.33,7.91)
Kazakhstan	19 (8,29)	0.28 (0.13,0.44)	2.73 (1.27,4.4)	508 (233,802)	6.62 (3.02,10.47)	3.41 (1.62,5.43)

Kenya	16 (6,28)	0.18 (0.07,0.32)	1.1 (0.5,1.77)	384 (152,669)	3.85 (1.55,6.75)	1.26 (0.58,2.02)
Kiribati	0 (0,0)	0.59 (0.25,1.05)	4.24 (1.98,7.02)	3 (1,5)	11.73 (5.08,20.09)	5.02 (2.36,8.08)
Kuwait	4 (2,7)	0.36 (0.15,0.62)	3.66 (1.58,6.17)	99 (42,171)	7.2 (3.04,12.59)	4.14 (1.86,6.9)
Kyrgyzstan	8 (4,14)	0.52 (0.23,0.84)	6.86 (3.05,11.04)	209 (94,347)	10.78 (4.82,17.57)	7.39 (3.39,11.69)
Lao People's Democratic Republic	7 (3,12)	0.43 (0.17,0.75)	3.81 (1.77,5.97)	148 (60,254)	7.86 (3.13,13.59)	4.13 (1.91,6.47)
Latin America and Caribbean	1300 (576,2266)	0.5 (0.22,0.87)	2.22 (0.99,3.77)	27125 (12388,45957)	9.86 (4.47,16.86)	2.48 (1.13,4.13)
Latvia	14 (6,24)	0.99 (0.4,1.69)	2.96 (1.35,4.9)	326 (134,534)	22.46 (9.25,36.78)	3.61 (1.68,5.81)
Lebanon	28 (11,52)	0.93 (0.38,1.72)	5.05 (2.19,8.39)	511 (201,917)	17.91 (7.02,31.79)	5.71 (2.59,9.18)
Lesotho	5 (2,9)	1.42 (0.54,2.76)	3.19 (1.5,5.03)	113 (42,214)	29.58 (11.1,55.62)	3.5 (1.62,5.52)
Liberia	1 (0,3)	0.16 (0.05,0.31)	0.62 (0.27,1.09)	35 (11,66)	3.48 (1.12,6.47)	0.79 (0.35,1.33)
Libya	8 (3,16)	0.41 (0.15,0.74)	2.92 (1.28,4.69)	196 (75,350)	8.32 (3.17,15.14)	3.43 (1.51,5.48)
Lithuania	22 (9,37)	1.02 (0.43,1.71)	3.48 (1.56,5.8)	504 (216,834)	23.6 (10.17,39.07)	3.96 (1.82,6.57)

Low SDI	371 (157,612)	0.19 (0.08,0.31)	1.12 (0.52,1.84)	8520 (3523,13974)	3.76 (1.57,6.2)	1.28 (0.61,2.09)
Low-middle SDI	1469 (650,2368)	0.26 (0.12,0.42)	2.37 (1.11,3.78)	31499 (14101,50256)	4.96 (2.21,8.01)	2.62 (1.24,4.13)
Luxembourg	3 (1,5)	0.54 (0.23,0.95)	3.22 (1.38,5.72)	55 (23,94)	11.01 (4.69,19)	3.92 (1.72,6.76)
Madagascar	5 (2,9)	0.12 (0.04,0.23)	0.66 (0.31,1.1)	119 (39,233)	2.46 (0.81,4.71)	0.73 (0.34,1.19)
Malawi	13 (5,24)	0.53 (0.21,0.97)	2.45 (1.03,4.14)	291 (114,543)	10.28 (4.07,19.23)	2.57 (1.07,4.24)
Malaysia	31 (12,53)	0.25 (0.1,0.42)	2.42 (1.12,3.9)	653 (267,1143)	4.89 (1.96,8.5)	2.66 (1.24,4.2)
Maldives	0 (0,1)	0.23 (0.08,0.44)	3.56 (1.7,5.71)	6 (2,10)	3.98 (1.55,7.21)	3.77 (1.79,5.97)
Mali	6 (2,10)	0.16 (0.07,0.29)	1.34 (0.59,2.25)	122 (47,214)	3.07 (1.21,5.34)	1.46 (0.64,2.4)
Malta	1 (1,3)	0.31 (0.12,0.55)	2.83 (1.22,5)	32 (13,55)	6.84 (2.86,11.75)	3.5 (1.6,5.83)
Marshall Islands	0 (0,0)	0.56 (0.21,1.06)	1.87 (0.81,3.29)	2 (1,3)	10.94 (4.02,19.4)	2.21 (0.96,3.86)
Mauritania	2 (1,3)	0.19 (0.06,0.36)	0.63 (0.28,1.03)	43 (14,80)	4.08 (1.36,7.59)	0.79 (0.36,1.28)
Mauritius	4 (2,7)	0.59 (0.27,0.95)	3.48 (1.57,5.51)	97 (44,154)	11.69 (5.43,18.56)	3.85 (1.75,6.07)

Mexico	118 (54,192)	0.22 (0.1,0.37)	1.29 (0.58,2.09)	2534 (1170,4077)	4.49 (2.08,7.26)	1.45 (0.67,2.31)
Micronesia (Federated States of)	0 (0,0)	0.68 (0.27,1.16)	2.15 (0.92,3.62)	4 (2,8)	13.85 (5.55,23.22)	2.66 (1.13,4.4)
Middle SDI	2989 (1349,4870)	0.28 (0.12,0.45)	2.74 (1.28,4.4)	64234 (29527,101335)	5.29 (2.41,8.5)	3.13 (1.48,4.96)
Monaco	0 (0,1)	0.82 (0.32,1.62)	3.37 (1.35,6.01)	8 (3,16)	16.85 (6.64,33.09)	4.02 (1.61,6.99)
Mongolia	2 (1,3)	0.19 (0.08,0.33)	4.36 (2.02,7.03)	43 (17,72)	4.43 (1.88,7.57)	5.15 (2.4,8.14)
Montenegro	6 (2,10)	1.42 (0.61,2.5)	4.63 (2.09,7.81)	130 (56,227)	29.02 (12.38,50.36)	5.52 (2.54,9.14)
Morocco	16 (5,30)	0.1 (0.03,0.19)	1.65 (0.73,2.77)	372 (123,707)	2.2 (0.73,4.13)	2.07 (0.93,3.41)
Mozambique	4 (2,8)	0.13 (0.05,0.25)	1.13 (0.49,1.94)	100 (41,187)	2.47 (0.99,4.67)	1.24 (0.54,2.1)
Myanmar	42 (17,77)	0.26 (0.1,0.49)	2.38 (1.04,4.12)	858 (348,1608)	4.71 (1.89,8.75)	2.49 (1.09,4.17)
Namibia	2 (1,4)	0.59 (0.22,0.98)	1.55 (0.73,2.5)	53 (19,90)	11.07 (4.04,18.36)	1.55 (0.73,2.46)
Nauru	0 (0,0)	0.66 (0.25,1.34)	2 (0.86,3.46)	0 (0,1)	13.09 (4.95,27.35)	2.39 (1.08,4.11)
Nepal	12 (5,23)	0.13 (0.05,0.25)	2.47 (1.12,3.94)	241 (99,483)	2.35 (0.96,4.66)	2.66 (1.21,4.24)

Netherlands	123 (54,220)	0.71 (0.31,1.28)	3.43 (1.52,5.98)	2401 (1072,4078)	13.79 (6.18,23.38)	3.83 (1.73,6.43)
New Zealand	19 (8,34)	0.45 (0.19,0.84)	2.18 (0.9,3.93)	386 (162,695)	9.45 (3.97,16.97)	2.55 (1.1,4.39)
Nicaragua	6 (3,11)	0.33 (0.14,0.61)	2.2 (0.95,3.89)	140 (58,255)	6.83 (2.84,12.44)	2.46 (1.1,4.37)
Niger	4 (1,8)	0.15 (0.05,0.31)	0.62 (0.26,1.09)	84 (25,173)	2.57 (0.81,5.14)	0.64 (0.28,1.13)
Nigeria	109 (31,218)	0.32 (0.09,0.64)	0.67 (0.29,1.2)	2397 (686,4755)	6.46 (1.83,12.95)	0.8 (0.34,1.43)
Niue	0 (0,0)	0.64 (0.23,1.3)	1.98 (0.85,3.57)	0 (0,0)	12.63 (4.66,25.17)	2.42 (1.04,4.25)
North Africa and Middle East	601 (257,1036)	0.33 (0.14,0.57)	3.31 (1.57,5.37)	13522 (5673,23089)	6.59 (2.8,11.31)	3.79 (1.8,6.04)
North Africa and Middle East	601 (257,1036)	0.33 (0.14,0.57)	3.31 (1.57,5.37)	13522 (5673,23089)	6.59 (2.8,11.31)	3.79 (1.8,6.04)
North Macedonia	10 (4,17)	0.68 (0.26,1.21)	3.94 (1.8,6.29)	228 (90,396)	14.05 (5.62,24.5)	4.68 (2.15,7.34)
Northern Mariana Islands	0 (0,0)	0.53 (0.22,0.95)	1.9 (0.81,3.3)	3 (1,5)	10.54 (4.46,18.56)	2.32 (1.02,3.97)
Norway	22 (9,38)	0.45 (0.19,0.77)	1.8 (0.77,3.06)	418 (183,694)	8.75 (3.82,14.44)	2.2 (0.98,3.65)
Oceania	10 (4,18)	0.33 (0.13,0.61)	1.52 (0.67,2.5)	232 (90,428)	6.63 (2.58,12.2)	1.84 (0.8,3.02)

Oman	0 (0,1)	0.07 (0.03,0.13)	1.51 (0.66,2.49)	12 (5,21)	1.4 (0.56,2.46)	1.74 (0.76,2.79)
Pakistan	137 (59,242)	0.27 (0.11,0.47)	2.37 (1.09,3.74)	3077 (1334,5464)	5.34 (2.3,9.38)	2.72 (1.24,4.26)
Palau	0 (0,0)	0.6 (0.24,1.16)	2.07 (0.89,3.68)	1 (1,3)	12.18 (4.92,22.94)	2.45 (1.06,4.26)
Palestine	7 (3,12)	0.82 (0.34,1.56)	4.18 (1.88,6.96)	156 (64,280)	15.36 (6.35,28.77)	4.81 (2.18,7.73)
Panama	7 (3,13)	0.33 (0.14,0.61)	1.59 (0.7,2.7)	140 (60,257)	6.69 (2.86,12.22)	1.75 (0.78,2.95)
Papua New Guinea	5 (2,11)	0.24 (0.08,0.51)	1.14 (0.47,1.98)	123 (42,263)	4.94 (1.71,10.58)	1.42 (0.59,2.41)
Paraguay	20 (7,40)	0.83 (0.28,1.67)	3.29 (1.4,5.89)	389 (134,786)	15.33 (5.28,30.98)	3.53 (1.52,6.23)
Peru	57 (23,115)	0.37 (0.15,0.77)	1.85 (0.8,3.16)	1049 (413,2121)	6.82 (2.69,13.77)	1.97 (0.85,3.33)
Philippines	149 (65,252)	0.49 (0.21,0.82)	2.98 (1.39,4.71)	3571 (1564,6026)	10.11 (4.37,17.01)	3.44 (1.63,5.45)
Poland	230 (107,395)	0.77 (0.36,1.34)	2.96 (1.35,4.97)	4828 (2263,8020)	15.52 (7.19,25.63)	3.49 (1.64,5.64)
Portugal	40 (17,72)	0.35 (0.15,0.62)	1.82 (0.82,3.08)	845 (374,1427)	8.02 (3.58,13.35)	2.41 (1.08,3.84)
Puerto Rico	16 (7,31)	0.46 (0.19,0.85)	2.42 (1,4.45)	326 (140,589)	9.97 (4.27,18.18)	2.79 (1.19,4.85)

Qatar	1 (0,2)	0.41 (0.16,0.82)	2.01 (0.89,3.52)	36 (13,73)	8.58 (3.27,17.29)	2.41 (1.06,4.19)
Republic of Korea	96 (37,165)	0.27 (0.1,0.48)	3.52 (1.53,5.98)	1870 (748,3122)	4.74 (1.87,8.05)	3.82 (1.71,6.25)
Republic of Moldova	21 (9,36)	0.87 (0.37,1.47)	6.12 (2.72,10.05)	553 (243,928)	21.77 (9.56,36.74)	6.93 (3.2,11.21)
Romania	63 (27,104)	0.39 (0.17,0.64)	2.26 (1.06,3.72)	1487 (629,2412)	9.25 (3.92,14.8)	2.82 (1.3,4.5)
Russian Federation	775 (369,1246)	0.88 (0.42,1.43)	4.72 (2.24,7.68)	19872 (9470,30860)	21 (10.07,33.27)	5.61 (2.67,8.98)
Rwanda	13 (4,24)	0.67 (0.21,1.27)	2.41 (0.99,4.19)	293 (100,547)	12.8 (4.08,23.7)	2.56 (1.07,4.34)
Saint Kitts and Nevis	0 (0,0)	1.2 (0.48,2.31)	1.27 (0.51,2.46)	6 (2,10)	21.1 (8.63,40.36)	1.39 (0.58,2.54)
Saint Lucia	1 (0,2)	1.1 (0.44,2.1)	1.45 (0.59,2.7)	22 (9,41)	20.39 (8.45,37.93)	1.69 (0.73,2.99)
Saint Vincent and the Grenadines	1 (0,1)	1.19 (0.5,2.28)	1.45 (0.6,2.74)	16 (7,29)	22.72 (9.97,40.92)	1.71 (0.78,3.06)
Samoa	0 (0,1)	0.49 (0.17,0.88)	3.27 (1.54,5.34)	7 (2,12)	9.84 (3.28,17.99)	3.92 (1.85,6.31)
San Marino	0 (0,0)	0.41 (0.16,0.86)	3.49 (1.47,6.33)	3 (1,7)	8.63 (3.51,17.42)	4.04 (1.75,7.09)
Sao Tome and Principe	0 (0,0)	0.16 (0.06,0.33)	0.66 (0.27,1.24)	2 (1,3)	3.22 (1.25,6.62)	0.83 (0.35,1.53)

Saudi Arabia	6 (2,14)	0.08 (0.03,0.18)	1.63 (0.71,2.8)	188 (72,438)	1.82 (0.7,4.32)	2.09 (0.92,3.58)
Senegal	11 (3,21)	0.33 (0.1,0.63)	1 (0.43,1.73)	269 (85,498)	7.32 (2.27,13.62)	1.31 (0.57,2.23)
Serbia	46 (20,83)	0.6 (0.26,1.08)	3.15 (1.44,5.02)	980 (408,1754)	12.62 (5.31,22.69)	3.71 (1.72,5.86)
Seychelles	1 (0,1)	1.78 (0.75,3.03)	3.5 (1.59,5.72)	16 (7,28)	32.88 (13.91,56.05)	3.79 (1.7,6.14)
Sierra Leone	3 (1,6)	0.19 (0.07,0.35)	0.72 (0.34,1.23)	74 (26,137)	4.02 (1.42,7.55)	0.91 (0.43,1.45)
Singapore	4 (2,7)	0.1 (0.04,0.17)	1.35 (0.61,2.25)	91 (39,150)	2.17 (0.93,3.58)	1.68 (0.76,2.79)
Slovakia	29 (12,48)	0.75 (0.3,1.29)	3.45 (1.58,5.81)	633 (261,1063)	15.33 (6.4,25.91)	3.89 (1.81,6.31)
Slovenia	15 (6,29)	0.77 (0.31,1.45)	2.87 (1.19,5.26)	320 (132,585)	15.87 (6.53,28.96)	3.61 (1.56,6.31)
Solomon Islands	1 (0,2)	0.69 (0.28,1.24)	2.8 (1.26,4.54)	23 (9,40)	14.4 (5.72,26.06)	3.52 (1.62,5.65)
Somalia	5 (1,10)	0.24 (0.07,0.53)	1.18 (0.48,2.06)	118 (34,251)	5.15 (1.45,11.31)	1.33 (0.55,2.31)
South Africa	81 (33,131)	0.5 (0.21,0.8)	1.18 (0.54,1.89)	1994 (818,3249)	10.55 (4.35,17)	1.44 (0.65,2.28)
South Asia	911 (410,1578)	0.15 (0.07,0.27)	2.56 (1.19,4.06)	18728 (8293,32376)	2.84 (1.26,4.93)	2.72 (1.27,4.24)

South Asia	911 (410,1578)	0.15 (0.07,0.27)	2.56 (1.19,4.06)	18728 (8293,32376)	2.84 (1.26,4.93)	2.72 (1.27,4.24)
South Sudan	4 (1,8)	0.25 (0.08,0.51)	1.13 (0.51,2.01)	98 (32,198)	5.31 (1.75,10.73)	1.29 (0.58,2.27)
Southeast Asia	828 (350,1384)	0.34 (0.14,0.57)	3.09 (1.47,4.78)	18635 (7952,30672)	6.61 (2.82,11.03)	3.46 (1.65,5.27)
Southeast Asia, East Asia, and Oceania	2856 (1232,4672)	0.25 (0.11,0.4)	4 (1.88,6.11)	61424 (26286,100583)	4.73 (2.03,7.77)	4.59 (2.17,6.99)
Southern Latin America	158 (70,273)	0.42 (0.19,0.73)	1.81 (0.79,3.06)	3472 (1605,5868)	8.99 (4.13,15.19)	2.28 (1.06,3.74)
Southern Sub-Saharan Africa	128 (55,206)	0.64 (0.27,1.02)	1.44 (0.65,2.3)	3131 (1362,4997)	13.47 (5.79,21.44)	1.74 (0.79,2.77)
Spain	230 (99,413)	0.48 (0.21,0.86)	3.33 (1.47,5.62)	4341 (1892,7423)	9.79 (4.33,16.67)	3.94 (1.76,6.43)
Sri Lanka	9 (4,16)	0.09 (0.04,0.16)	1.39 (0.61,2.24)	187 (77,336)	1.62 (0.68,2.92)	1.49 (0.66,2.32)
Sub-Saharan Africa	542 (214,897)	0.31 (0.12,0.53)	0.97 (0.45,1.59)	12826 (5109,20995)	6.31 (2.49,10.46)	1.16 (0.55,1.86)
Sudan	20 (8,39)	0.24 (0.09,0.47)	2.95 (1.22,5.07)	431 (162,820)	4.6 (1.73,8.86)	3.25 (1.34,5.41)
Suriname	2 (1,4)	0.8 (0.29,1.62)	2.19 (0.91,3.89)	47 (17,95)	16.62 (6.09,33.09)	2.61 (1.12,4.5)
Sweden	100 (40,186)	0.87 (0.35,1.62)	3.62 (1.53,6.49)	1791 (748,3255)	16.38 (6.86,29.18)	4.24 (1.86,7.16)

Switzerland	53 (23,91)	0.58 (0.25,0.99)	3.04 (1.34,5.21)	992 (421,1683)	11.47 (4.89,19.52)	3.51 (1.58,5.79)
Syrian Arab Republic	17 (7,32)	0.31 (0.13,0.59)	3.26 (1.45,5.36)	413 (172,743)	6.41 (2.66,11.83)	3.82 (1.74,6.07)
Taiwan (Province of China)	66 (28,106)	0.34 (0.15,0.55)	3.12 (1.4,4.95)	1313 (572,2090)	6.66 (2.89,10.64)	3.47 (1.57,5.52)
Tajikistan	3 (1,6)	0.11 (0.04,0.22)	2.97 (1.26,5.14)	75 (28,152)	2.58 (0.95,5.12)	3.3 (1.43,5.61)
Thailand	141 (49,270)	0.29 (0.1,0.57)	2.88 (1.33,4.71)	3000 (1030,5788)	6.03 (2.07,11.54)	3.19 (1.49,5.12)
Timor-Leste	1 (0,2)	0.28 (0.11,0.5)	2.94 (1.25,4.74)	20 (8,36)	5.17 (1.96,9.16)	3.22 (1.36,5.04)
Togo	7 (2,14)	0.61 (0.21,1.15)	1.85 (0.76,3.27)	180 (61,355)	12.33 (4.26,24.07)	2.18 (0.93,3.74)
Tokelau	0 (0,0)	0.59 (0.24,1.09)	2.01 (0.85,3.68)	0 (0,0)	11.68 (4.68,20.81)	2.44 (1.06,4.35)
Tonga	0 (0,1)	1.38 (0.54,2.6)	3.51 (1.51,6.03)	9 (4,16)	25.04 (10.05,47.49)	3.93 (1.7,6.56)
Trinidad and Tobago	9 (4,16)	1.01 (0.41,1.85)	1.9 (0.82,3.24)	197 (83,351)	21.03 (8.77,37.96)	2.28 (1.02,3.75)
Tropical Latin America	688 (297,1217)	0.66 (0.28,1.17)	3.03 (1.34,5.4)	14239 (6252,24260)	12.74 (5.58,21.96)	3.39 (1.5,5.86)
Tunisia	20 (7,37)	0.36 (0.12,0.67)	4.66 (2.15,7.34)	407 (141,786)	6.74 (2.34,12.99)	5.1 (2.37,7.99)

Turkmenistan	3 (1,5)	0.18 (0.08,0.32)	3.67 (1.61,5.96)	76 (33,134)	4.21 (1.81,7.33)	4.11 (1.87,6.61)
Tuvalu	0 (0,0)	0.61 (0.25,1.16)	2.16 (0.92,3.71)	1 (0,1)	12.14 (5.08,22.89)	2.61 (1.13,4.34)
Turkey	208 (80,382)	0.55 (0.21,1.02)	3.37 (1.56,5.59)	4656 (1794,8389)	11.1 (4.26,20.22)	3.91 (1.85,6.31)
Uganda	20 (9,39)	0.41 (0.18,0.78)	0.74 (0.33,1.29)	500 (213,978)	8.51 (3.65,16.24)	0.85 (0.38,1.45)
Ukraine	192 (77,347)	0.65 (0.26,1.17)	4.24 (1.94,6.86)	4690 (1820,8550)	15.3 (5.96,27.91)	4.8 (2.21,7.68)
United Arab Emirates	4 (2,8)	0.4 (0.17,0.77)	2.95 (1.22,5.09)	128 (55,239)	7.1 (3.02,13.84)	3.19 (1.35,5.37)
United Kingdom	429 (182,808)	0.66 (0.28,1.23)	2.87 (1.22,5.29)	7917 (3432,14037)	12.53 (5.47,22.01)	3.31 (1.45,5.67)
United Republic of Tanzania	44 (16,87)	0.42 (0.15,0.83)	1.81 (0.77,3.05)	1067 (382,2157)	9.13 (3.25,18.34)	2.11 (0.93,3.52)
United States of America	1394 (581,2518)	0.51 (0.21,0.93)	3.09 (1.31,5.56)	35067 (15879,58801)	12.75 (5.76,21.32)	3.81 (1.69,6.52)
United States Virgin Islands	1 (0,1)	0.72 (0.23,1.64)	1.7 (0.63,3.44)	11 (4,25)	12.96 (4.11,29.79)	2 (0.77,3.89)
Uruguay	21 (9,38)	0.89 (0.38,1.59)	2.5 (1.13,4.2)	434 (193,759)	18.58 (8.25,32.47)	3.15 (1.48,5.17)
Uzbekistan	11 (5,18)	0.1 (0.05,0.18)	2.34 (1.03,3.73)	282 (120,462)	2.36 (1.01,3.9)	2.69 (1.2,4.25)

Vanuatu	0 (0,0)	0.36 (0.14,0.63)	1.43 (0.63,2.43)	6 (2,10)	7.19 (2.87,12.49)	1.73 (0.76,2.84)
Venezuela (Bolivarian Republic of)	62 (25,106)	0.51 (0.2,0.88)	1.56 (0.7,2.6)	1388 (562,2378)	10.46 (4.26,18.03)	1.75 (0.8,2.9)
Viet Nam	53 (17,91)	0.16 (0.05,0.28)	4.24 (1.95,6.81)	1226 (415,2109)	3.13 (1.03,5.37)	4.86 (2.24,7.66)
Western Europe	2574 (1122,4429)	0.56 (0.25,0.96)	3.05 (1.37,5.2)	50258 (22956,82734)	11.58 (5.34,18.87)	3.62 (1.66,5.89)
Western Sub-Saharan Africa	220 (75,401)	0.3 (0.1,0.55)	0.77 (0.34,1.33)	4978 (1686,9010)	5.93 (2.02,10.81)	0.92 (0.42,1.55)
Yemen	18 (6,34)	0.35 (0.13,0.67)	3.72 (1.69,6.19)	416 (149,770)	6.79 (2.4,12.69)	4.29 (1.94,7.02)
Zambia	11 (3,23)	0.39 (0.11,0.83)	0.94 (0.41,1.56)	289 (73,600)	9.16 (2.54,18.86)	1.09 (0.48,1.79)
Zimbabwe	35 (13,66)	1.55 (0.57,2.95)	2.63 (1.12,4.45)	841 (329,1567)	32.29 (12.14,61.27)	3.01 (1.28,5.1)

ASMR, age-standardized mortality rates; ASDR, age-standardized disability-adjusted life years rates; DALYs, disability-adjusted life years; PAF, population attributable fraction; ASM, age-standardized mortality; ASD, age-standardized disability-adjusted life year; UI, uncertainty interval.

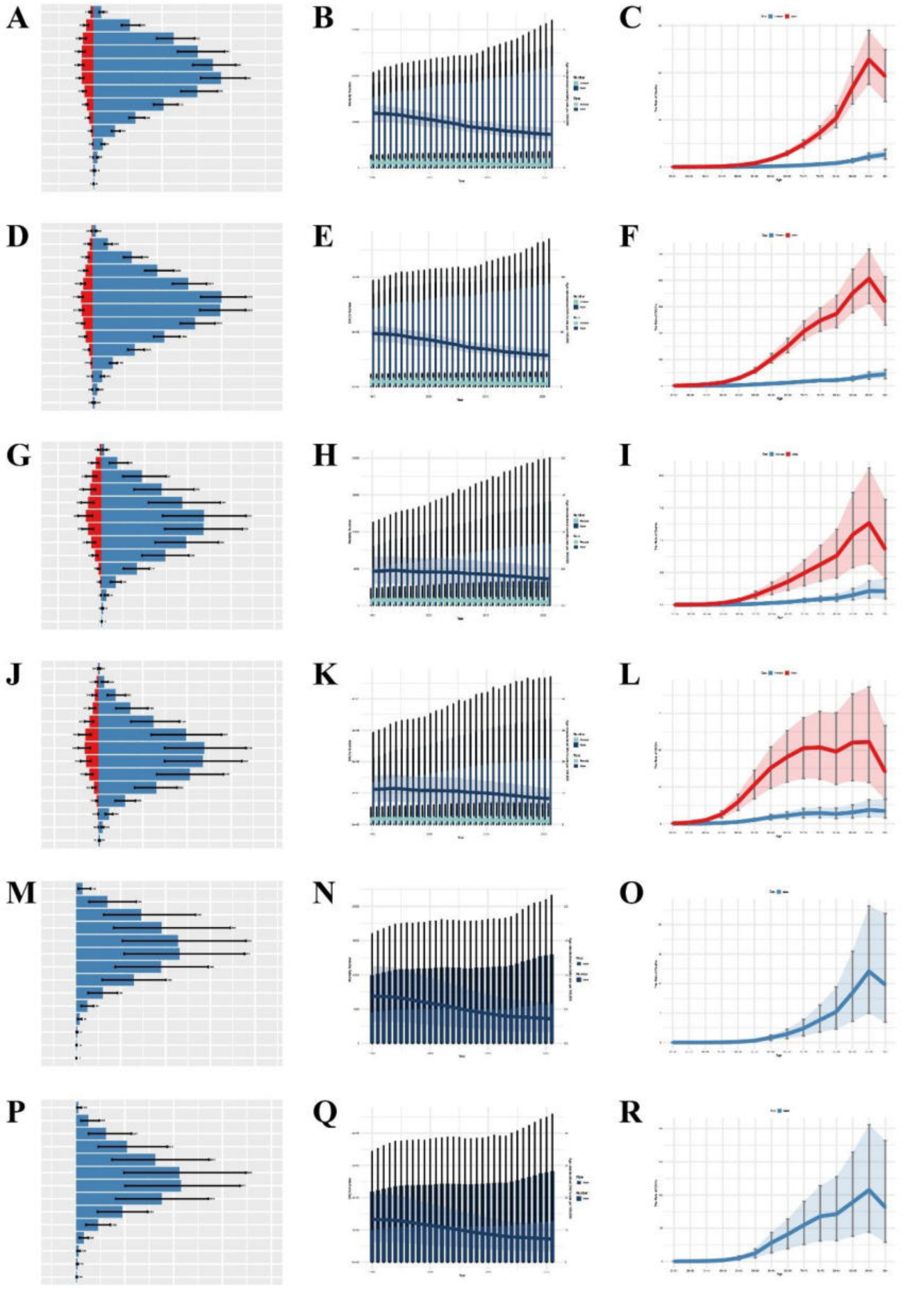

Supplementary Figure 1. The number of (A) bladder cancer deaths, (D) bladder cancer DALYs, (G) kidney cancer deaths, (J) kidney cancer DALYs, (M) PCa deaths, and (P) PCa DALYs in men or women by age. Temporal trends in (B) the number of deaths and ASMR in bladder cancer, (E) the number of DALYs and ASDR in bladder cancer, (H) the number of deaths and ASMR in kidney cancer, (K) the number of DALYs and ASDR in kidney cancer, (N) the number of deaths and ASMR

in PCa, and (Q) the number of DALYs and ASDR in PCa. Rates of (C) mortality for bladder cancer, (F) DALYs for bladder cancer, (I) mortality for kidney cancer, (L) DALYs for kidney cancer, (O) mortality for PCa, and (R) DALYs for PCa by age. DALYs, disability-adjusted life years; PCa, prostate cancer; ASMR, age-standardised mortality rate; ASDR, age-standardised DALYs rate.

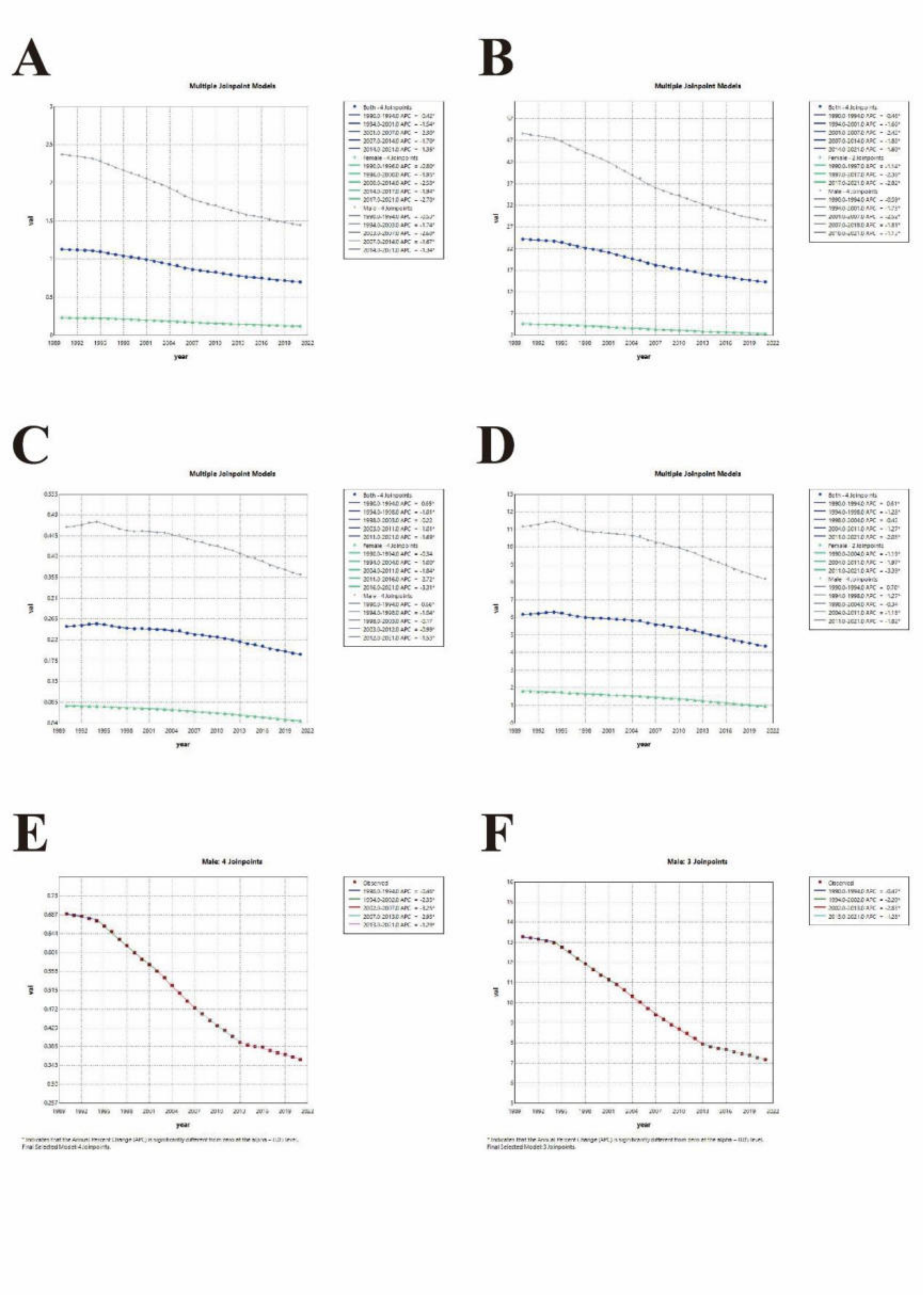

Supplementary Figure 2. The joinpoint regression analysis of (A) ASMR for bladder cancer, (B) ASDR for bladder cancer, (C) ASMR for kidney cancer, (D) ASDR for kidney cancer, (E) ASMR for PCa, (F) ASDR for PCa. ASMR, age-standardised mortality rate; ASDR, age-standardised disability-adjusted life years rate; PCa, prostate cancer.

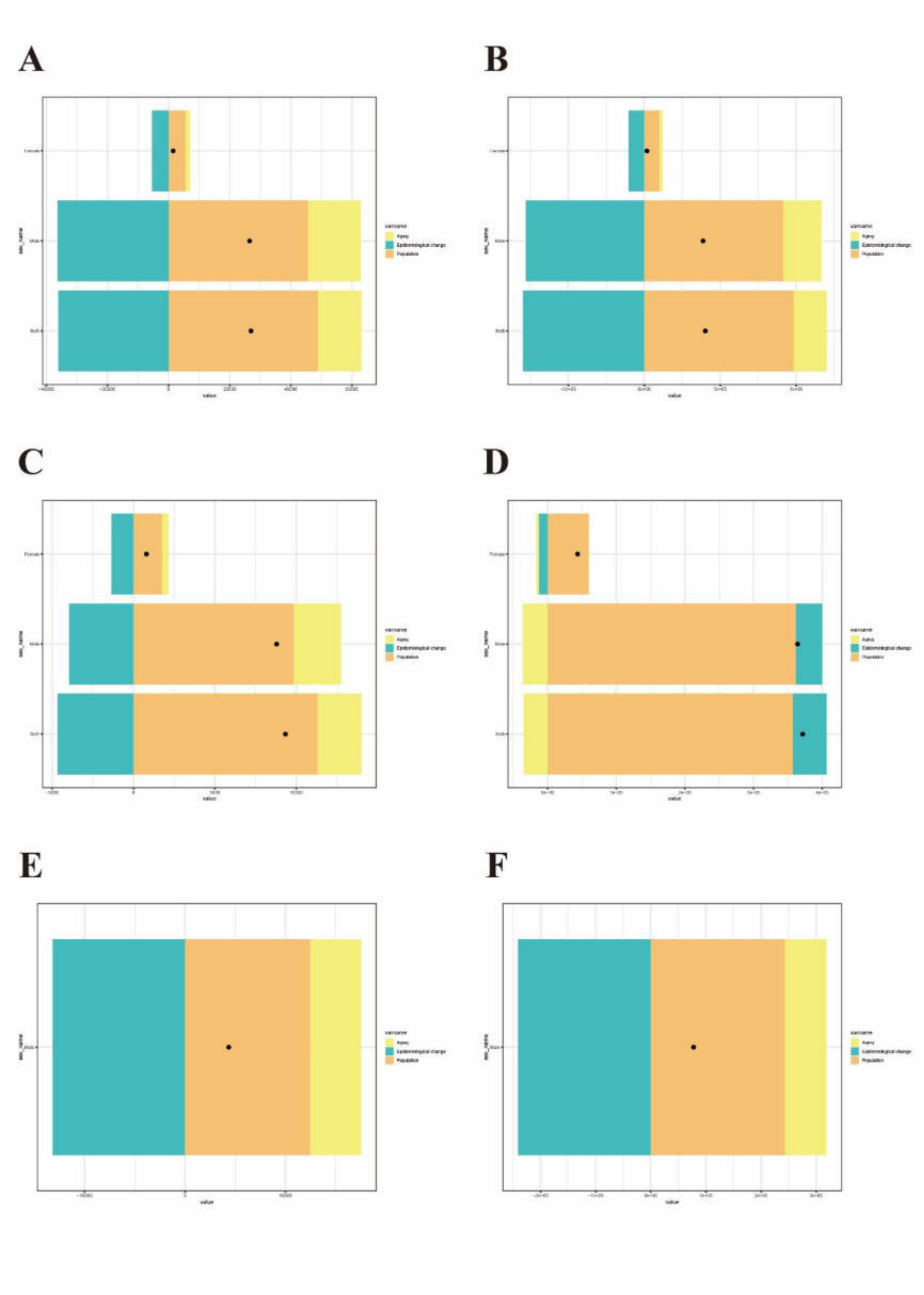

Supplementary Figure 3. Decomposition analysis of rates of **(A)** mortality in bladder cancer, **(B)** DALYs in bladder cancer, **(C)** mortality in kidney cancer, **(D)** DALYs in kidney cancer, **(E)** mortality in PCa, and **(F)** DALYs in PCa. DALYs, disability-adjusted life years; PCa, prostate cancer.

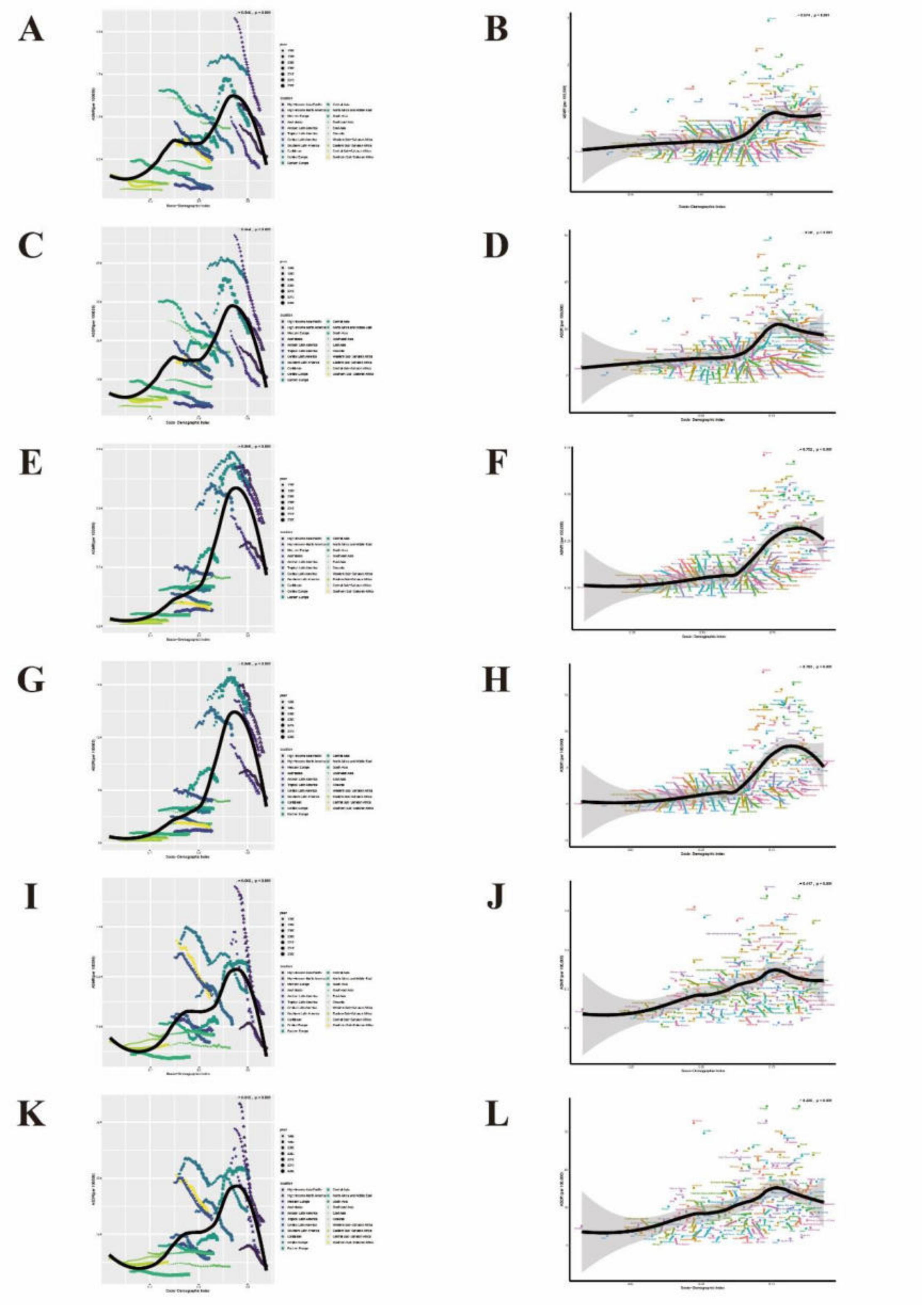

Supplementary Figure 4. The associations between the SDI and disease burden across GBD

regions or countries. (A) Association between ASMR and SDI in bladder cancer across GBD regions; (B) association between ASMR and SDI in bladder cancer across GBD countries; (C) association between ASDR and SDI in bladder cancer across GBD regions; (D) association between ASDR and SDI in bladder cancer across GBD countries; (E) association between ASMR and SDI in kidney cancer across GBD regions; (F) association between ASMR and SDI in kidney cancer across GBD countries; (G) association between ASDR and SDI in kidney cancer across GBD regions; (H) association between ASDR and SDI in kidney cancer across GBD countries; (I) association between ASMR and SDI in PCa across GBD regions; (J) association between ASMR and SDI in PCa across GBD countries; (K) association between ASDR and SDI in PCa across GBD regions; (L) association between ASDR and SDI in PCa across GBD countries. SDI, socio-demographic index; GBD, Global Burden of Disease; ASMR, age-standardised mortality rate; ASDR, age-standardised disability-adjusted life years rate; PCa, prostate cancer.

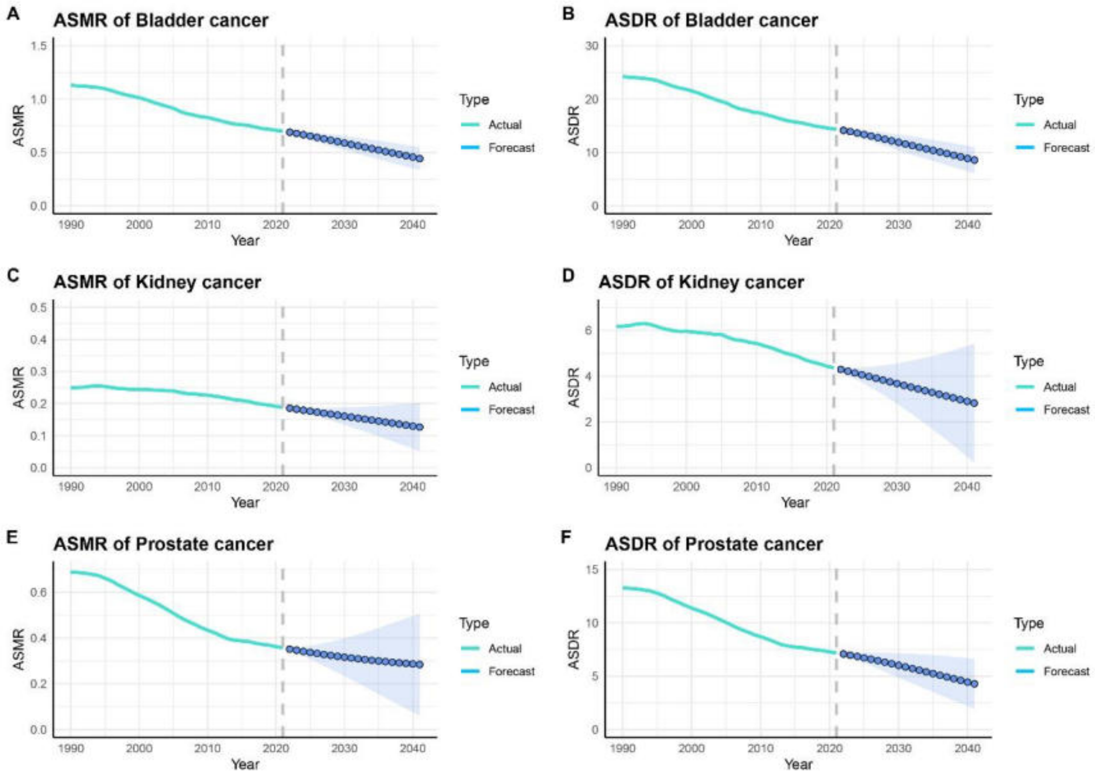

Supplementary Figure 5. The ARIMA model predicts (A) ASMR in bladder cancer, (B) ASDR in bladder cancer, (C) ASMR in kidney cancer, (D) ASDR in kidney cancer, (E) ASMR in PCa, and (F) ASDR in PCa over the next two decades. ARIMA, autoregressive integrated moving average; ASMR, age-standardised mortality rate; ASDR, age-standardised disability-adjusted life years rate; PCa, prostate cancer. Dark blue dot lines and shaded regions represent the predicted trend and its 95% confidence interval (CI).

© 2025 Chen X. et al.
